# Solvent Cavitation during Ambient Pressure Drying of Silica Aerogels

**DOI:** 10.1021/acs.langmuir.4c00497

**Published:** 2024-06-12

**Authors:** Julien Gonthier, Ernesto Scoppola, Tilman Rilling, Aleksander Gurlo, Peter Fratzl, Wolfgang Wagermaier

**Affiliations:** † Department of Biomaterials, Max Planck Institute of Colloids and Interfaces, 14476 Potsdam, Germany; ‡ Chair of Advanced Ceramic Materials, Institute of Materials Science and Technology, Faculty III Process Sciences, Technische Universität Berlin, 10623 Berlin, Germany

## Abstract

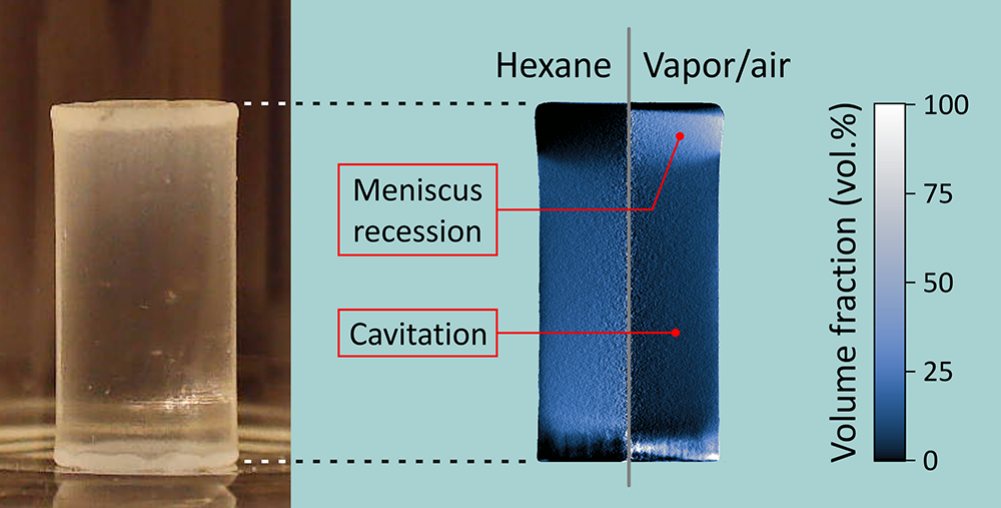

Ambient-pressure drying of silica gels stands out as an economical and accessible process for producing monolithic silica aerogels. Gels experience significant deformations during drying due to the capillary pressure generated at the liquid–vapor interface in submicron pores. Proper control of the gel properties and the drying rate is essential to enable reversible drying shrinkage without mechanical failure. Recent in operando microcomputed X-ray tomography (μCT) imaging revealed the kinetics of the phase composition during drying and spring-back. However, to fully explain the underlying mechanisms, spatial resolution is required. Here we show evidence of evaporation by hexane cavitation during the ambient-pressure drying of silylated silica gels by spatially resolved quantitative analysis of μCT data supported by wide-angle X-ray scattering measurements. Cavitation consists of the rupture of the pore liquid put under tension by capillary pressure, creating vapor bubbles within the gels. We found the presence of a homogeneously distributed vapor-air phase in the gels well ahead of the maximum shrinkage. The onset of this vapor/air phase corresponded to a pore volume shrinkage of ca. 50 vol % that was attributed to a critical stiffening of the silica skeleton enabling cavitation. Our results provide new aspects of the relation between the shape changes of silica gels during drying and the evaporation mechanisms. We conclude that stress release by cavitation may be at the origin of the resistance of the silica skeleton to drying stresses. This opens the path toward producing larger monolithic silica aerogels by fine-tuning the drying conditions to exploit cavitation.

## Introduction

Aerogels are porous materials with high specific surface area and submicrometer pore size.^1,2^ They consist of a solid matrix of particles or fibers filled by an open network of air-filled pores making up 80–99% of the total volume, thus the name aerogel.^3^ Silica-based aerogels display among the lowest thermal conductivities observed in solids,^4,5^ making them competitive in thermal insulation.^6,7^ Aerogels are obtained by replacing the pore liquid of a gel with air while conserving the native matrix and pore structure, which is achieved by drying. Ambient-pressure drying (also called evaporative drying) stands out as a safe and economically attractive process to produce aerogels granulates, composites, or monoliths compared to more energy-intensive techniques like supercritical drying.^8,9^ Understanding, predicting, and potentially tailoring the evaporation mechanisms in gels is crucial as they impact the structure and performance of the aerogels. Previous work revealed the kinetics of the evaporative drying process in terms of global phase composition, giving insights into the conditions of emergence and dynamic of the aerogel spring-back.^10^ To elucidate the actual evaporation mechanisms and differentiate between evaporation by meniscus recession, drying shrinkage, and cavitation, we modeled the spatial distribution of the pore liquid and gas in the gels throughout drying.

**Figure 1:**
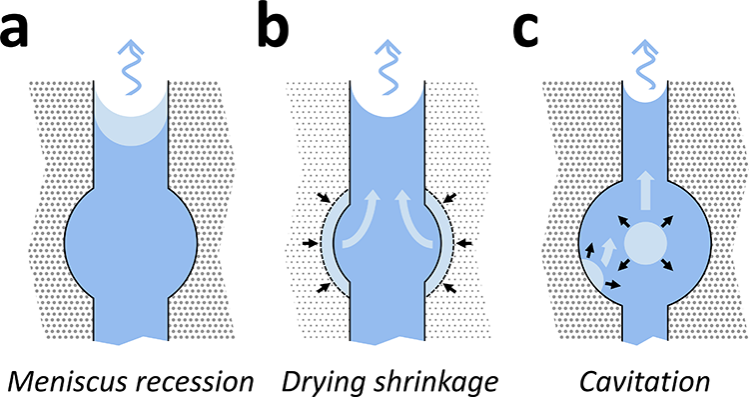
Illustration of the three evaporation mechanisms in a simplified cylindrical pore with an ink-bottle-shaped geometry confined by a solid matrix (in gray). (a) Evaporation by recession of the meniscus. (b) Evaporation by drying shrinkage. (c) Evaporation by homogeneous (center bubble) and heterogeneous (edge bubble) cavitation. The black arrows depict the tension in the liquid and the light blue arrows indicate the liquid flow. The gray background represents the stiffness of the matrix, a denser background stands for a higher stiffness. The wavy arrow on top illustrates the evaporated liquid.

In general, evaporation of a liquid confined in a porous media can occur by three mechanisms: evaporation by recession of the liquid–vapor interface, drying shrinkage, and cavitation (Figure 1). Drying starts with the formation of a meniscus at the outer surface of the pores. This liquid–vapor interface is subject to capillary pressure and the tension of the liquid confined to a cylindrical pore of radius *r*_c_ can be calculated with the Young–Laplace equation:^11,12^
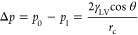
1where Δ*p* is the capillary pressure, *p*_0_ is the vapor pressure, *p*_l_ is the pressure in the liquid, γ_LV_ is the surface tension of the liquid–vapor interface, and θ is the contact angle of the meniscus. For wetting fluids (θ < 90°), *p*_l_ can be negative: the liquid is under tension.^13,14^ Let us consider a liquid fully wetting the solid (θ = 0, which is true for most solvent-gel systems^15^). Evaporation by the recession of the meniscus will occur, provided that the tension in the liquid remains under the elastic limit of the solid matrix. This is typically the case for porous materials with a large pore size or a stiff matrix. With water as the pore liquid (γ_water_≈ 72 mN m^–1^) and a pore size of 1 μm, the capillary pressure given by eq 1 would be around 0.15 MPa, which is about the yield strength of polyurethane foams^16^ (found in, e.g., kitchen sponges). The meniscus recedes in the larger pores first, resulting in a heterogeneous drying front inside the porous medium where evaporation is limited by the diffusion of the vapor phase.^17,18^ Under certain conditions, evaporation by the recession of the meniscus may also proceed by a sudden conversion of liquid to vapor referred to as adiabatic burst events.^19^

Evaporation by drying shrinkage takes place in porous materials with a compliant matrix and a smaller pore size, eventually preceded by an initial stage of evaporation by meniscus recession. As the meniscus reaches smaller pores, the tension in the liquid increases and can overcome the elastic limit of the solid matrix, which then contracts onto the liquid.^20^ The flow of liquid resulting from the pore volume shrinkage sustains the evaporation at the meniscus through poromechanical coupling.^21^ The capillary pressure can be as high as 150 MPa for a pore radius of 1 nm (with eq 1, considering water), resulting in a significant compressive stress on the solid matrix.

Evaporation by cavitation may take place in porous materials with a stiff matrix and “ink-bottle″-shaped pores, that is, large pores constricted by smaller pore necks.^22^ Unlike evaporation by drying shrinkage, the tension in the liquid is not compensated by a contraction of the solid matrix and keeps rising, making the liquid metastable and susceptible to cavitation. Cavitation occurs by the nucleation of vapor bubbles in larger pores ahead of the pore constriction, where the effects of confinement by the matrix are less strong.^23−25^ The evaporation proceeds at the meniscus and is sustained by the liquid flow toward the pore constriction at the expense of the formation and growth of the bubbles. The onset of cavitation depends on the state of the liquid: its saturation vapor pressure, surface tension, and temperature;^26−29^ and on the properties of the porous media: the pore size distribution,^22,26,27,30^ the stiffness of the solid matrix,^23,25^ and the presence of defects.^31^

Evaporative drying of gels is commonly described as a succession of evaporation by drying shrinkage and meniscus recession.^15^ The drying stress is reduced by replacing the pore liquid of the gels before drying with a liquid with a low surface tension such as *n*-hexane.^32^ Because the matrix of the gels is initially rather compliant, evaporative drying first proceeds by drying shrinkage, and the menisci remain mostly located on the outer surface of the gel. The decrease in the pore volume causes a progressive stiffening of the gel. As the tension in the liquid increases, it reaches a maximum once the radius of curvature of the meniscus becomes equal to the radius of the smallest pores. At this point, the liquid tension cannot overcome the stiffening of the solid matrix, marking the end of drying shrinkage and the beginning of evaporation by meniscus recession. This threshold is known as the maximum shrinkage or critical point of drying where the gel volume can reach about 20% of its original volume.^33,34^ As the meniscus recedes into the pores, the compressive stress on the solid matrix related to capillary forces is released. In silica gels modified with a silylating agent such as trimethylchlorosilane (TMCS), this coincides with a re-expansion of the solid matrix, enabling recovery of the drying shrinkage known as the spring-back effect (SBE) and resulting in hydrophobic aerogels.^1^ The SBE is a key feature in the production of aerogels by evaporative drying and its origin has not been investigated until recently.^10,35−38^ Nevertheless, it is not yet understood how monolithic silica aerogels can be produced by evaporative drying considering the extreme stress exerted on the silica solid matrix by drying shrinkage and spring-back, which therefore challenges the current models of evaporative drying.

In addition to the two known evaporation mechanisms, it is theoretically possible that cavitation also plays a central role during the drying of gels at ambient pressure as suggested by Scherer & Smith.^39^ Using classical nucleation theory (CNT), they estimated that homogeneous nucleation of vapor bubbles may happen before maximum shrinkage provided a small enough pore size (1–2 nm). Cavitation events are particularly intriguing in gels as they stabilize the tension in the liquid, which in turn would reduce the stress on the silica matrix and potentially prevent mechanical failure. A systematic literature review of publications citing the Scherer & Smith paper revealed the absence of experimental evidence of cavitation events in the drying of gels to produce aerogels. To our knowledge, there have been only three studies reporting visual observations of an opaque phase growing in the core of gels dried at ambient pressure that could possibly be associated with cavitation bubbles.^40−42^ The lack of investigation on cavitation is presumably due to experimental limitations: monitoring the evaporative drying of gels requires nondestructive in operando methods at ambient pressure and with enough resolution and/or contrast to resolve cavitation events.

We have recently reported that monolithic silica aerogels dried at ambient pressure contained up to 37 vol % of gas at the maximum shrinkage using an X-ray microcomputed tomography (μCT) quantitative imaging procedure.^10^ This is inconsistent with a drying model based on dual-evaporation mechanisms although the spatial distribution of gas was not evaluated. This finding echoed with the theoretical study of Scherer & Smith and motivated the current work with the aim of evaluating cavitation as an additional evaporation mechanism. μCT records changes in the absorption of an irradiated specimen and allows the reconstruction of a 3D volume, in which the contrast is proportional to the attenuation coefficient of the specimen.^43^ Quantitative imaging of the reconstructed volumes correlates the temporal variations in the attenuation coefficients of a specimen with changes in composition by image processing and subsequent modeling. Spatially resolved quantitative imaging can produce composition maps notably showing the distribution of vapor/air inside of the gels and provide information on the evaporation mechanisms. Besides absorption, changes in gels’ composition can also be evaluated from the scattering of X-rays upon irradiation of a gel. In the wide-angle X-ray scattering (WAXS) region, the scattering signal arises from the molecular structure of the pore liquid and solid silica skeleton.^35,44^ Deconvolution of these two signals and subsequent modeling allow us to calculate the average phase composition of the gels within the incident beam path.

In this work, previously published μCT data on the drying of silica gels^10^ were reanalyzed based on a spatially resolved μCT quantitative imaging workflow to test for the hypothesis of cavitation. While our precedent quantitative imaging workflow only allowed us to calculate the average phase composition, the procedure presented in this study generates composition maps of pore liquid and vapor/air within silica gels during drying. This computational approach consists in reducing and interpolating 4D reconstructed volumes, along with a systematic evaluation of instrumental and computational artifacts. Despite the resolution of the μCT scans (11 μm) being much larger than the size of the cavitation bubbles as postulated by Scherer & Smith (2 nm), the present approach was able to capture the spreading of a vapor/air phase in the gels well ahead of the maximum shrinkage. That gas phase appeared and grew homogeneously across the sample, suggesting it was created by cavitation of the solvent. In addition, we performed WAXS measurements and CNT estimations, which consolidated the results on the evaporation mechanisms. This study reports indirect evidence of evaporation by cavitation in silica gels by two methods, opening new aspects to the understanding and improvement of the evaporative drying process to produce high-performance, monolithic aerogels.

## Experimental Section

### Materials

Five silylated silica gels, previously analyzed to study the kinetics of their average phase composition in ref (10), were reused in this work with a new image processing approach. They were synthesized by a two-step sol–gel process adapted from refs (35,45). A silica sol was prepared from a tetraethyl orthosilicate (TEOS) precursor and ethanol and was cast in cylindrical molds to produce gels of 16 and 8 mm in height and diameter, respectively. The resulting gels underwent a solvent exchange for *n*-hexane, followed by a surface modification with trimethylchlorosilane (TMCS) in *n*-hexane and a final solvent exchange for *n*-hexane. The five silylated silica gels were labeled M1–M5.

### In Operando μCT

The evaporative drying of gels M1–M5 was monitored by taking a series of μCT scans as described in our recent work.^10^ Each scan was reconstructed and segmented using semiautomatic procedures to generate masked slices of the specimen and to calculate morphological information. This was done by transferring a gel from its *n*-hexane storage solution to a tailored PEEK drying chamber. The chamber has an open lid through which the vapors exit. The chamber was placed in an EasyTom 160/150 CT system (RX Solutions, Chavanod, France), and 141 μCT scans were acquired in the step and shot mode without reference images at a voxel size of 11 μm to monitor the drying process in operando, which lasted between 14 and 16 h. The projections were reconstructed along the samples’ height using a cone-beam algorithm in the software XAct (RX Solutions) to generate a set of slices (8-bit tif images). The segmentation of the reconstructed volumes was carried out in the software Dragonfly^46^ using the Python console and a region of interest (ROI) labeling the sample at each scan was created.

The ROIs were used to overwrite the gray values of all background voxels in the slices by zero, generating masked slices that were exported as 8-bit tif images. For a single sample, 141 series of 1000+ masked slices were created, corresponding to the segmented volume of the gel over time. The total volume of the gel at each scan was given by the total volume of the corresponding ROI: *V*_*k*_, where the index *k* refers to the scan number: 0 ≤ *k* ≤ 140 and . The gels volume was corrected as described in our previous work.^10^ The drying time was defined as the difference between the mean time of each scan (taken as the average of the timestamps of the 64 projections) and the time at which the lid of the drying chamber was opened. In addition to the five silica gels, a reference liquid *n*-hexane sample was scanned under the same in operando conditions. The masked images were then processed using a quantitative imaging workflow consisting of two parts: data reduction and modeling.

### μCT Data Reduction

**Figure 2:**
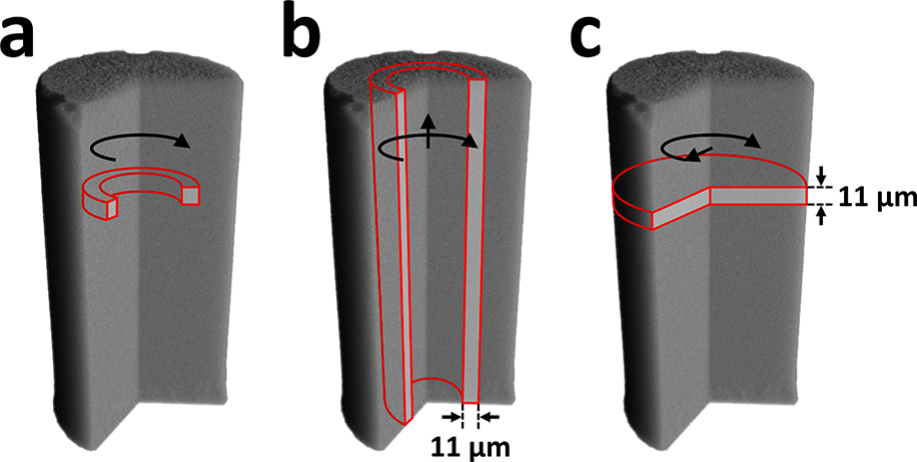
Illustration of the three data reduction procedures on the 3D segmented volume of gel M4 at the beginning of drying. (a) Azimuthal integration (GHR maps). (b) Azimuthal + vertical integration (GR map). (c) Slice integration (GH map). The black arrows indicate the direction of integration, and the red contours depict an integrated volume element. The integration step was equal to the voxel size: 11 μm.

The motivation behind the reduction of the reconstructed μCT data was to simplify the geometry of the gels during drying, taking advantage of their cylindrical symmetry. Doing so allowed us to work on a 2D or 3D data set instead of a 4D data set (three spatial dimensions and one temporal dimension). It also improved the presentation of the results that can be shown against different axes of the cylinders independently. The time series of masked images was reduced by integrating the data over one or more axes of the samples. The raw gray values in the masked images were corrected for the anode heel effect,^10,47^ resulting in the gray values *g*_*i*, *p*, *q*, *k*_ with *i*, *p*, *q* the *z*, *x*, *y* coordinates in the reconstructed volume, respectively, and *k* the time index. . Formally, *i* = *i*_*k*_, *p* = *p_k_,* and *q* = *q*_*k*_ because the shape of the sample was changing over time. Three reduction procedures were used to generate different spatial and temporal representations of the reconstructed gray values during drying (Figure 2):

a) Azimuthal integration (3D, Figure 2a): the masked slices were integrated over the azimuth of the cylinder, resulting in 141 2D gray value maps along the height and radius of the cylinder: *g*_*i*, *p*, *q*, *k*_ → *g*_*i*, *j*, *k*_, where *j* represents the radial distance to the center of a masked slice. . The domain of pixels in the maps belonging to the sample was referred to as Ω_*k*_ with (*i*, *j*) ∈ Ω_*k*_. This representation was referred to as the maps of the gray values along the height and radius (GHR maps).
b) Azimuthal and vertical integration (2D, Figure 2b): the GHR maps were further integrated over the vertical axis of the cylinder resulting in a single gray value map along the radius of the gel and the scan number: *g*_*i*, *j*, *k*_ → *g*_*j*, *k*_. *j* ∈ Ω_*k*_^*r*^ where Ω_*k*_^*r*^ is the domain of pixels in the map belonging to the sample. This representation was referred to as the radial gray value map (GR map).
c) Slice integration (2D, Figure 2c): the masked slices were integrated over *p* and *q*, resulting in a single gray value map along the height of the gel and the scan number: *g*_*i*, *p*, *q*, *k*_ → *g*_*i*, *k*_. *i* ∈ Ω_*k*_^*h*^ where Ω_*k*_^*h*^ is the domain of pixels in the map belonging to the sample. This representation was referred to as the vertical gray value map (GH map).

A complete description of the reduction procedures can be found in the SI1. The conversion between a gray value and the corresponding reconstructed attenuation coefficient (RAC) was given by
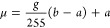
2where μ is the RAC, and *a* and *b* are the custom contrast parameters used for reconstructing the μCT projections. After data reduction, RAC maps were generated using eq 2 and referred to as MHR, MR, and MH maps. These data were then used to model the phase composition of the drying silica gels.

### μCT Drying Model

The spatial and temporal phase composition of the drying gels was computed by applying a drying model to the reduced data adapted from our previous study.^10^ The construction of the drying model consists in (1) derive the main equations, (2) make assumptions to express additional equations, and (3) solve the system of equations using bilinear interpolation and correction factors. The model presented in this section applies to the MHR maps resulting from the azimuthal integration, but the notation can easily be extended to the two other data reduction procedures. At any time during drying, the silica gels were composed of three phases: solid silica skeleton, liquid *n*-hexane, and vapor/air. In the MHR maps, the RAC can thus be written as
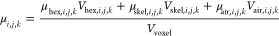
3where μ_φ, *i*, *j*, *k*_ and *V*_φ, *i*, *j*, *k*_ are the RAC and volume of a phase φ, respectively, at a vertical and radial coordinate (*i*, *j*) and scan *k* for the hexane, skeleton, and vapor/air phases, and *V*_voxel_ is the volume of a voxel. The attenuation of the vapor/air phase was set to zero; thus: μ_air, *i*, *j*, *k*_ = 0. The RAC of the silica skeleton and liquid *n*-hexane phases was assumed constant and homogeneous throughout drying: μ_hex, *i*, *j*, *k*_ = μ_hex_ and μ_skel, *i*, *j*, *k*_ = μ_skel_. μ_hex_ was calculated from the reference *n*-hexane measurements as described in ref,^10^ μ_hex_ = 0.155. Eq 3 can be rewritten as a function of the volume fraction of each phase at a given voxel rather than the total volume, leading to the first equation of the drying model:
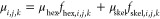
4where *f*_φ, *i*, *j*, *k*_ = *V*_φ, *i*, *j*, *k*_/*V*_voxel_ for a phase φ. Additionally, the volume conservation implies
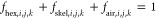
5

The volume fraction maps were referred to as the HEXHR, SKELHR, and AIRHR maps for *f*_hex, *i*, *j*, *k*_, *f*_skel, *i*, *j*, *k*_, and *f*_air, *i*, *j*, *k*_, respectively. Solving the system of equations required making assumptions about the composition of the gels throughout drying. First, the HEXHR maps were computed by assuming that the hexane content was zero at the end of drying. To do so, a RAC map representative of the dry gel was generated:
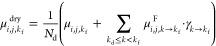
6where μ_*i*, *j*, *k*_f__^dry^ is an artificial MHR map defined over the domain Ω_*k*_f__, *k*_f_ = 140 is the final scan, *k*_d_ is a threshold scan number from which the hexane content is assumed to be zero, *N*_*d*_ is the number of scans in *k*_*d*_ ≤ *k* ≤ *k*_*f*_, μ_*i*, *j*, *k* → *k*_*f*__^*F*^ is a map generated by bilinear interpolation of a map from scan *k* ≥ *k*_d_ onto the domain of the map at scan *k* = *k*_*f*_, and γ_*k*→*k*_*f*__ is a scaling factor. The scaling factor was defined as the volume ratio between a source scan *k*_1_ and a target scan *k*_2_ as γ_*k*_1_ → *k*_2__ = *V*_*k*_1__/*V*_*k*_2__. Bilinear interpolation was required so that a map defined over a domain Ω_1_ could match the domain Ω_2_ of another map because the change in the sample volume throughout drying implied that the domain Ω_*k*_ was different for all scans. The map μ_*i*, *j*, *k*_f__^dry^ was then interpolated from source scan *k*_*f*_ onto target scan 0 ≤ *k* < 140, resulting in the maps μ_*i*, *j*, *k*_*f*_ → *k*_^dry,F^ defined over Ω_*k*_ at any scan. The HEXHR maps of any scan could be computed by replacing the product μ_skel_*f*_skel, *i*, *j*, *k*_ in eq 4 by μ_*i*, *j*, *k*_*f*_ → *k*_^dry, F^ with a scaling factor:
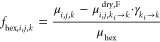
7

The SKELHR maps were computed by assuming a zero vapor/air content at the beginning of drying from scan number *k*_1_ to *k*_2_. The maps at *k*_1_ < *k* ≤ *k*_2_ were rescaled onto the domain of target scan *k*_1_ and were then averaged over *k*_1_ ≤ *k* ≤ *k*_2_ to compute an artificial map representative of the alcogel μ_*i*, *j*, *k*_1__^alco^:
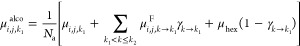
8where μ_*i*, *j*, *k* → *k*_1__^*F*^ is a map interpolated from scan *k*_1_ < *k* ≤ *k*_2_ to scan *k* = *k*_1_ and *N*_a_ is the number of scans in *k*_1_ ≤ *k* ≤ *k*_2_. *k*_d_, *k*_1_, and *k*_2_ were evaluated using a global quantitative imaging approach and were reported in ref (10). The rightmost product in eq 8 was an additional scaling factor. An artificial hexane map representative of the alcogel at scan *k* = *k*_1_ was computed as
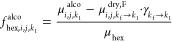
9

The skeleton map at *k* = *k*_1_ was then calculated by substituting *f*_hex, *i*, *j*, *k*_ by *f*_hex, *i*, *j*, *k*_1__^alco^ in eq 5, resulting in *f*_skel, *i*, *j*, *k*_1__. The SKELHR maps for the other scans were computed by interpolating and rescaling the SKELHR map from scan *k*_1_ to scan *k* ≠ *k*_1_:
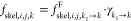
10where  is the skeleton map interpolated from source scan *k* = *k*_1_ to target scan *k* ≠ *k*_1_. The AIRHR maps were finally calculated with eq 5. The complete derivation of the drying model is reported in SI2 and the bilinear interpolation algorithm is reported in SI3. Sample M3 had a large meniscus at its bottom (Figure S5), leading to complications in the data reduction procedure, and was thus discarded. Similar equations were derived for the MR and MH maps (not shown), and the resulting volume fraction maps were referred to as the HEXR, SKELR, and AIRR and HEXH, SKELH and AIRH maps, respectively. The GR and GH maps and the corresponding volume fraction maps were interpolated from scan number to time to create profiles monotonically increasing with time. Finally, all maps were saved as 2D float arrays, which were then converted to images to create the figures. The data processing, modeling and the creation of the figures were carried out in Python with the DipLib, Matplotlib, NumPy, Pillow, and Scipy libraries.^48−52^

### In Operando X-ray Scattering

X-ray scattering experiments were performed to monitor the structure of the gels during drying at a wide angle, which corresponds to the molecular structure of the silica skeleton and *n*-hexane. The general idea was to correlate the evolution of scattering intensity in the wide-angle region with the change in the specimen composition within the volume probed by the beam. X-ray scattering measurements were performed at the BESSY II synchrotron of the Helmholtz-Zentrum für Materialien and Energie (Germany, Berlin) at the μSpot beamline.^53^ One silylated silica gel (labeled M6) was dried at ambient pressure in a tailored measurement cell adapted from ref (35). The cell was constructed from anodized aluminum with a silicon wafer and a silicon nitride window (NORCADA low-stress SiNx membrane, 10 mm length/width, 1000 nm thickness) placed in the direction of the X-ray beam. The top of the cell was sealed with a valve (1/8 in., PN63/1.4408, shortened with an adapter to ca. 26 mm), and a museum glass was placed on the side to allow the collection of digital pictures of the sample with a digital microscope camera (TOOLKRAFT USB microscope, 5 MP). The cell was mounted on a rotary stage that could host up to five cells. At the time of measurement, sample M6 was transferred from its *n*-hexane storage solution to the measurement cell, whereas an empty cell was used for background correction. The valve was opened fully before the measurement.

Experiments were performed using a monochromatic X-ray beam at 18 keV and a B4C/Mo Multilayer (2 nm period) monochromator. A spot size of 30 × 30 μm^2^ was adjusted by a series of pinholes. The cell position was set so that the beam hits the sample at a fixed location, 4 mm from the bottom of the sample. The scattering data were collected on an Eiger 9 M detector with a 75 × 75 μm^2^ pixel size. A quartz reference was fixed at the same distance from the beam source as the sample and was used to determine the sample–detector distance, beam center, tilt, and rotation. A glassy carbon Standard Reference Material 3600 (SRM 3600) of the National Institute of Standards and Technology (NIST) was measured for absolute intensity calibration.^54^ The transmission through the sample was calculated from the X-ray fluorescence signal collected from a lead beamstop by using a RAYSPEC Sirius SD-E65133-BE-INC detector equipped with an 8 μm beryllium window, while the primary beam intensity was monitored and normalized by using an ion chamber. Each data frame was collected by exposing the sample to radiation for 1 s every 27 s, with the rotary stage alternating between an empty cell and the sample cell. The resulting data were preprocessed/previewed using the DPDAK software package^55^ and a custom Python script utilizing the pyFAI library.^56^ The preprocessing steps involved integration to 1D scattering curves and subtraction of an instrumental background (i.e., the empty cell). The scattering data were corrected for transmission and primary beam intensity and corrected for a “container background”. To normalize the data with the sample thickness, the diameter of the gel was determined from the optical images collected during drying (see SI4). As a final step, data were scaled to absolute units (i.e., cm^–1^) by sample thickness normalization and by the scaling factor of the glassy carbon.

The azimuthal integration of X-ray scattering measurements provided scattered intensity *I*(*q*) as a function of the momentum transfer *q* = 4π sin (θ/2)/λ, using the wavelength of the synchrotron beam λ and the scattering angle θ, resulting in an accessible *q* range of ca. 0.07 to 40 nm^–1^. Collected data were analyzed in a wide-angle diffraction region (3–30 nm^–1^) to obtain time-dependent volume fraction profiles. To this end, diffraction data of liquid hexane in a borosilicate glass capillary were collected. The latter was reduced following the same procedure described above (i.e., monitor and transmission normalization, empty capillary subtraction, radial integration) but not scaled by sample thickness or corrected by glassy carbon scaling factor.

To compute the hexane, skeleton and vapor/air volume fraction profiles of the gel at each data frame, the diffraction 1D profiles of the hexane reference: *I*_hex_(*q*, *t*) and of the dry aerogel (i.e., last collected data frame): *I*_dry_(*q*, *t*) were modeled using a baseline function and a function sum of three pseudovoigts:
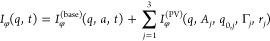
11where *I*_φ_(*q*, *t*) stands for *I*_hex_(*q*, *t*) or *I*_dry_(*q*, *t*) and the parameters *A*_*j*_, *q*_0, *j*_, Γ_*j*_, *r*_*j*_ of the pseudovoigt represent the area, the center, the full width at half-maximum and the Gaussian–Lorentzian ratio, respectively. Similarly to the μCT drying model, the hexane content in the gel at the end of drying was assumed to be zero, so that the scattered intensity of the dry gel arose only from the skeleton and vapor/air. The baseline function for the hexane reference: *I*_hex_^(base)^(*q*, *a*, *t*) was set as a linear polynomial with slope *a*:
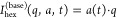
12

and the baseline function for the dry aerogel was set as a power law decay with a constant *a* representing the law’s exponent:
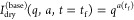
13where *t* = *t*_f_ is the time of the last data frame. During the modeling of the hexane and dry aerogel diffraction profiles, an additional parameter representing the data background was used. To obtain volume fraction profiles of each phase, two more steps were necessary. At first, the volume fraction of the skeleton in the dry aerogel was calculated by assuming a composition: Si_23_O_40_C_9_H_28_ and a skeletal density of approximately 1.9 g cm^–3^. That composition was estimated by comparing the weight of fully dried silylated gels with fully dried unmodified gels^10^ and by assuming that the weight difference was only due to the silyl groups in the modified gels: Si(CH_3_)_3_. Moreover, the unmodified gels were left in a desiccator for 24 h before being weighed to complete drying.

Subsequently, by means of the Python library xraylib^57,58^ and the Beer–Lambert equation, it was possible to compare the experimental transmission of the dry aerogel *T*_skel_ and the volume fraction-dependent theoretical transmission:
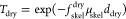
14with  the skeleton volume fraction of the dry gel, μ_skel_ its attenuation coefficient for an 18 keV X-ray beam, and *d*_dry_ the sample diameter obtained with the optical microscope at the corresponding time. The model functions *I*_hex_(*q*), *I*_dry_(*q*) and  were combined and used for fitting the time-dependent scattering profiles *I*(*q*, *t*) of the drying gel as follows:
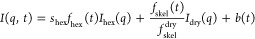
15

with *f*_hex_(*t*) and *f*_skel_(*t*) the hexane and skeleton time dependent volume fractions, *b*(*t*) a background independent of *q*, and *s*_hex_ a constant factor to scale the hexane data to absolute units (i.e., cm^–1^). *s*_hex_ was calculated by assuming that at *t* = 0, the vapor/air content in the gel was zero, leading to
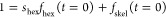
16

At *t* > 0, the time dependent vapor/air volume fraction *f*_air_(*t*) was calculated by modifying eq 16:
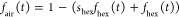
17

which implies that *f*_air_(*t* = 0) = 0. All data modeling was performed using the Scipy optimize library provided by Python.^52^ In order to improve performance and result reliability, fits were performed by providing analytical functions and jacobians. Best-fit parameter uncertainties were therefore calculated by evaluating the Jacobian at the minimum of the penalty function distribution.

Figures were generated using the scientific color maps batlow, lapaz, and oslo^59^ to prevent visual distortion of the data and exclusion of readers with color-vision deficiencies.^60^

## Results and Discussion

### Hexane and Vapor/Air Spatial and Temporal Distributions

**Figure 3:**
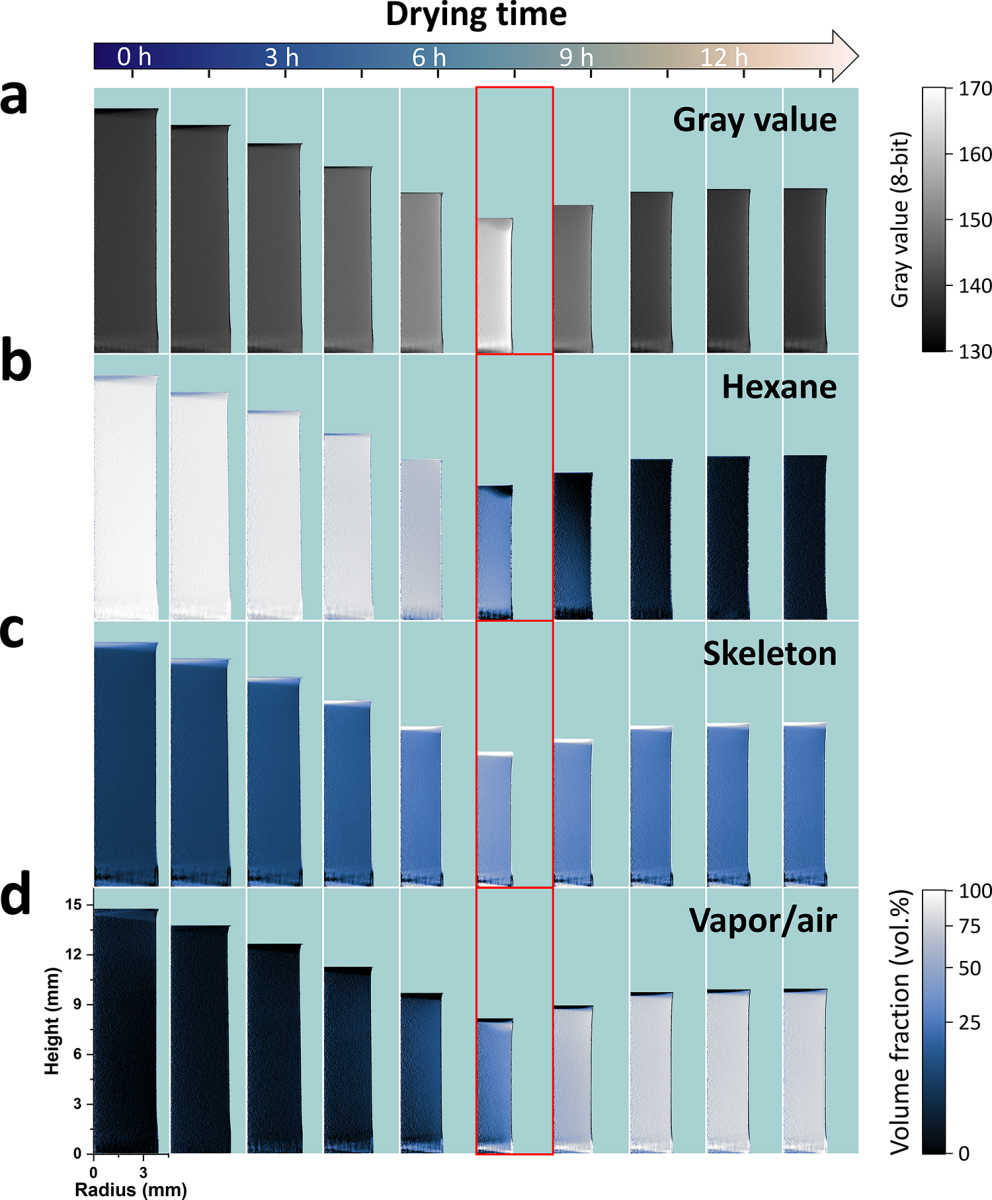
GHR and volume fraction maps of sample M4 at 10 selected drying stages on top of a cyan background. (a) GHR maps with the corresponding gray value scale on the right. The brightness and contrast in the images of the GHR maps are adjusted to improve visualization. (b) HEXHR maps. (c) SKELHR maps. (d) AIRHR maps. The color scale of the volume fraction maps is shown at the bottom right of the figure. The volume fraction maps are normalized between 0 and 100%. The images of the volume fraction maps are encoded with a gamma value of 0.5 to improve the visualization. The time scale is illustrated with an arrow on top of the figure, and the time gap between the maps in a given panel is 1.56 ± 0.05 h. The length scale of all maps is indicated in the first map of panel (d). The maps corresponding to the maximum shrinkage are outlined in red. Each map consists of 410 × 1455 noninterpolated data points.

**Figure 4:**
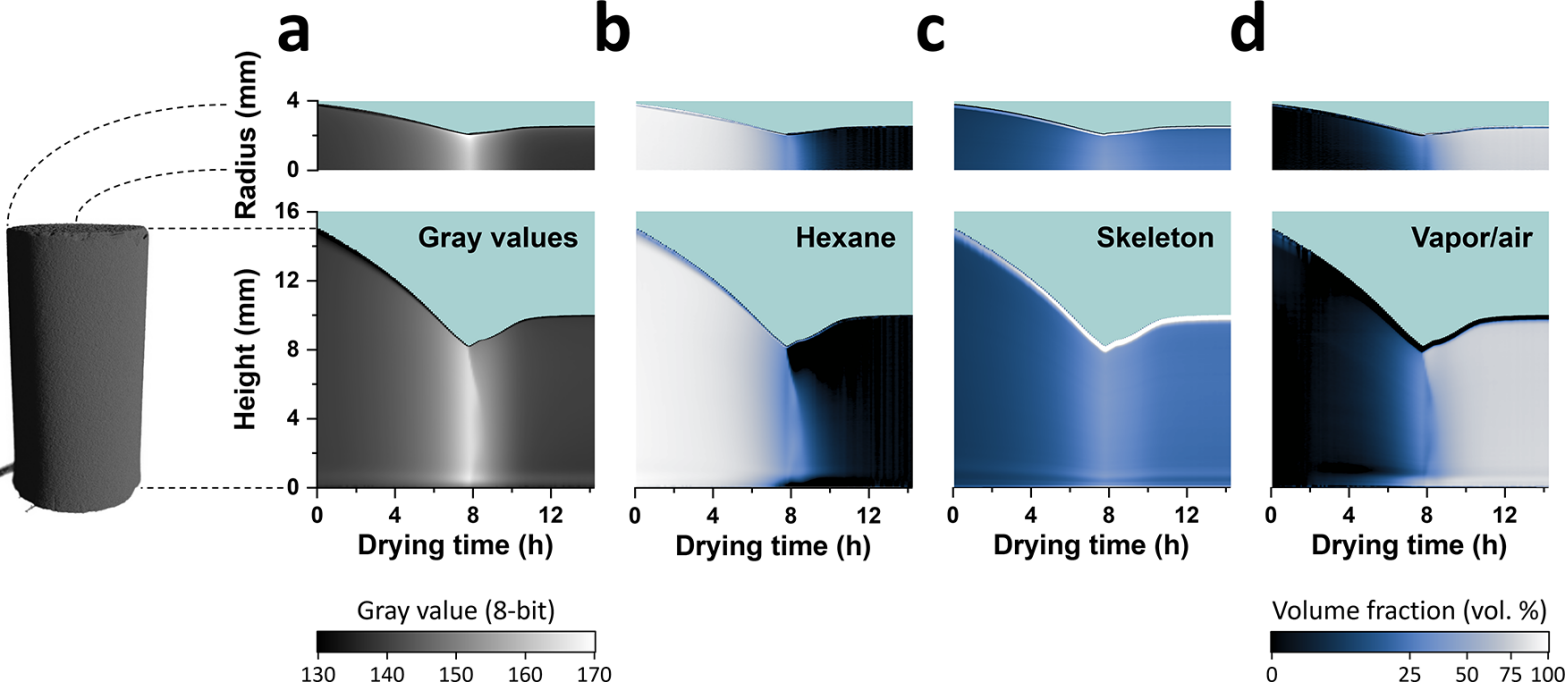
Radial and vertical maps of the gray values and volume fraction of sample M4 on top of a cyan background. The 3D image on the left of the figure depicts the segmented volume of M4 at the beginning of drying, and the dashed lines illustrate the radial and vertical axes of the cylinder against which the radial and vertical maps are shown. (a) GR and GH maps. The gray value scale is shown at the bottom of panel (a). The brightness and contrast in the images of the GR and GH maps are adjusted to improve visualization. (b) HEXR and HEXH maps. (c) SKELR and SKELH maps. (d) AIRR and AIRH maps. The images of the volume fraction maps are encoded with a gamma value of 0.5 to improve visualization. The time axis is shown in each vertical map, and the length scale is shown in the radial and vertical maps of panel (a). The radial maps consist of 1460 × 410 data points and the vertical maps consist of 1460 × 1455 points. In all maps, the horizontal time resolution is interpolated from 141 time stamps onto 1460 points.

This section reports and discusses the spatial distribution and evolution of the gel phase composition generated by μCT quantitative imaging. The azimuthal integration of the masked slices produced well-defined GHR maps shown in Figure 3a for sample M4 at different drying stages (see Video S1 for an animation of the 141 frames). The drying shrinkage can be seen with the decrease in height and maximum radius of the gel up to the maximum shrinkage at 7.6 h, together with an increase of the average gray values. The maximum shrinkage was followed by the re-expansion of the gel (spring-back effect) and by a decrease in the gray values. Silica gels dried in the fume hood under similar conditions remained transparent until the maximum shrinkage and only turned opaque upon re-expansion. The hexane, skeleton, and vapor/air volume fractions along the height and radius of sample M4 are shown in Figure 3b–d and in Video S1. At the start of drying, the hexane and skeleton phases were uniformly distributed in the gel at an average volume fraction of 94 and 6 vol %, respectively, suggesting a homogeneous gelation process. The fraction of vapor/air was 0 vol % as it was set in the drying model. Up to the maximum shrinkage, the hexane content decreased while the skeleton and vapor/air contents increased, each phase being still relatively homogeneously distributed across the entire gel’s volume. The emergence of the vapor/air phase in the gel was not clearly depicted in the AIRHR maps due to noise in the data, but Figure 3d shows a non-negligible amount of vapor/air before the maximum shrinkage, as already reported in ref (10). Near the end of drying, the skeleton and vapor/air spatial distributions were homogeneous, and after 14 h of drying, the gel was composed of about 22 and 78 vol % of skeleton and vapor/air, respectively. Heterogeneities in the repartition of hexane and vapor/air appeared at the maximum shrinkage where the hexane volume fraction abruptly dropped to ca. 0 vol % at the top of the gel (Figure 3b, Video S1). However, the fact that each map corresponds to a single drying stage and the presence of noise in the maps limited the analysis. The spatial and temporal distribution of the hexane and vapor/air phases was thus analyzed based on the vertical and radial volume fraction maps derived by modeling the GH and GR maps, which had the advantage of depicting the gel state at all drying stages along the two main axes of the cylindrical samples. The results are shown in Figure 4.

**Figure 5:**
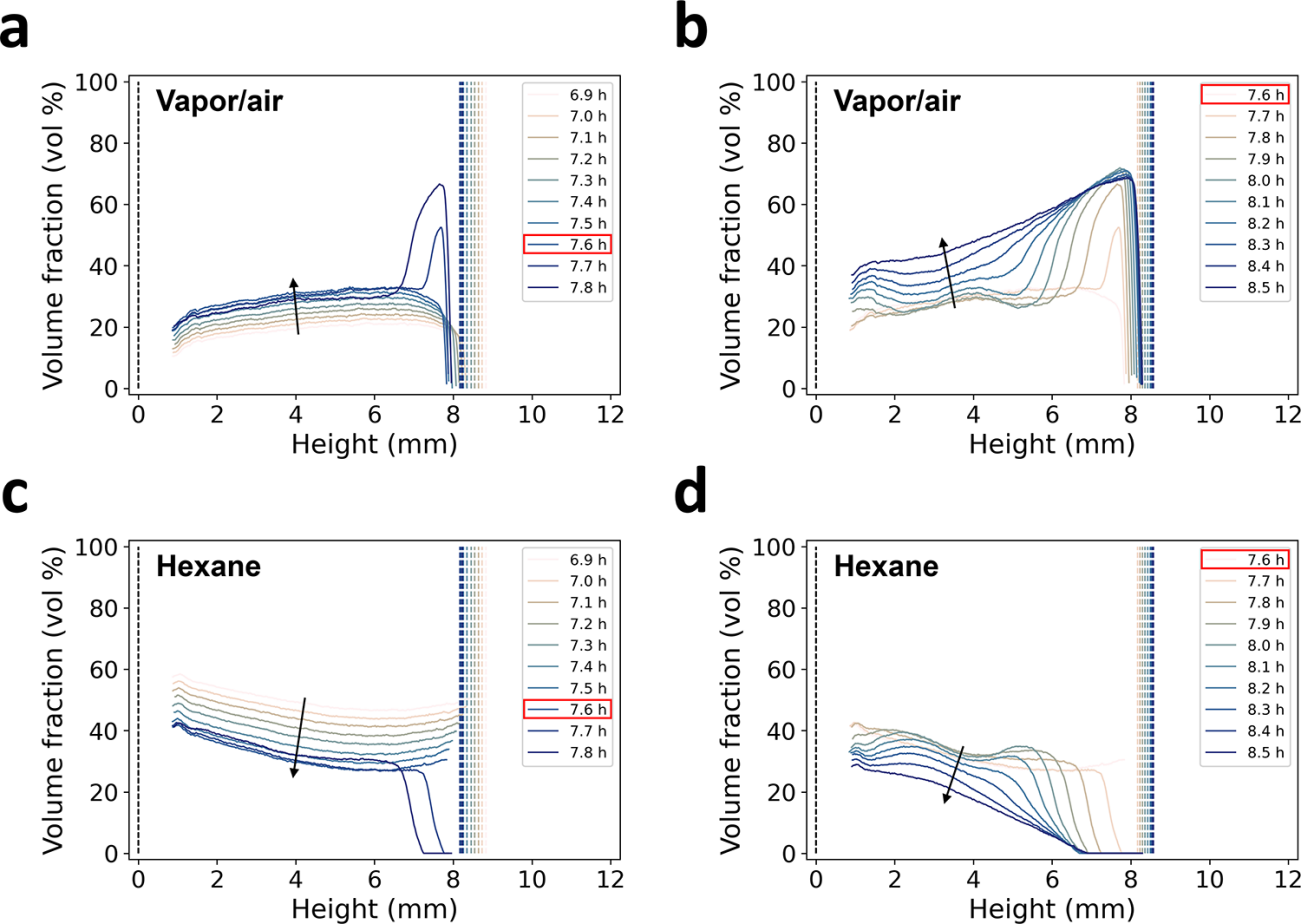
Volume fraction profiles of hexane and vapor/air along the height of sample M4 at selected time stamps. Vapor/air profiles were between 6.9 and 7.8 h (a) and between 7.6 and 8.5 h (b). Hexane profiles between 6.9 and 7.8 h (c) and between 7.6 and 8.5 h (d). The profiles in panels (a) and (c) correspond to the μCT scans before the maximum shrinkage and shortly after, while the profiles in panels (b) and (d) correspond to the μCT scans at the maximum shrinkage and after. The dashed lines correspond to the bottom (*h* = 0 mm) and the top of the sample. The time of maximum shrinkage is highlighted in red in the legends. The spacing between the profiles in each panel corresponds to a single μCT scan. The black arrows depict the drying time. The profiles were extracted from the AIRH and HEXH maps by excluding the values affected by the artifacts at the edges for better visualization.

The noise in the gray values of the vertical and radial maps in Figure 4a was significantly lower compared to the corresponding GHR maps, resulting in smoother volume fraction maps, which permitted a more accurate evaluation. Slight variations in the vertical distribution of hexane were observed before the maximum shrinkage, with a higher concentration of hexane at the bottom of the gel than at the top (Figure 4b). The top of the gel was composed of relatively more vapor/air than at the bottom before the maximum shrinkage (Figure 4d), whereas the skeleton spatial distribution remained static throughout the entire drying process, as set in the drying model (Figure 4c). Upon spring-back, significant variations in the repartition of hexane and vapor/air appeared along the height of the gel. The top region of the gel got depleted in hexane first, leading to an increase in the vapor/air volume fraction in the same region (Figure 4b,d). Hexane and vapor/air vertical profiles were extracted from the HEXH and AIRH maps to quantify the variations before and after maximum shrinkage (Figure 5). At the maximum shrinkage (7.6 h), the vapor/air content abruptly increased from 30 to 52 vol % near the top of the gel within 6 min (Figure 5a) and was followed by a wave-like drying front traveling downward in the sample as the gel started re-expanding (Figure 5b). At 7.9 h, the vapor/air content rose at the bottom of the gel which corresponded to another drying front traveling upward. At 8.5 h, those heterogeneities along the gel height stabilized, although a gradient of vapor/air was still present from the top to the bottom of the gel (Figure 5b). Similar but opposite features were noted in the hexane vertical profiles (Figure 5c,d). It was worth noting that at 8.5 h, there was still a significant fraction of hexane remaining with up to 29 vol % in the bottom regions of the gel (Figure 5c,d). At ca. 10 h of drying, the re-expansion of the gel slowed down and the variations in the hexane and vapor/air content along the gel height dissipated with an average hexane volume fraction close to zero and an average vapor/air volume fraction at 73 vol %. Between 10 and 14 h, the volume fraction of vapor/air slightly increased as the gel re-expanded to reach a final value of 78 vol % (Figure 4d).

**Figure 6:**
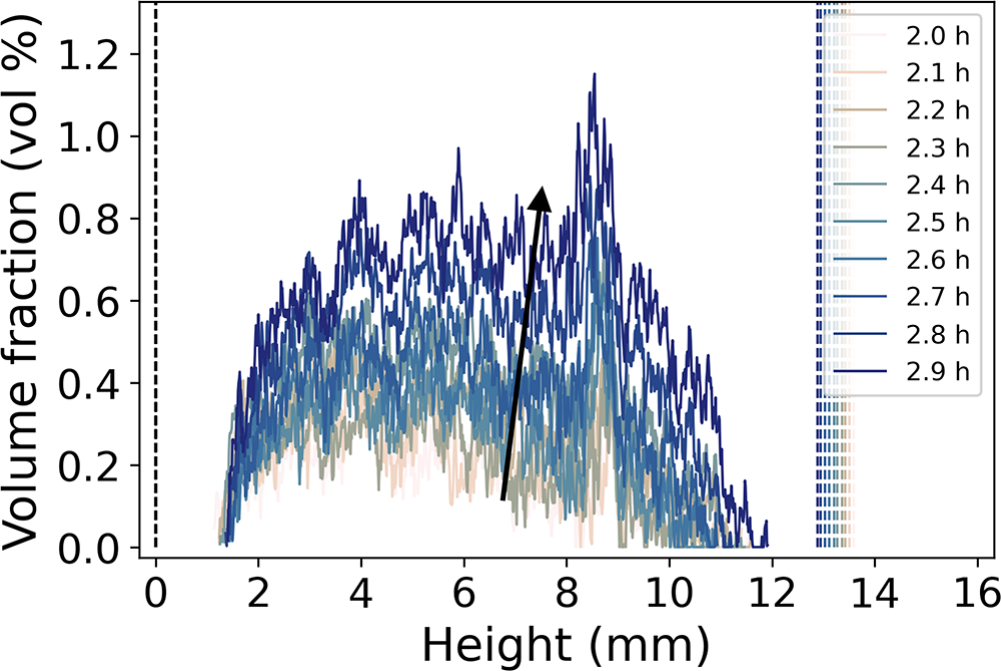
Vertical vapor/air profiles extracted from the AIRH maps of sample M4 between 2.0 and 2.9 h. The dashed lines correspond to the bottom (*h* = 0 mm) and the top of the sample. The spacing between each profile corresponds to a single μCT scan. The black arrow depicts the drying time.

The emergence of the vapor/air phase in the gels was referred to as the “cavitation onset” and was evaluated from the AIRH maps by extracting vertical volume fraction profiles near the start of drying (Figure 6). The vertical profiles indicated a nonzero amount of vapor/air from 2.0 h of drying in sample M4, with a higher concentration in the middle of the gel. Similar conclusions were drawn from the analysis of samples M1, M2, and M5. The only notable difference was a shift in the timing and duration of specific events such as the spring-back effect and the emergence of the vapor/air phase (especially in sample M1) due to slightly different starting volumes of the gels and possibly drying conditions (Figures S9–S18). As an attempt to quantify the cavitation onset, the AIRH maps (Figures 4d and S12d–S14d) were integrated over the height of the gel, giving an average vapor/air volume fraction. The cavitation onset was then defined as the drying time at which the average vapor/air volume fraction rose above an arbitrary volume fraction of 1 vol %. The time of cavitation onset for samples M1, M2, M4, and M5 was 2.93, 3.50, 3.64, and 3.43 h, respectively. Additionally, the pore volume shrinkage was calculated at the cavitation onset. It was defined as ν_*p*, *k*_cav__ = *V*_*p*, *k*_cav__/*V*_*p*,0_ where *V*_*p*, *k*_cav__, and *V*_*p*,0_ are the pore volume at the cavitation onset and the start of drying, respectively. The pore volume shrinkage was 64.1, 55.3, 52.7, and 51.8 vol % for samples M1, M2, M4, and M5, respectively. The time and pore volume shrinkage of sample M1 seemed to deviate compared to those of the three other samples. The global quantitative imaging analysis on that sample also showed deviations compared to the other samples.^10^ This sample put apart, the proximity of the results between the three other samples suggested that the cavitation onset was related to a particular state of the gel. Figures 5a and 6 showed a steady growth of the fraction of the vapor/air phase in the gel from the cavitation onset until the maximum shrinkage throughout drying, which was also observed in the other samples. Table 1 summarizes the properties of the gels upon the cavitation onset.

**Table 1:** Properties of All Samples at the Cavitation Onset^a^

sample	*t*_cav_ (h)	*t*_cav_/*t*_MS_	pore volume shrinkage at *t*_cav_ (vol %)	volume shrinkage at *t*_cav_ (vol %)
M1	2.93	0.360	64.1	66.5
M2	3.50	0.436	55.3	58.8
M4	3.64	0.471	52.7	55.8
M5	3.43	0.482	51.8	54.9

a *t*_cav_ and *t*_MS_ stand for the time of cavitation onset and the time of maximum shrinkage, respectively. The volume shrinkage is the ratio of the gel volume to the gel initial volume at the start of drying.

**Figure 7:**
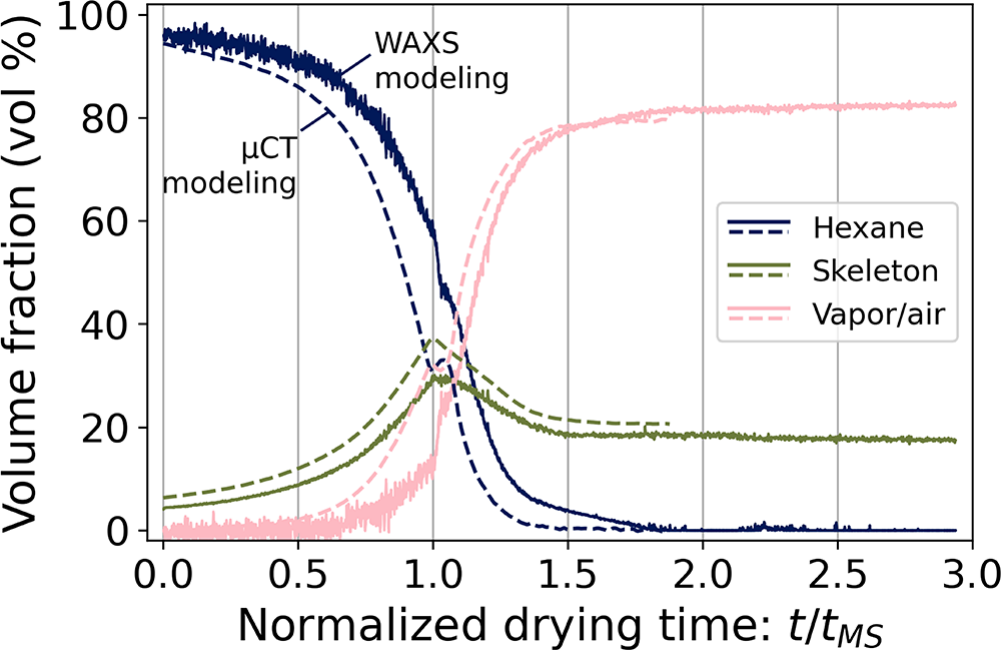
Average phase composition of gel M6 computed by WAXS modeling (full lines) along with the phase composition computed by μCT modeling, averaged over gels M1, M2, M4 and M5 (dashed lines) at the same location in the gel. The time scale is normalized by the time of maximum shrinkage *t*_MS_, which was 4.3 h for the WAXS modeling and ca. 7.5 h for the μCT modeling.

To corroborate the observations made from the μCT measurements, the phase composition derived by modeling of the WAXS data was compared with the results from μCT modeling at a representative location in the gel. Although WAXS did not allow spatial resolution of the vapor-air phase, it could detect the emergence of a vapor-air phase prior to the maximum shrinkage. Figure 7 shows the hexane, skeleton, and vapor/air volume fraction profiles computed from both methods against a normalized time scale. The evolution of the scattering profiles in the 3–30 nm^–1^ region and examples of the data fit can be found in Figures S19 and S20, respectively. The volume fraction profiles corresponded to the composition of the gels 4 mm above their bottom (where the X-ray beam probed the sample during the in operando X-ray scattering experiment). The profiles generated by these two methods were relatively consistent. The WAXS modeling results suggested a content of 14 vol % of vapor/air in the gel before the maximum shrinkage, supporting the μCT results. The initial and final compositions of the gels were similar between both methods with variations of ca. 2 vol %. In the probed location, the fraction of vapor/air raised above 1 vol % at *t*/*t*_MS_ ≈ 0.3 from the μCT measurement, and at *t*/*t*_MS_ ≈ 0.63 in the WAXS measurements (Figure 7). This cavitation onset in the gels dried in the μCT setup was lower than the values reported in Table 1 because it only accounted for the gas volume detected at a specific height in the gels. The differences observed between μCT and WAXS results during drying could be due to the different environments in the two experiments. Sample M6 dried faster in the scattering setup and reached the maximum shrinkage after ca. 4.3 h of drying versus ca. 7.5 h for the samples dried in the μCT setup. This could suggest that the gel composition and the cavitation onset have a nonlinear dependency on the drying rate. Additionally, the lower skeleton volume fraction from WAXS modeling compared to μCT modeling could be related to the assumptions on the chemical composition and density of the silica skeleton set in the WAXS drying model.

### Evaporation Mechanisms

We interpreted the spatial and temporal phase composition of the gels in terms of evaporation mechanisms, with an emphasis on cavitation. Based on the distribution and evolution of the hexane and vapor/air phases, we propose that the evaporative drying of the silica gels prepared in this study proceeded in three distinct stages:

(1) Evaporation by drying shrinkage from 0 to ca. 3.5 h.
(2) Evaporation by a combination of cavitation and drying shrinkage from ca. 3.5 to 7.5 h (maximum shrinkage).
(3) Evaporation by meniscus recession from ca. 7.5 h until complete evaporation of the remaining hexane.

During stage (1), the hexane content decreased homogeneously without any vapor/air inclusions (Figure 4d) and the volume of hexane evaporated matched the volume shrinkage of the gel, which was consistent with the drying shrinkage model.^15,20^ During stage (2), the drying shrinkage proceeded until the maximum shrinkage was reached in parallel with the growth of a vapor/air phase in the gels. Both the μCT and WAXS results showed that vapor/air appeared ahead of the maximum shrinkage and increased steadily, and μCT volume fraction maps revealed a relatively homogeneous distribution of vapor/air across the whole gel’s volume (Figures 3d, 5a, and 6), which suggested it was created by cavitation of hexane. In stage (3), the gel sprung back and a drying front was observed growing vertically through the sample (mostly downward). This stage was associated with the recession of the hexane-vapor interface into the pores and corresponded exactly to the re-expansion of the gels. Gels dried in the fume hood turned opaque upon re-expansion and gradually shifted toward a bluish color under a dark background, the latter being caused by Rayleigh scattering.^61^ The time resolution of the μCT experiments did not allow us to distinguish potential adiabatic burst events. Presumably, no additional cavities were created ahead of the drying front in stage (3) as the vapor/air volume fraction did not increase at those locations (Figure 5a). The gradient in the hexane and vapor/air volume fraction along the height of the gel during the re-expansion confirmed the heterogeneous nature of the spring-back effect which was attributed to the design of the drying chamber used in this study and in ref (10).

The hypothesis of cavitation was indirectly supported by two experimental observations. First, the vapor-air phase grew uniformly without any visible drying front. This was inconsistent with an evaporation mechanism by recession of the meniscus, which would proceed by the ingress of the liquid–vapor interface into the pores, forming a drying front. It also seemed unfeasible that evaporation by drying shrinkage and meniscus recession occurred simultaneously in the gels, given that the recession of the meniscus would result in local relaxation of the capillary stress, whereas the drying shrinkage kept proceeding steadily throughout stage (2). The absence of a drying front supports evaporation by cavitation although it must be noted that this feature might also be associated with other mechanisms, such as fractal-like penetration of the vapor phase.^62,63^ Second, the gels turned opaque only upon re-expansion and not at the cavitation onset. The change of transparency during stage (3) arguably corresponded to the apparition of pores filled with vapor/air that showed a characteristic size large enough to scatter visible light (400–700 nm). As such a change was not observed during stage (2), it suggested the absence of a continuous medium of vapor/air in the gels larger than 400 nm. This observation would be consistent with the growth of vapor/air within the gels by cavitation with cavities smaller than the wavelength of visible light. Nevertheless, it may be possible for gels to turn opaque upon cavitation.^40−42^ Those elements suggested that the vapor/air phase in the gel prior to the maximum shrinkage was not caused by meniscus recession but by cavitation of the solvent. Additionally, the cavitation onset coincided with a pore volume shrinkage of 51–64 vol % (Table 1). Because cavitation in porous materials depends on the porous media stiffness,^23,25^ which is closely related to the pore volume, the pore volume shrinkage is a critical parameter for the emergence of cavitation in the gels. As vapor/air bubbles appeared at the start of stage (2), they grew steadily and uniformly as shown by the vapor/air vertical distribution (Figures 4d, 5a, and 6). This growth was consistent with the phenomenology of evaporation by cavitation and drying shrinkage. The first cavities were presumably created in large pores ahead of the outer surface of the gel, where the liquid confinement by the solid matrix was the smallest.^23^ At this stage, there was no deceleration of the volume shrinkage upon the emergence of vapor/air in the gel.^10^ As the drying shrinkage proceeded, the tension in the liquid rose, which enabled the nucleation of additional cavities in smaller pores and possibly the growth of already-created bubbles. Shortly before maximum shrinkage, the volume shrinkage decreased,^10^ which could indicate a stress release by cavitation.

To assess whether cavitation can actually occur in silica gels filled with hexane, the pressure and required pore size were estimated following the arguments of ref (64). The energy change associated with the creation of a spherical cavity of radius *R* is 4π*R*^3^*p*_l_/3 + 4π*R*^2^γ, with *p*_l_ < 0 the pressure in the liquid.^14,65^ A bubble with a critical radius larger than R* = −2γ/*p*_l_ will spontaneously grow, and the corresponding energy barrier is Δ*E* = 16πγ^3^/3*p*_l_^2^.^65^ Cavitation can occur only in pores larger than R*. According to the classical nucleation theory, the nucleation rate is^64^
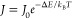
18where *J*_0_ is a prefactor in cm^–3^ s^–1^, *k*_B_ is the Boltzmann constant, and *T* is the temperature. Silica gels display a fractal structure^66^ with a wide size distribution of pores (1–100 nm).^3^ In silica gels, cavitation will occur in sufficiently large pores if the pressure generated at the meniscus (in smaller pores within the network) is large enough to generate a reasonable nucleation rate, as shown in eq 18. At the meniscus, the pressure in the liquid is given by eq 1: *p*_*l*_ = *p*_0_–2γ/*r* assuming that hexane fully wets the gel (θ = 0), where *r* is the smallest pore radius blocking the recession of the meniscus in the gel. In nanometric pores, |*p*_l_| ≫ |*p*_0_| and the vapor pressure can be neglected.^39^ The energy barrier for the nucleation of a bubble can be rewritten as a function of the smallest pore radius: Δ*E* = 4πγ*r*^2^/3. Ref (64) provides an estimate for the prefactor *J*_0_ for cavitation conditions: , with ρ being the liquid density, *N*_A_ being Avogadro’s constant, and *M*_w_ being the molar mass of the liquid.

**Figure 8:**
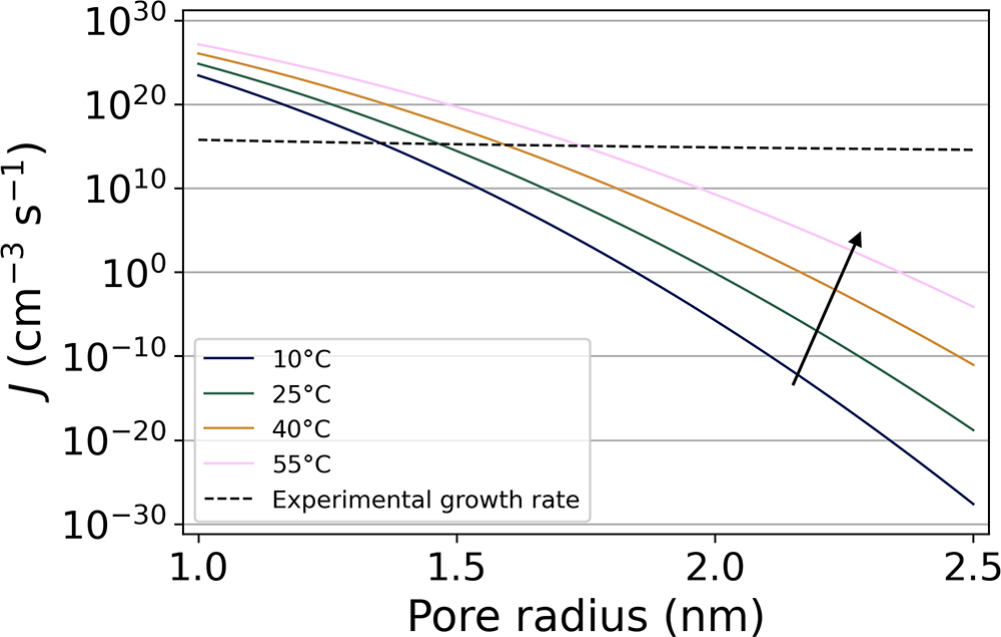
Calculated rate (full lines) of the formation of hexane bubbles *J* as a function of the smallest pore radius *r* blocking the recession of the meniscus at different liquid temperatures. Vapor/air growth rate (dashed line) was calculated from the μCT experiments on sample M4, assuming the creation of spherical bubbles of radius *r*. The black arrow depicts the temperature increase.

The nucleation rate of hexane in silica gels was then numerically estimated as a function of the smallest pore radius in the gel using eq 18. The dependence of *n*-hexane surface tension on the temperature was taken into account using a modified van der Waals equation reported in ref (67). The results are shown in Figure 8. The nucleation rate showed an extreme dependence on the radius of the smallest pores. The liquid temperature also has a strong influence on the nucleation rate, promoting cavitation at higher temperatures as already reported elsewhere.^26,27,29^

To evaluate what would be a reasonable nucleation rate for cavitation in silica gels, the numerical estimates of *J* were compared with the experimental growth rate of vapor/air from the μCT analysis. The growth rate per unit volume and per unit time was calculated as *J*_exp_(*r*) = Δ*f̅*_air_/(Δ*t* · 4π*r*^3^/3), where Δ*f̅*_air_ is the difference of the average volume fraction of vapor/air in gel M4 taken between *t*_cav_ and *t*_MS_, with *t*_cav_ the time of cavitation onset and *t*_MS_ the time of maximum shrinkage, and Δ*t* = *t*_cav_–*t*_MS_ = 4.1 h. Δ*f̅*_air_/Δ*t* was a good approximation of the vapor/air growth rate (Figure S21). The profile *J*_exp_(*r*) corresponds to the creation of spherical vapor bubbles of radius *r* that neither grow nor collapse during the time lapse Δ*t*. The intersection between the profiles *J* from the CNT estimations and *J*_exp_ in Figure 8 gives an approximation of the nucleation rate that would correspond to the growth rate of the vapor/air phase in gel M4. At 25 °C (average temperature in the μCT instrument),^10^ a nucleation rate of about 2 · 10^15^ cm^–3^ s^–1^ was found for a pore radius blocking the meniscus recession of *r* = 1.47 nm. Under these conditions, the liquid pressure would be *p*_l_ ≈−24 MPa, which was in good agreement with the values found by optical measurements of hexane desorption in alumina membranes.^22^ These results supported that hexane cavitation can occur in silica gels, which have a small enough pore size for the liquid to reach the negative pressure required to form hexane bubbles at a reasonable rate. However, those results did not consider the dynamic of hexane bubbles over time. The spontaneous growth of already formed bubbles could notably produce the vapor/air volume in sample M4 at a much lower nucleation rate; thus, cavitation might also occur at larger pore radii. On the other hand, the hexane temperature during drying was likely lower than 25 °C due to the latent heat of vaporization, and this would reduce the nucleation rate at a fixed pore radius.

The timing and extent of cavitation could largely differ in gels resulting from different synthesis routes, depending on the porous network, stiffness of the solid matrix, and the nature of the solvent. Notably, in certain systems, cavitation may be absent. Cavitation in the silica gels synthesized in this study could have been facilitated by the presence of a thicker layer of silica on the outer surface of the gels, which may have formed during gelation in the molds. Such a shell with a smaller pore size would have a pore-blocking effect,^28^ generating a stronger capillary pressure during drying that enables nucleation of vapor/air bubbles ahead of the gel surface. Additionally, cavitation of solvents with a low surface tension (such as hexane) is facilitated since the free energy of cavitation bubbles is proportional to γ^3^ at a given liquid pressure.^14,64^ As shown by the CNT estimations, the temperature at which hexane evaporation occurs has a significant influence on the cavitation rate. Performing ambient-pressure drying at higher temperatures may therefore reduce the drying stress and possibly allow the production of larger monolithic aerogels with higher spring-back efficiencies, which may be investigated in the future. To our knowledge, the effect of the drying temperature on the spring-back efficiency and size of monolithic aerogels has not been investigated, though sometimes temperatures of 50 °C and more have been used. The surface chemistry of the silica network may also play a role: defects on the silica surface (e.g., remaining silanol groups due to incomplete silylation) can enable heterogeneous nucleation of vapor/air bubbles which require less energy than homogeneous nucleation,^31,68^ meaning that cavitation can occur at a reduced liquid pressure. However, recent investigations in porous silicon suggest that desorption exclusively takes place through homogeneous cavitation in that system.^22,26^ This is attributed to the complete wetting of silicon and most surfaces by hexane, liquid nitrogen, and liquid helium, owing to their exceptionally low surface tension. Cavitation in silica gels could also be detected by complementary measurements and analyses, such as acoustic measurements^69−71^ and Small-Angle X-ray Scattering (SAXS),^72^ which were not conducted in this work.

### Reliability and Limitations

This section discusses the reliability of the μCT data reduction procedures and modeling and lists the artifacts generated experimentally and computationally. The GR maps of all samples indicated a heterogeneous distribution of the gray values along the gel radius at all drying stages (Figures 4a and S12a–S14a). This feature was also visible in the GHR maps to a lesser extent (Figures 3a and S9a–S11a). Those variations were more pronounced near the maximum shrinkage. A spatial variability analysis revealed an exponential dependency of the gray values on the gel radius (SI5). The relative increase of the gray values along the radius was correlated to the gel diameter and attributed to beam hardening,^43^ although potential heterogeneities in the skeleton concentration along the gel radius could not be excluded. A spatial variability analysis on the gray values across the gel’s height showed no sign of beam hardening. A similar analysis of the distribution of the gray values along the azimuth of the cylinders revealed slight variations that were caused by reconstruction artifacts rather than being a physical feature of the samples (SI5). Darker lines can be seen on the top and bottom of the gel and its outer radius in the GHR maps (Figure 3a). These were caused by background inclusions in the masked images due to imperfect segmentation. Locally higher gray values were observed at a height of ca. 0.7 mm from the bottom of the gels (Figure 3a), which were reconstruction artifacts due to the proximity of the sample to the bottom of the PEEK drying chamber and the low number of CT projections used for reconstruction. The artifacts in the GHR maps were propagated to the GR and GH maps, where similar features were observed at the vertical and radial edges of the samples. Despite these artifacts, the overall kinetics of the gray values in the GHR, GR, and GH maps were consistent with the results of the global quantitative imaging analysis (SI6). This confirmed the reduction procedures could generate accurate representations of the shape-changing samples. The histograms of the GHR maps also showed a narrower distribution of the gray values compared to the histogram of the masked slices, as a result of the azimuthal integration (Figure S31). Lastly, the gray values in the raw reconstructed slices were affected by the anode heel effect,^47^ which was corrected by the method developed in ref (10).

The volume fraction maps were affected by the artifacts in the gray value maps. The abnormally high/low volume fraction of a given phase at the edges of the gel (Figures 3b–d and 4b–d) corresponded to the locations where the segmentation included the background in the masked slices, which was interpreted in the drying model as a change in the composition. The abrupt changes at the top, bottom, and radial edges of the gels were thus treated as artifacts. The radial variations of the gray values were also propagated to the volume fraction maps, which can be seen, for example, in the increasing vapor/air volume fraction with the gel radius in the AIRR map of sample M4 before the maximum shrinkage (Figure 4d). Those variations, being partially attributed to beam hardening, prevented making reliable interpretations of the radial distribution of hexane, skeleton, and vapor/air during drying. The vertical maps were the most reliable representations to quantify the phase composition of the gels as they appeared to be free of beam hardening artifacts. Nonetheless, the HEXHR, SKELHR, and AIRHR maps depicted qualitative aspects of the composition evolution during drying despite the artifacts in the radial direction. The kinetics of the hexane, skeleton, and vapor/air volumes were consistent with the results from the global quantitative imaging approach,^10^ besides some irregularities near the maximum shrinkage that were possibly related to the additional computational steps required in the presented method (SI6).

At the beginning of stage (3), as the drying front emerged on the top of the gel, an increase in the hexane concentration was observed in regions ahead of the drying front in all samples (Figures 5c and S16c–S18c). A similar but opposite feature was observed in the vapor/air phase with a decrease in the vapor/air volume fraction at those locations. This phenomenon can also be seen in Figure 7, where the hexane and vapor/air volume fractions were computed at a constant height in the gel. Most likely this phenomenon was related to an artifact arising from the assumption of a temporally static skeleton distribution throughout drying. This assumption seemed valid for most of the drying process, as the volume shrinkage appeared to be uniform along the height and radius of the gel (Figure 3), but may present limitations at the onset of stage (3) due to the heterogeneous nature of the spring-back effect in this study. The HEXHR maps were computed based on an artificial MHR map representative of the dry gel, which was rescaled toward a target scan. That artificial MHR map was stretched by bilinear interpolation to fit the target scan domain and corrected by a scalar scaling factor given by the volume ratio between the source scan and the target scan. The overall volume of the gel increased at the spring-back, which decreased the rescaling factor γ_*k*_*f*_ → *k*_ used to compute the local hexane volume fraction (see eq 7), resulting in an effective increase of the local hexane volume fraction that did not account for the heterogeneous re-expansion of the gel. This also resulted in an overall decrease of the vapor/air volume fraction ahead of the drying front, as it was calculated from eq 5. Nevertheless, it could not be excluded that part of the observed variations were a physical feature of the sample. The sudden relaxation of the liquid tension could notably destabilize the equilibrium of the vapor/air bubbles close to the drying front and possibly result in a collapse of the cavities. This would increase again the tension in the liquid, which may pull more hexane ahead from the drying front, resulting in an effective increase of the hexane volume fraction at the corresponding location. Including the local re-expansion of the gel in the bilinear interpolation routine would require generating a dynamic mesh depending on both the shape changes and local gray values, which was out of the scope of this study.

## Conclusions

This study addressed the evaporation mechanisms during the evaporative drying of silica gels by evaluating the spatial and temporal phase compositions modeled by μCT quantitative imaging. A noteworthy discovery was made, as the observed evolution of vapor/air content in the gels aligned with the concept of evaporation by cavitation initially theorized by Scherer and Smith in 1995. The presence of vapor and air in the gels before the maximum shrinkage was confirmed by WAXS modeling. The repartition of hexane and vapor/air in the gels was successfully computed by an in operando μCT workflow, which also demonstrated the potential of μCT quantitative imaging to generate local phase composition maps of the shape and composition of evolving materials. Based on these results, we proposed that the evaporative drying of silylated silica gels proceeded in three stages: (1) evaporation by drying shrinkage; (2) evaporation by drying shrinkage and by cavitation; (3) evaporation by the recession of the meniscus, challenging the common drying model associated with sol–gel processes. By using classical nucleation theory, we have also derived the nucleation rate and smallest pore radius required to create the vapor/air volume computed by μCT quantitative imaging, which supported that cavitation can occur in silica gels. The emergence of cavitation was correlated with a pore volume shrinkage of about 50 vol % that was attributed to the critical point where the silica matrix stiffened enough to enable the nucleation of cavities. Cavitation started as early as 3.4 h of drying time, whereas the maximum shrinkage occurred at 7.6 h of drying.

In general, this discovery highlights cavitation as a new potential mechanism for evaporation in silica gels and makes a valuable contribution to understanding drying processes in porous materials. Tailoring the evaporative drying process by cavitation could be advantageous in the production of monolithic aerogels as it would reduce the pressure gradients in the gels and alleviate the rise of the capillary pressure, which would in turn reduce the risk of cracks appearing during drying. This could be done by precise modulation of gel properties (specifically pore size and matrix stiffness) and optimization of drying conditions and may represent a promising route for substantial advancements in the fabrication of monolithic aerogels through ambient-pressure drying, a process currently constrained in its application. The study notably highlighted the potential of carrying out evaporative drying at temperatures higher than room temperature, which may promote cavitation. The characterization of cavitation in the drying process of silica gels could be expanded through the application of alternative nondestructive methodologies, including acoustic detection and SAXS.

## Data Availability

The raw data, processed data, python scripts and intermediate results are openly available and are archived on Edmond^73^https://doi.org/10.17617/3.OYI3T.

## Supporting Information Available

μCT data reduction procedure, derivation of the μCT drying model, bilinear interpolation algorithm, artifact in sample M3, determination of the gel diameter during in operando X-ray scattering measurements, volume fraction maps and profiles of samples M1, M2, and M5, time series and fitting of WAXS data, gradient of vapor/air average volume fraction, spatial variability analysis of μCT data, comparative analysis of μCT quantitative imaging approaches, histograms of masked slices and GHR maps (PDF)
Video of the distribution of the gray values and hexane, silica skeleton, and vapor/air volume fractions along the gel’s height and radius for sample M4 (MP4)
