# Supplementary material for: Solvent Cavitation during Ambient Pressure Drying of Silica Aerogels
Source: Langmuir. 2024 Jun 12;40(25):12925–38. doi: 10.1021/acs.langmuir.4c00497 (PMC11210208; doi:10.1021/acs.langmuir.4c00497)
Supplement: Supplementary file 1 — la4c00497_si_001.pdf [file la4c00497_si_001.pdf]

Supporting information

Solvent cavitation during Ambient Pressure Drying of Silica Aerogels

Julien Gonthier,[*,†] Ernesto Scoppola,[†] Tilman Rilling,[†] Aleksander Gurlo,[‡] Peter Fratzl,[†] Wolfgang Wagermaier*[*,†]

[†] Department of Biomaterials, Max Planck Institute of Colloids and Interfaces, 14476 Potsdam, Germany

[‡] Chair of Advanced Ceramic Materials, Institute of Materials Science and Technology, Faculty III Process Sciences, Technische Universität Berlin, 10623 Berlin, Germany

*Email: julien.gonthier@mpikg.mpg.de, wolfgang@wagermaier.mpikg.mpg.de

Table of contents

SI1: Data Reduction Procedure 3

SI2: Derivation of the μCT Drying Model 5

SI3: Bilinear Interpolation Procedure 11

Figure S5 15

SI4: Gel Diameter during X-ray scattering measurements 15

Figure S9 19

Figure S10 20

Figure S11 21

Figure S12 22

Figure S13 22

Figure S14 23

Figure S15 24

Figure S16 25

Figure S17 26

Figure S18 27

Figure S19 28

Figure S20 28

Figure S21 29

SI5: Spatial Variability Analysis 29

SI6: Comparative analysis of the Quantitative Imaging Approaches 36

Figure S30 42

References 42

SI1: Data Reduction Procedure

The in operando µCT measurements generated a 4D dataset as 3D reconstructed volumes over time for each sample. This section describes the three reduction procedures used to reduce the 4D dataset. Note that in the main text, the notation was simplified and the 4D dataset was introduced as $g_{i,p,q,k}$, describing it as the gray values already corrected for the anode heel effect. In practice, the anode heel effect correction was performed after a preliminary integration.

The 4D gray values were referred to as $\tilde{g}_{i,p,q,k}$, with $i, p, q, k \in \mathbb{N}$. The tilde stands for uncorrected, the index i depicts the vertical position in the sample in voxels, the indexes p and q depict the horizontal positions in voxels and the index k stands for the scan number (time). Figure S1 illustrates the three spatial axes and the time axis. Note that the index i was defined pointing downwards following the convention for digital pictures. The 4D dataset consisted of a series of images (the masked slices) indexed by i and k. As a reminder, the masked slices are 8-bit tif images that were generated upon segmentation of the reconstructed volume by replacing the value of the pixels outside of the sample by zero.

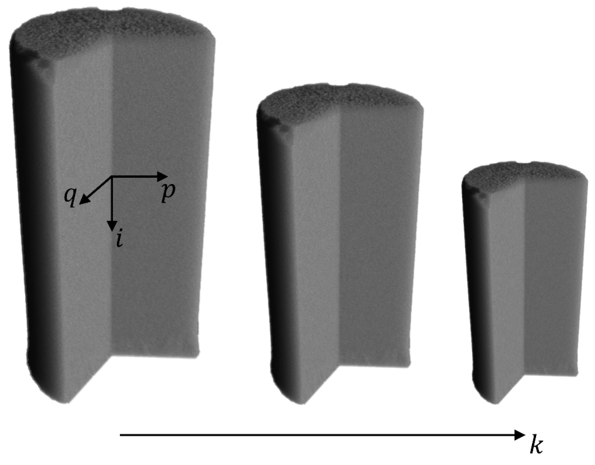

Figure S1. Sketch of three reconstructed volumes along with the three spatial axes where the indexes i, p, q are defined and the time axis where the index k is defined.

Azimuthal integration. The azimuthal integration generated the gray value maps showing the evolution of the gray values along the gel radius and height over time (GHR maps). The procedure was performed on the masked slices using the Python library DipLib [1]. The center of the sample cross-section in the image was calculated as the center of mass (first order moments) of the image with the function *diplib.CenterOfMass().* The azimuthal integration was then computed with the function *diplib.RadialMean()* using a bin size of one pixel. Integrated gray values at a radial distance from the center larger than 1.05 times the average radius of the gel at a given scan number were set at zero. This was done to limit the propagation of the imperfect segmentation at the bottom of the gel. Additionally, if the area of the sample in the image was less than half of the mean cross-section area of the gel, the integrated gray value of the masked slice was set to zero. This criterion allowed to exclude the noise at the top of the reconstructed

volume due to imperfect segmentation. The mean cross-section area was calculated from the average diameter of the gel from our recent work [2].

The profiles created by azimuthal integration of all masked slices at a given scan number k were then combined into a single map, referred to as $\tilde{g}_{i,j,k}$, where $j \in \mathbb{N}$ represents the radial distance to the center of the cylinder in pixels. The gray values were then corrected for the anode heel effect with:

$$g_{i,j,k} = \tilde{g}_{i,j,k} + (i_p - i) \cdot m^*(\tilde{g}_{i,j,k}), \quad (1)$$

where $g_{i,j,k}$ are the maps corrected for the anode heel effect, i_p is the pivot slice number and $m^*(g)$ is an exponential decay function. The parameters used in eq. (1) were the same as in ref. [2] (see Supporting Information, section SI3). The GHR maps were saved as npy files for further processing and as 8-bit tif images for illustration purposes. Figure S2 illustrates the azimuthal integration procedure.

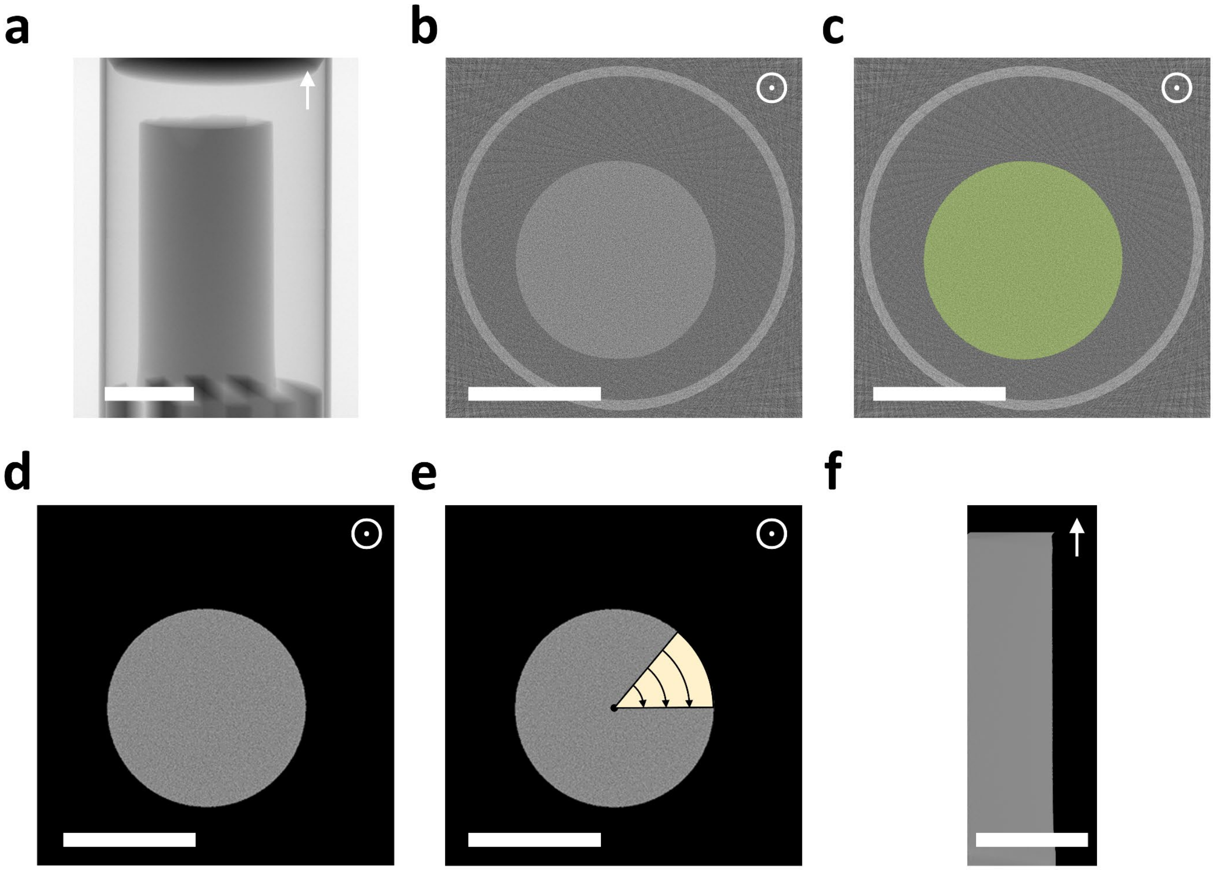

Figure S2. Illustration of the reduction procedure by azimuthal integration on sample M2 at the start of drying. (a) µCT projection at the start of drying. (b) Reconstructed slice at $i = 300$. (c) Same slice overlaid with the ROI from the automated segmentation. (d) Corresponding masked slice. (e) Sketch of the azimuthal integration on the masked slice. (f) Final GHR map created by combining the radial gray value profiles generated by the azimuthal integration at each index i. The scale bar in each panel is 5 mm. The gray values are in 8-bit. The arrow in each panel stands for the vertical direction of the gel.

Azimuthal and vertical integration. The azimuthal and vertical integration generated a single radial gray value map (GR map) for a given sample, showing the evolution of the gray values along the gel radius over time. The GHR maps generated by azimuthal integration were further integrated along the index i (along the height of the gel) to compute the GR map:

$$g_{j,k} = \frac{1}{H_{\Omega_{j,k}}} \sum_{i \in \Omega_{j,k}} g_{i,j,k}, \tag{2}$$

where $g_{j,k}$ is the GR map, $H_{\Omega_{j,k}}$ is the height in pixel of the domain $\Omega_{j,k}$ which defines the range of i indexes belonging to the sample. Note that the domains $\Omega_{j,k}$ were cropped, so that the sum in eq. (2) was effectively done by excluding a top and bottom band of 70 pixels. This was done as an attempt to get a more representative evolution of the gray values along the gel radius over time. Moreover, since the GHR maps were already corrected for the anode heel effect, no additional correction procedure was required.

Slice integration. The slice integration generated a single vertical gray value map (GH map) for a given sample, showing the evolution of the gray values along the gel height over time. In practice, this reduction procedure was carried out directly in the software Dragonfly [3] using the "slice analysis" plugin. The masked slices were integrated along the indexes p and q as follows:

$$\tilde{g}_{i,k} = \frac{1}{A_{\Omega_{i,k}}} \sum_{p,q \in \Omega_{i,k}} \tilde{g}_{i,p,q,k}, \tag{3}$$

where $\tilde{g}_{i,k}$ is the uncorrected GH map, $A_{\Omega_{i,k}}$ is the area in the masked slice within the domain $\Omega_{i,k}$ defining the range of p and q indexes belonging to the sample. The map $\tilde{g}_{i,k}$ was then corrected for the anode heel effect with a similar expression as in eq. (1).

SI2: Derivation of the μCT Drying Model

Here the μCT drying model is derived by developing the equations step-by-step, leading to the final expression for the spatial and temporal volume fraction maps of the three phases composing the gel. As mentioned in the main text, the reconstructed attenuation coefficient (RAC) of each pixel belonging to the sample in the MHR maps is given by:

$$\mu_{i,j,k} = \mu_{\mathrm{hex}} f_{\mathrm{hex},i,j,k} + \mu_{\mathrm{skel}} f_{\mathrm{skel},i,j,k}, \tag{4}$$

where μ_{hex} and μ_{skel} is the RAC of the hexane and skeleton phases, respectively and $f_{\mathrm{hex},i,j,k}$ and $f_{\mathrm{skel},i,j,k}$ are the volume fraction of hexane and skeleton in each pixel. $\mu_{\mathrm{hex}} = 0.155$ from separate measurements on hexane. Volume conservation within each pixel of the MHR maps reads:

$$f_{\mathrm{hex},i,j,k} + f_{\mathrm{skel},i,j,k} + f_{\mathrm{air},i,j,k} = 1. \tag{5}$$

Eqs. (4) and (5) were the main equations of the drying model. To calculate the volume fraction maps of each phase, some assumptions were made on the evaporative drying process. First, the total skeleton volume was assumed constant throughout drying: $V_{\mathrm{skel},k} = \mathrm{constant}$, which can be expressed locally as:

$$\sum_{(i,j)\in\Omega_k} f_{\mathrm{skel},i,j,k} V_{\mathrm{voxel}} = \mathrm{constant}. \tag{6}$$

Second, the content of hexane after a certain time was assumed to be zero everywhere in the gel, leading to: $f_{\mathrm{hex},i,j,k\geq k_d} = 0$, with k_d a threshold scan number upon which the gel was assumed dry. Lastly, the initial content of vapor/air was assumed to be zero everywhere in the gel, leading to: $f_{\mathrm{air},i,j,k_1\leq k\leq k_2} = 0$ where k_1 and k_2 are threshold scan numbers. Scan k_1 defined the time from which the instabilities in the X-ray tube disappeared and scan k_2 stood for the time where vapor/air started to enter the gel. k_d, k_1 and k_2 were calculated using the global quantitative imaging approach reported in ref. [2]. Additionally, a variation of the skeleton volume conservation equation can be derived by taking the sum over the domain Ω_k of eq. (4):

$$\sum_{(i,j)\in\Omega_k} \mu_{i,j,k} - \mu_{\mathrm{hex}} f_{\mathrm{hex},i,j,k} - \mu_{\mathrm{skel}} f_{\mathrm{skel},i,j,k} = 0, \tag{7}$$

$$\Leftrightarrow \sum_{(i,j)\in\Omega_k} \mu_{i,j,k} - \mu_{\mathrm{hex}} f_{\mathrm{hex},i,j,k} = \mathrm{constant}. \tag{8}$$

Where eq. (8) was derived using the conservation of the skeleton volume. Taking eq. (5) for $k_1 \leq k \leq k_2$ gives:

$$f_{\mathrm{hex},i,j,k} = 1 - f_{\mathrm{skel},i,j,k}, \quad k_1 \leq k \leq k_2. \tag{9}$$

By replacing $f_{\mathrm{hex},i,j,k}$ in eq. (8) by its expression in eq. (9), we get:

$$\sum_{(i,j)\in\Omega_k} \mu_{i,j,k} - \mu_{\mathrm{hex}}\left(1 - f_{\mathrm{skel},i,j,k}\right) = \mathrm{constant}, \quad k_1 \leq k \leq k_2, \tag{10}$$

$$\Leftrightarrow \sum_{(i,j)\in\Omega_k} \mu_{i,j,k} - \mu_{\mathrm{hex}} = \mathrm{constant}, \quad k_1 \leq k \leq k_2. \tag{11}$$

Eq. (11) was not an additional equation per se, but will be used later on.

Hexane volume fraction. We first derive the expressions used to calculate the hexane volume fraction maps throughout drying. To improve the statistics, the calculations were done on a MHR map representative of the dry gel for $k \geq k_d$. The hexane volume fraction $f_{\mathrm{hex},i,j,k}$ is calculated by finding an expression for $\mu_{\mathrm{skel}} f_{\mathrm{skel},i,j,k}$ in eq. (4). For $k \geq k_d$, eq. (4) becomes:

$$\mu_{i,j,k} = \mu_{\mathrm{skel}} f_{\mathrm{skel},i,j,k}, \quad k \geq k_d, \tag{12}$$

because there was no more hexane in the gel at $k \geq k_d$. By combining the MHR maps $\mu_{i,j,k}$ over $k \geq k_d$, an artificial MHR map representative of the dry gel could be generated. However, the volume of the gel still changed at $k \geq k_d$, so did the domains Ω_k, preventing to average the MHR maps directly. We write formally:

$$\Omega_{k_a} \neq \Omega_{k_b}, \ \ k_a \neq k_b. \tag{13}$$

This was solved by resizing one map onto another using bilinear interpolation so that the domain of the maps matched. The MHR map to be resized was referred to as the source and the map over which it was resized was referred to as the target. In this case, the target was unique and was set as the MHR map of the last scan ($k = k_f$), while the source scans were multiple ($k_d \leq k < k_f$). Note that the target scan could have been any scan within $k \geq k_d$. We define:

$$\mu^*_{i,j,k \to k_f} = \mu^F_{i,j,k \to k_f} \cdot \gamma, \ \ k_d \leq k < k_f, \tag{14}$$

where $\mu^*_{i,j,k \to k_f}$ is the rescaled MHR map of scan k towards scan k_f, $\mu^F_{i,j,k \to k_f}$ is the interpolated map defined over the domain Ω_{k_f} and γ is a correction factor. Formally, we also define $F_{k \to k_f}$ as the bilinear interpolation function from a source k towards a target k_f:

$$\mu^F_{i,j,k \to k_f} = F_{k \to k_f}\left(\mu_{i,j,k}\right). \tag{15}$$

The algorithm for the bilinear interpolation function is described in SI3. A series of rescaled maps $\mu^*_{i,j,k \to k_f}$ for $k_d \leq k < k_f$ was thus obtained, each map being defined on the same domain Ω_{k_f}. To derive the scaling factor in eq. (14), let's consider a quantity that stays constant throughout drying. We recall the conservation of the skeleton volume:

$$\sum_{(i,j) \in \Omega_k} f_{\text{skel},i,j,k} V_{\text{voxel}} = \text{constant}. \tag{6}$$

By substituting $f_{\text{skel},i,j,k}$ in eq. (6) by its expression from eq. (12), we get:

$$\sum_{(i,j) \in \Omega_k} \frac{\mu_{i,j,k}}{\mu_{\text{skel}}} V_{\text{voxel}} = \text{constant}, \ \ k \geq k_d, \tag{16}$$

$$\Leftrightarrow \sum_{(i,j) \in \Omega_k} \mu_{i,j,k} V_{\text{voxel}} = \text{constant}, \ \ k \geq k_d. \tag{17}$$

Eqs. (16) and (17) were only defined for $k \geq k_d$ since they were derived using eq. (12). Let's consider eq. (17) between the rescaled MHR map from source scan $k = k_d$ onto target scan $k = k_f$, and the source MHR map at scan $k = k_d$:

$$\sum_{(i,j) \in \Omega_{k_f}} \mu^F_{i,j,k_d \to k_f} V_{\text{voxel}} \cdot \gamma = \sum_{(i,j) \in \Omega_{k_d}} \mu_{i,j,k} V_{\text{voxel}}, \tag{18}$$

$$\Rightarrow \bar{\mu}^F_{k_d\to k_f} V_{k_f} \cdot \gamma = \bar{\mu}_{k_d} V_{k_d}, \tag{19}$$

$$\Leftrightarrow \bar{\mu}^F_{k_d\to k_f} \cdot \gamma = \bar{\mu}_{k_d} \cdot \frac{V_{k_d}}{V_{k_f}}, \tag{20}$$

where $\bar{\mu}_k$ is the RAC averaged over the domain Ω_k. Since the average of all values of a MHR map (or any digital image) are conserved upon interpolation, we have that:

$$\bar{\mu}^F_{k_d\to k_f} = \bar{\mu}_{k_d}, \tag{21}$$

giving an expression for the scaling factor γ:

$$\gamma \equiv \gamma_{k_d\to k_f} = \frac{V_{k_d}}{V_{k_f}}. \tag{22}$$

The expression of the γ scaling factor can be generalized:

$$\gamma_{\mathrm{source}\to\mathrm{target}} = \frac{V_{\mathrm{source}}}{V_{\mathrm{target}}}. \tag{23}$$

The rescaled maps were averaged over the scans $k \geq k_d$, resulting in an artificial MHR map representative of the dry gel:

$$\mu^{\mathrm{dry}}_{i,j,k_f} = \frac{1}{N_d}\left(\mu_{i,j,k_f} + \sum_{k_d\leq k<k_f} \mu^F_{i,j,k_d\to k_f} \cdot \gamma_{k\to k_f}\right), \tag{24}$$

where N_d is the number of scans in $k_d \leq k \leq k_f$. The quantity $\mu^{\mathrm{dry}}_{i,j,k_f}$ was referred to as the dry MHR map. By recalling eq. (12), the MHR maps $\mu_{i,j,k\geq k_d}$ were equal to $\mu_{\mathrm{skel}} f_{\mathrm{skel},i,j,k\leq k_d}$ under the assumption of a zero hexane content in the gels at $k \geq k_d$. The dry MHR map derived in eq. (24) was thus an expression of the quantity $\mu_{\mathrm{skel}} f_{\mathrm{skel},i,j,k}$ interpolated onto scan $k = k_f$. We set:

$$\mu_{\mathrm{skel}} f_{\mathrm{skel},i,j,k_f} = \mu^{\mathrm{dry}}_{i,j,k_f}. \tag{25}$$

The dry MHR map was then used to calculate the HEXHR maps. Let's recall the expression of the local RAC:

$$\mu_{i,j,k} = \mu_{\mathrm{hex}} f_{\mathrm{hex},i,j,k} + \mu_{\mathrm{skel}} f_{\mathrm{skel},i,j,k}. \tag{4}$$

At $k = k_f$, the rightmost term in eq. (4) is equal to the dry MHR map. The change of the gel volume throughout drying implied that $\Omega_{k\neq k_f} \neq \Omega_{k_f}$. By assuming that the distribution of the silica skeleton within the gel's volume does not change throughout drying, an expression for $\mu_{\mathrm{skel}} f_{\mathrm{skel},i,j,k}$ for any scan k could be obtained by rescaling $\mu_{\mathrm{skel}} f_{\mathrm{skel},i,j,k_f}$ (which is known) from source scan $k = k_f$ to target scan $k \neq k_f$ using a similar methodology as before. In this case, the source is unique ($k = k_f$) and the targets are multiple ($k \neq k_f$). We thus set:

$$\mu_{\text{skel}} f^*_{\text{skel},i,j,k_f \to k} = F_{k_f \to k}\left(\mu_{\text{skel}} f_{\text{skel},i,j,k_f}\right) \cdot \gamma_{k_f \to k} \tag{26}$$

$$\Leftrightarrow \mu_{\text{skel}} f^*_{\text{skel},i,j,k_f \to k} = F_{k_f \to k}\left(\mu^{\text{dry}}_{i,j,k_f}\right) \cdot \gamma_{k_f \to k}, \tag{27}$$

$$\Leftrightarrow \mu_{\text{skel}} f^*_{\text{skel},i,j,k_f \to k} = \mu^{\text{dry},F}_{i,j,k_f \to k} \cdot \gamma_{k_f \to k}, \tag{28}$$

where $\mu_{\text{skel}} f^*_{\text{skel},i,j,k}$ is the rescaled map and the scaling factor is:

$$\gamma_{k_f \to k} = \frac{V_{k_f}}{V_k}. \tag{29}$$

The expression for the scaling factor was verified by recalling the conservation of the skeleton volume, considering the rescaled map at scan $k \neq k_f$ and the dry MHR map at scan $k = k_f$:

$$\sum_{(i,j)\in\Omega_k} f_{\text{skel},i,j,k} V_{\text{voxel}} = constant, \tag{6}$$

$$\Rightarrow \sum_{(i,j)\in\Omega_k} \frac{\mu^{\text{dry},F}_{i,j,k_f \to k} \cdot \gamma_{k_f \to k}}{\mu_{\text{skel}}} V_{\text{voxel}} = \sum_{(i,j)\in\Omega_{k_f}} \frac{\mu^{\text{dry}}_{i,j,k_f}}{\mu_{skel}} V_{\text{voxel}}, \tag{30}$$

$$\Rightarrow \bar{\mu}^{dry,F}_{k_f \to k} \cdot \gamma_{k_f \to k} V_k = \bar{\mu}^{dry}_{k_f} V_{k_f}, \tag{31}$$

$$\Leftrightarrow \bar{\mu}^{dry,F}_{k_f \to k} = \bar{\mu}^{dry}_{k_f}, \tag{32}$$

where $\bar{\mu}^{\text{dry},F}_{k_f \to k}$ and $\bar{\mu}^{\text{dry}}_{k_f}$ is the RAC of the maps averaged over the domains Ω_k and Ω_{k_f}, respectively. Finally, the quantity $\mu_{\text{skel}} f_{\text{skel},i,j,k}$ in eq. (4) was replaced by the expression in eq. (28), giving an expression for the hexane volume fraction maps at any scan:

$$f_{\text{hex},i,j,k} = \frac{\mu_{i,j,k} - \mu^{\text{dry},F}_{i,j,k_f \to k} \cdot \gamma_{k_f \to k}}{\mu_{\text{hex}}}. \tag{33}$$

Skeleton volume fraction. The SKELHR maps can be directly calculated at $k_1 \leq k \leq k_2$ using the volume conservation equation and the previously computed HEXHR maps. Similarly to the procedure adopted to calculate the hexane volume fraction maps, an artificial MHR map representative of the state of the gel at the beginning of drying was computed to improve the statistics upon calculating $f_{\text{skel},i,j,k}$. This was done by rescaling and combining the MHR maps over $k_1 \leq k \leq k_2$. The rescaling was performed from source scans $k_1 < k \leq k_1$ onto target scan $k = k_1$. In this case, the target is unique and the sources are multiple. The rescaled maps are:

$$\mu^*_{i,j,k \to k_1} = \mu^F_{i,j,k \to k_1} \cdot \gamma_{k \to k_1} + \beta, \;\; k_1 \leq k \leq k_2, \tag{34}$$

where $\mu^F_{i,j,k\to k_1}$ is the interpolated map defined over the domain Ω_{k_1} and β is an additional scaling factor. An expression for β was found by considering a quantity that stays constant over $k_1 \le k \le k_2$ (similar strategy as the one employed to determine γ). Let's recall the 2nd conservation equation:

$$\sum_{(i,j)\in\Omega_k} \mu_{i,j,k} - \mu_{\text{hex}} = \text{constant}, \quad k_1 \le k \le k_2. \tag{11}$$

Let's consider eq. (11) between a rescaled MHR map from scan $k \neq k_1$ to $k = k_1$ and the MHR map of source scan k:

$$\sum_{(i,j)\in\Omega_{k_1}} \mu^*_{i,j,k\to k_1} - \mu_{\text{hex}} = \sum_{(i,j)\in\Omega_k} \mu_{i,j,k} - \mu_{\text{hex}}, \tag{35}$$

$$\Leftrightarrow V_{\text{voxel}} \sum_{(i,j)\in\Omega_{k_1}} \mu^F_{i,j,k\to k_1} \cdot \gamma_{k\to k_1} + \beta - \mu_{\text{hex}} = V_{\text{voxel}} \sum_{(i,j)\in\Omega_k} \mu_{i,j,k} - \mu_{\text{hex}}, \tag{36}$$

$$\Rightarrow \left(\bar{\mu}^F_{k\to k_1} \cdot \gamma_{k\to k_1} + \beta - \mu_{\text{hex}}\right) V_{k_1} = (\bar{\mu}_k - \mu_{\text{hex}}) V_k, \tag{37}$$

where $\bar{\mu}^F_{k\to k_1}$ and $\bar{\mu}_k$ are the MHR maps averaged over their domain Ω_{k_1} and Ω_k, respectively. Because bilinear interpolation conserves the average value of a MHR map, we have that:

$$\bar{\mu}^F_{k\to k_1} = \bar{\mu}_k, \tag{38}$$

giving an expression for β:

$$\left(\bar{\mu}^F_{k\to k_1} \cdot \frac{V_k}{V_{k_1}} + \beta - \mu_{\text{hex}}\right) V_{k_1} = (\bar{\mu}_k - \mu_{\text{hex}}) V_k, \tag{39}$$

$$\Leftrightarrow \bar{\mu}^F_{k\to k_1} V_k + (\beta - \mu_{\text{hex}}) V_{k_1} = \bar{\mu}_k V_k - \mu_{\text{hex}} V_k, \tag{40}$$

$$\Leftrightarrow (\beta - \mu_{\text{hex}}) V_{k_1} = -\mu_{\text{hex}} V_k, \tag{41}$$

$$\Rightarrow \beta = \mu_{\text{hex}} \frac{\left(V_{k_1} - V_k\right)}{V_{k_1}}, \tag{42}$$

$$\Leftrightarrow \beta = \mu_{\text{hex}}\left(1 - \gamma_{k\to k_1}\right). \tag{43}$$

The rescaled MHR maps were then averaged over $k_1 \le k \le k_2$, resulting in an artificial MHR map representative of the state of the alcogel referred to as the alco MHR map:

$$\mu^{\text{alco}}_{i,j,k_1} = \frac{1}{N_a}\left[\mu_{i,j,k_1} + \sum_{k_1<k\le k_2} \mu^F_{i,j,k\to k_1}\gamma_{k\to k_1} + \mu_{\text{hex}}\left(1 - \gamma_{k\to k_1}\right)\right], \tag{44}$$

where N_a is the number of scans between $k_1 \le k \le k_2$. The alco MHR map was used to compute a hexane volume fraction map representative of the alcogel at scan $k = k_1$. Eq. (33) was rewritten considering a target scan $k = k_1$ and by replacing the MHR map at scan k by the alco MHR map at scan $k = k_1$:

$$f_{\mathrm{hex},i,j,k_1}^{\mathrm{alco}} = \frac{\mu_{i,j,k_1}^{\mathrm{alco}} - \mu_{i,j,k_f\to k_1}^{\mathrm{dry},F} \cdot \gamma_{k_f\to k_1}}{\mu_{\mathrm{hex}}}, \tag{45}$$

where $f_{\mathrm{hex},i,j,k_1}^{\mathrm{alco}}$ is a hexane map representative of the state of the alcogel expressed at scan $k = k_1$. This expression was finally used to calculate the SKELHR map at scan $k = k_1$ from the conservation of the total volume:

$$f_{\mathrm{skel},i,j,k_1} = 1 - f_{\mathrm{hex},i,j,k_1}^{\mathrm{alco}}. \tag{46}$$

The SKELHR maps at scans $k \neq k_1$ were determined by rescaling $f_{\mathrm{skel},i,j,k_1}$:

$$f_{\mathrm{skel},i,j,k} = f_{\mathrm{skel},i,j,k_1\to k}^{F} \cdot \gamma_{k_1\to k}, \tag{47}$$

where $f_{\mathrm{skel},i,j,k_1\to k}^{F}$ is the interpolated map defined over the domain Ω_k.

Vapor/air volume fraction. The vapor/air volume fraction maps were directly computed using eq. (5) with the knowledge of $f_{\mathrm{hex},i,j,k}$ and $f_{\mathrm{skel},i,j,k}$:

$$f_{\mathrm{air},i,j,k} = 1 - f_{\mathrm{hex},i,j,k} - f_{\mathrm{skel},i,j,k}. \tag{48}$$

SI3: Bilinear Interpolation Procedure

A key step in the local quantitative imaging procedure was the resizing of the maps between the different drying stages by bilinear interpolation. The resizing and rescaling of a GHR map from source scan $k_s = 140$ onto target scan $k_t = 0$ is illustrated in Figure S3 as an example and consisted in: (i) conversion of the GHR maps into MHR maps (Figure S3b), (ii) define the edges of the domains Ω_{k_s} and Ω_{k_t} separating the sample from the background in the source and target MHR maps (Figure S3c), (iii) compute the normalized vertical and radial coordinates of any pixel of the maps within the domain edges (not shown), (iv) bilinear interpolation of source map onto target map and correction of the RAC values by a scalar factor (Figure S3d).

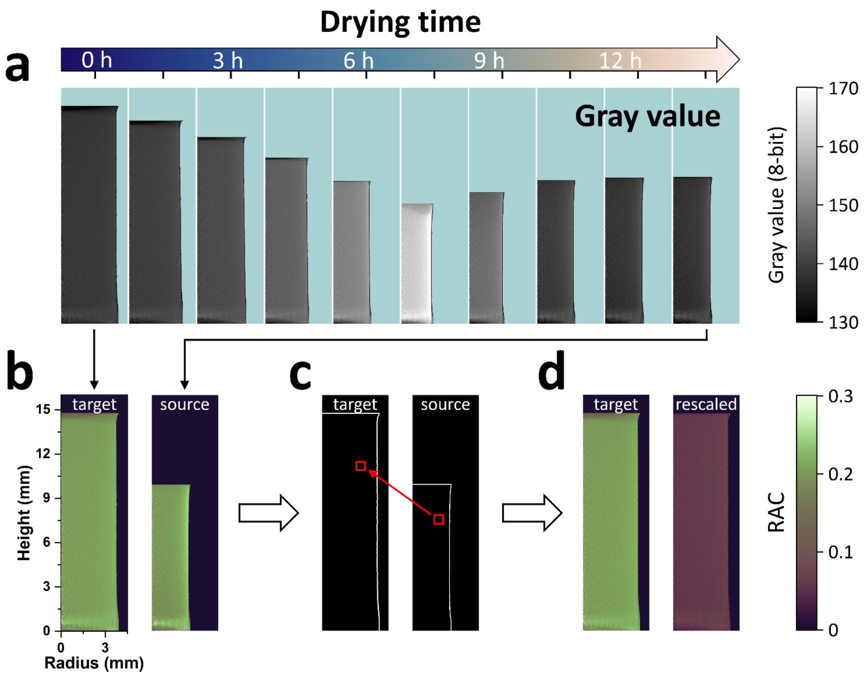

Figure S3. Example of the bilinear interpolation procedure and rescaling. (a) GHR maps of sample M4 at 10 selected drying stages on top of a cyan background with the corresponding color scale on the right. The brightness and contrast in the images of the gray value maps was adjusted to improve visualization. (b) MHR maps converted from the GHR maps at the start of drying (target scan) and at the end of drying (source scan) (c) Domains defining the sample in the MHR maps (white line over black background) in target and source scans. The red squares illustrate the correspondence in the relative coordinates of two pixels in both scans. (d) Target MHR map and rescaled MHR map interpolated from the source scan domain onto the target scan domain. The color scale of the MHR maps in panels (b) and (d) is shown at the bottom right of the figure. The length axes of all maps is indicated in the first map of panel (b).

As mentioned in the main text and in SI2, the domain of two GHR or MHR maps taken at different scans did not match, due to the shape change of the sample. To compare maps at different drying stages required establishing a correspondence between the two domains, which was done by bilinear interpolation of a source scan: k_s towards the domain of a target scan: k_t. The procedure described here is based on the GHR maps but can also be applied to a MHR map or a volume fraction map upon minor adaptations. First, the edges of the domains were defined as the limit where the gray values dropped to zero in the GHR maps (Figure S3c). The top edge of the gel was called the north edge: $N_{j,k}$, and was defined as the i index where the gray values became non-zero, from top to bottom for each index j. The radial edge of the gel was called the east edge: $E_{i,k}$, and was similarly defined as the j index where the gray values became non-zero, from right to left for each index i. The bottom edge of the gel was called the south edge: S and was independent of the radial position and of the scan number. Figure S4 depicts those edges of the GHR maps at scans k_t and k_s shown in Figure S3.

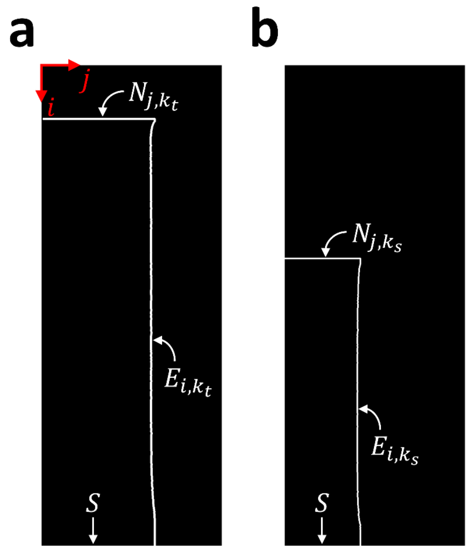

Figure S4. Edges (white lines) of the domains of two GHR maps from (a) a target scan at the start of drying (k=0) and (b) a source scan at the end of drying (k=140). The north edges are depicted with an arrow: N_{j,k_t} and N_{j,k_s} for target and source scans, respectively. The east edges are depicted accordingly: E_{i,k_t} and E_{i,k_s}. The south edge S is also shown and is independent of the j index and of the scan number k. The axes are shown in red on the top left of the figure in panel (a).

We define the normalize coordinates within a domain Ω_k as:

$$h_{i,j} = \frac{i - N_{j,k}}{S - N_{j,k}}, \tag{49}$$

$$r_{i,j} = \frac{j}{E_{i,k}}, \tag{50}$$

With $h_{i,j} \in [0,1]$ and $r_{i,j} \in [0,1]$. For a given coordinate in the target map -abbreviated (i^*, j^*)- a corresponding coordinate was calculated in the source map -abbreviated (i, j)- by minimizing the difference between the normalized coordinates, defined as $\delta_h = |h^* - h|$ and $\delta_r = |r^* - r|$. (h^*, r^*) is short for $(h_{i^*,j^*}, r_{i^*,j^*})$ and is the normalized coordinates of the target map (known). (h, r) is short for $(h_{i,j}, r_{i,j})$ and is the normalized coordinate of the source map (unknown). The following algorithm was developed to find (h, r) at a given (h^*, r^*):

1) Define the vertical and radial tolerance:

$$\epsilon_h = 0.1 \cdot (S - N_{k_s}^{\min}), \tag{51}$$

$$\epsilon_r = 0.1 \cdot E_{k_s}^{\max}, \tag{52}$$

where $N_{k_s}^{\min}$ is the minimum of N_{j,k_s} and $E_{k_s}^{\max}$ is the maximum of E_{i,k_s}.

2) Set a starting point in the source map (h_0, r_0) as:

$$h_0 = \lfloor h^* \cdot (S - N_{k_s}^{\min}) + N_{k_s}^{\min} \rceil, \tag{53}$$

$$r_0 = 0, \tag{54}$$

where the notation $\lfloor x \rceil$ stands for rounding x to the nearest integer (0.5 is rounded down to 0).

3) Compute the normalized radial coordinate in the source map r_1 the closest to the target coordinate r^* at $h = h_0$:

$$r_1 = \underset{i|h_{i,j}=h_0}{\operatorname{argmin}}\left(\left|r^* - r_{i,j}\right|\right). \tag{55}$$

The function argmin is the argument of the minima taken over a series of coordinates (i,j), where i is such that $h_{i,j} = h_0$. This represents a relatively horizontal line.

4) Do the same for the normalized vertical coordinate h_1 at $r = r_1$:

$$h_1 = \underset{j|r_{i,j}=r_1}{\operatorname{argmin}}\left(\left|h^* - h_{i,j}\right|\right), \tag{56}$$

where the coordinates j is such that $r_{i,j} = r_1$.

5) Compute the difference $\delta_h = |h^* - h_1|$ and $\delta_r = |r^* - r_1|$.

6) If $\delta_h \geq \epsilon_h$ or $\delta_r \geq \epsilon_r$, assign the current value of h_1 to h_0 and r_1 to r_0, and repeat steps 3) to 6). After five iterations, or if $\delta_h < \epsilon_h$ and $\delta_r < \epsilon_r$, set $h = h_1$ and $r = r_1$ and exit the loop.

Once the correspondence between the normalized coordinates in the source map (h,r) and in the target map (h^*, r^*) was established, an empty map with the domain of the target map Ω_{k_t} was created. The gray values in the empty map were filled using a bilinear interpolation algorithm. For any pixel with the coordinates (i^*, j^*) in the empty map, its gray value was determined by:

1) Calculate the virtual position in the source map corresponding to the normalized coordinates (h^*, r^*) of the empty map:

$$I = h^* \cdot (S - N_{j,k_s}) + N_{j,k_s}, \tag{57}$$

$$J = r^* \cdot E_{i,k_s}, \tag{58}$$

where I and J are not pixel indexes but a virtual position in between pixels. (i,j) are known from $(h_{i,j}, r_{i,j})$ determined in the previous calculations.

2) Find the four pixels the closest to the virtual position (I,J):

$$i_1 = \begin{cases} i, & I \geq i \\ i-1, & I < i \end{cases}, \tag{59}$$

$$i_2 = \begin{cases} i+1, & I \geq i \\ i, & I < i \end{cases}, \tag{60}$$

$$j_1 = \begin{cases} j, & J \geq j \\ j-1, & J < j \end{cases}, \tag{61}$$

$$j_2 = \begin{cases} j+1, & J \geq j \\ j, & J < j \end{cases}. \tag{62}$$

3) The gray value in the empty map at the coordinates (i^*, j^*) is given by the bilinear interpolation formula:

$$g_{i^*,j^*,k_s \to k_t} = \begin{pmatrix} i_2 - I & I - i_1 \end{pmatrix} \begin{pmatrix} g_{i_1,j_1,k_s} & g_{i_1,j_2,k_s} \\ g_{i_2,j_1,k_s} & g_{i_2,j_2,k_s} \end{pmatrix} \begin{pmatrix} j_2 - J \\ J - j_1 \end{pmatrix}. \tag{63}$$

By construction, the interpolated map $g_{i^*,j^*,k_s \to k_t}$ was defined over the same domain as the target map g_{i,j,k_t}. After interpolation, depending on the nature of the interpolated map (GHR, MHR or volume fraction map), the interpolated map was corrected with one or more scaling factor (see SI2).

Figure S5

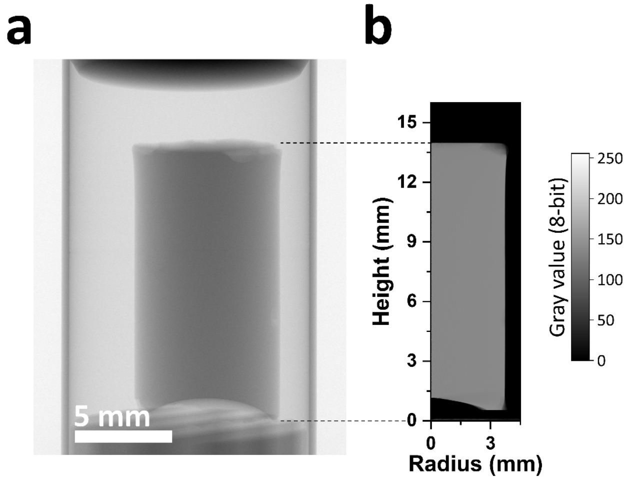

Figure S5. (a) µCT projection of sample M3 at the start of drying, the meniscus in the gel is visible at the gel's bottom. (b) GHR map of the same µCT scan on a black background, where artifacts can be seen at the bottom of the sample. Those artifacts resulted in errors in the bilinear interpolation algorithm, preventing to compute volume fraction maps for that sample.

SI4: Gel Diameter during X-ray scattering measurements

Here we describe how the local diameter of the gels was calculated during their drying at the µSpot beamline at BESSY. The digital pictures were correlated with the µCT data and with the time stamps of the scattering data. Figure S6 shows a series of digital pictures at selected drying stages, where the maximum shrinkage occurred after ca. 4.4 h of drying, which was faster than in the µCT experiments. Due to the insufficient contrast in the image, the dimensions of the gel could not be retrieved by automated image processing. Instead, the height of the gel in pixel was measured manually from 14 pictures using the

software Fiji [4]. It must be noted that during the first 2 h of drying, the height of the gel could not be measured because its top was hidden by the measurement cell (Figure S6).

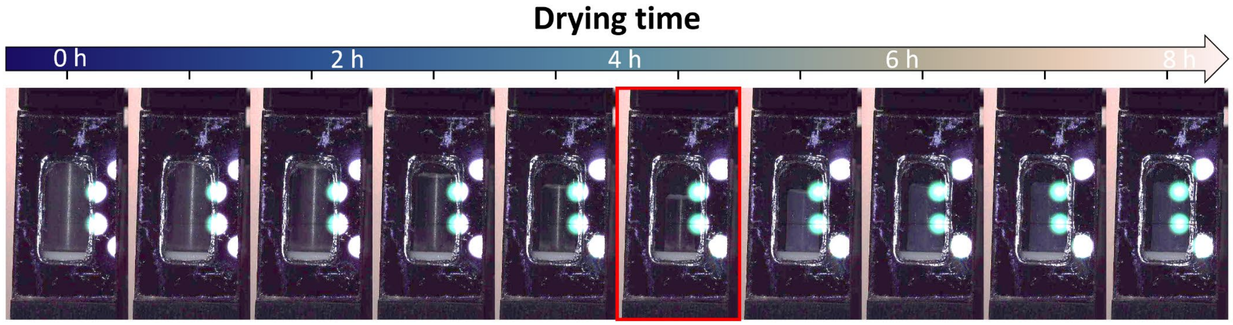

Figure S6. Digital pictures of the gel dried in the measurement cell during the in operando SAXS/WAXS measurement. The picture corresponding to the maximum shrinkage is outlined in red. The bright spots are due to the reflection of the camera lamp on the museum glass. The brightness and contrast of the images are adjusted for better visualization.

The diameter of the gel in pixel was derived by dividing the height by a factor 2 (gels have an aspect ratio of 2 [2]) and was normalized by the diameter of the gel at the maximum shrinkage. We define:

$$\nu_{\mathrm{PI}}(t_{\mathrm{PI}}) = d(t_{\mathrm{PI}})/d\big(t_{\mathrm{PI}} = t_{\mathrm{PI,MS}}\big), \tag{64}$$

with $\nu_{\mathrm{PI}}(t_{\mathrm{PI}})$ the normalized diameter from the digital pictures, $d_{\mathrm{PI}}(t_{\mathrm{PI}})$ the diameter of the gel in pixels and $t_{\mathrm{PI,MA}}$ the time of maximum shrinkage. t_{PI} is the time at which the digital pictures were recorded. $\nu_{\mathrm{PI}}(t_{\mathrm{PI}})$ is shown in Figure S7. Because the diameter of the gel at $t_{\mathrm{PI}} < 2\ \mathrm{h}$ could not be measured, the diameter from the digital pictures was fitted using µCT data, allowing to extrapolate the diameter of the gel at the start of drying.

During the in operando X-ray scattering experiment, the X-ray beam probed the gel at a vertical height of ca. 4 mm from the bottom of the gel. That location can be seen in Figure S6 as a slight darker line on the museum glass. The diameter of the gels dried by µCT at the same location was extracted from the masked images and was normalized by its value at the maximum shrinkage as in eq. (64). The corresponding normalized diameters of samples M1, M2, M4 and M5 are shown in Figure S7. Note that the drying rate within these samples differed because of slightly different starting volume of the gels and possibly drying conditions.

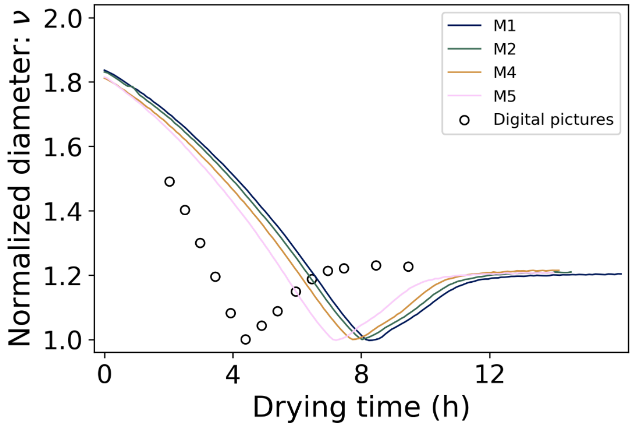

Figure S7. Diameter of a gel normalized over its diameter at the maximum shrinkage: ν from the μCT measurements (M1, M2, M4 and M5) and from the digital pictures versus the drying time.

The μCT normalized diameter was taken as the average among samples M1, M2, M3 and M4 to improve the accuracy of the fit. To do so, the μCT time scale was also normalized:

$$x_{\mathrm{CT},m} = t_{\mathrm{CT},m} \,/\, t_{\mathrm{CT,MS},m}, \quad (65)$$

where $x_{\mathrm{CT},m}$ is a time scale normalized over the time of the maximum shrinkage $t_{\mathrm{CT,MS},m}$ for a sample m. The average μCT normalized diameter was then: $\nu_{\mathrm{CT}}(x_{\mathrm{CT}})$, where $x_{\mathrm{CT}} = 0$ and $x_{\mathrm{CT}} = 1$ corresponded to the start of drying and to the maximum shrinkage, respectively.

The normalized diameter from the digital pictures was also expressed as a function of a normalized time scale. However, the first recorded image was taken a few minutes after the gel started drying due to experimental limitations. We defined the time difference between the first recorded image and the effective start of drying of the gel as Δt_1. Therefore, we can express $\nu_{\mathrm{PI}} = \nu_{\mathrm{PI}}(x_{\mathrm{PI}})$ with x_{PI} as:

$$x_{\mathrm{PI}} = \frac{t_{\mathrm{PI}} + \Delta t_1}{t_{\mathrm{PI,MS}} + \Delta t_1}. \quad (66)$$

By minimizing the difference of $\left(\nu_{\mathrm{CT}}(x_{\mathrm{CT}}) - \nu_{\mathrm{PI}}(x_{\mathrm{PI}})\right)^2$, Δt_1 could be determined at ca. 3.5 min, which was reasonable. After a time scale normalization, $\nu_{\mathrm{PI}}(x_{\mathrm{PI}})$ was in good agreement with the normalized diameter from the μCT data (see Figure S8). $\nu_{\mathrm{PI}}(x_{\mathrm{PI}})$ was then interpolated at the points x_{CT}.

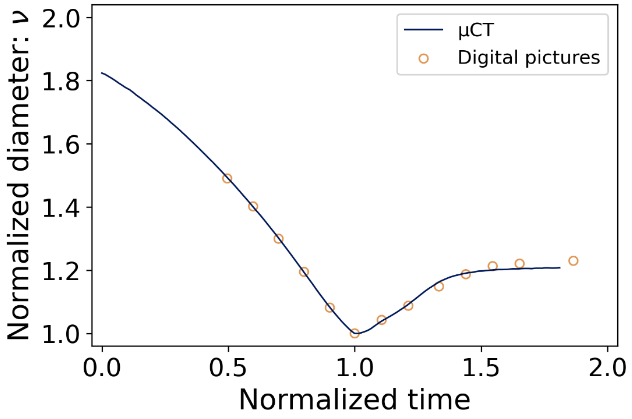

Figure S8. Normalized diameter ν from the μCT and digital pictures expressed as a function of a normalized time scale.

The last step was to interpolate the diameter of the gel with the time of the scattering data. The scattering intensity could be expressed as $I(q, x_{\mathrm{XS}})$ with:

$$x_{\mathrm{XS}} = \frac{t_{\mathrm{XS}} + \Delta t_2}{t_{\mathrm{XS,MS}} + \Delta t_2}. \tag{67}$$

where x_{XS} is the normalized time scale of the X-ray scattering data frames, t_{XS} the time in h, $t_{\mathrm{XS,MS}}$ the time of maximum shrinkage and Δt_2 a time shift. $t_{\mathrm{XS,MS}}$ was set by visual inspection of the scattering profiles (see Figure S19). Δt_2 was known, it corresponded to the delay between the first scattering data frame recorded and the first digital pictures that was taken. $\nu_{\mathrm{PI}}(x_{\mathrm{PI}})$ was then interpolated at the points x_{XS}. The diameter in absolute units was calculated with: $d_{\mathrm{XS}}(x_{\mathrm{XS}}) = \nu_{\mathrm{PI}}(x_{\mathrm{XS}}) \cdot d_{\mathrm{CT,MS}}$, from which the diameter of the gel during drying was found by converting x_{XS} back to the absolute time scale t_{XS}.

Figure S9

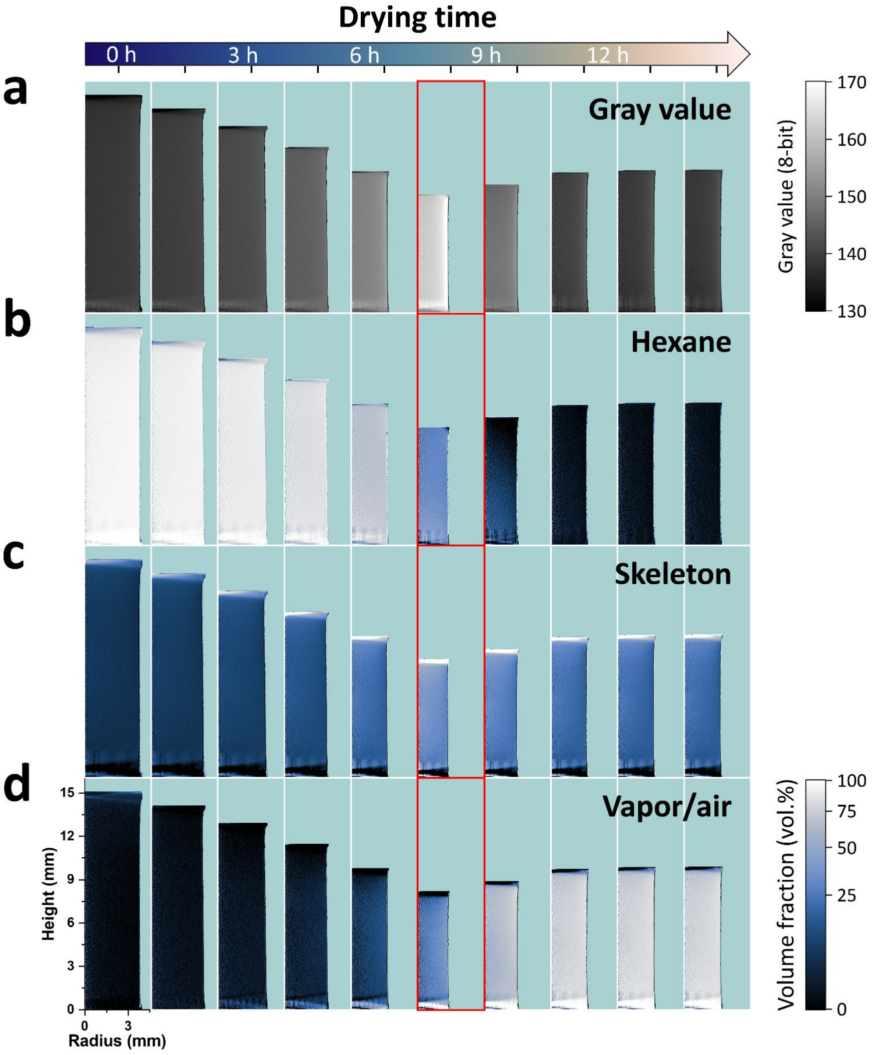

Figure S9. GHR and volume fraction maps of sample M1 at 10 selected drying stages on top of a cyan background. (a) GHR maps with the corresponding gray value scale on the right. The brightness and contrast in the images of the GHR maps are adjusted to improve visualization. (b) HEXHR maps. (c) SKELHR maps. (d) AIRHR maps. The color scale of the volume fraction maps is shown at the bottom right of the figure. The volume fraction maps are normalized between 0 % and 100 %. The images of the volume fraction maps are encoded with a gamma value of 0.5 to improve visualization. The time scale is illustrated with an arrow on top of the figure and the time gap between the maps in a given panel is 1.64 +/- 0.04 h. The length scale of all maps is indicated in the first map of panel (d). The maps corresponding to the maximum shrinkage are outlined in red. Each map consists in 410 x 1455 non-interpolated data points.

Figure S10

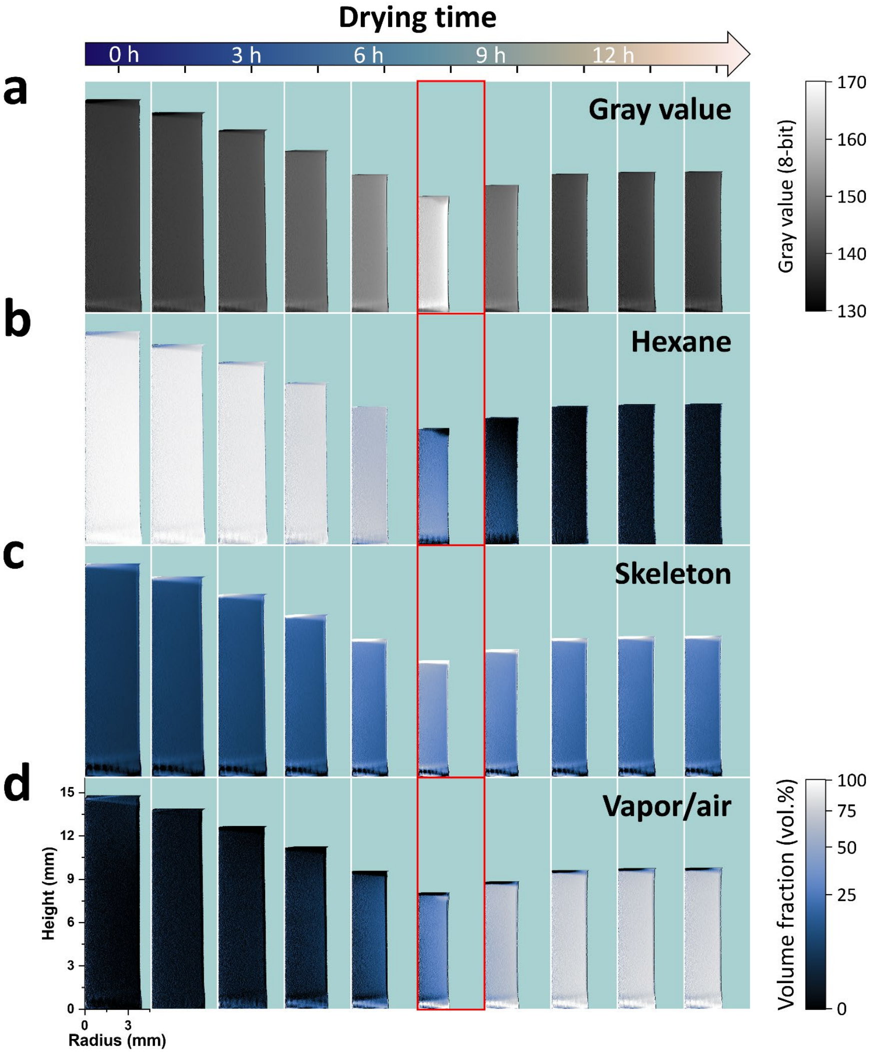

Figure S10. GHR and volume fraction maps of sample M2 at 10 selected drying stages on top of a cyan background. (a) GHR maps with the corresponding gray value scale on the right. The brightness and contrast in the images of the GHR maps are adjusted to improve visualization. (b) HEXHR maps. (c) SKELHR maps. (d) AIRHR maps. The color scale of the volume fraction maps is shown at the bottom right of the figure. The volume fraction maps are normalized between 0 % and 100 %. The images of the volume fraction maps are encoded with a gamma value of 0.5 to improve visualization. The time scale is illustrated with an arrow on top of the figure and the time gap between the maps in a given panel is 1.63 +/- 0.04 h. The length scale of all maps is indicated in the first map of panel (d). The maps corresponding to the maximum shrinkage are outlined in red. Each map consists in 410 x 1455 non-interpolated data points.

Figure S11

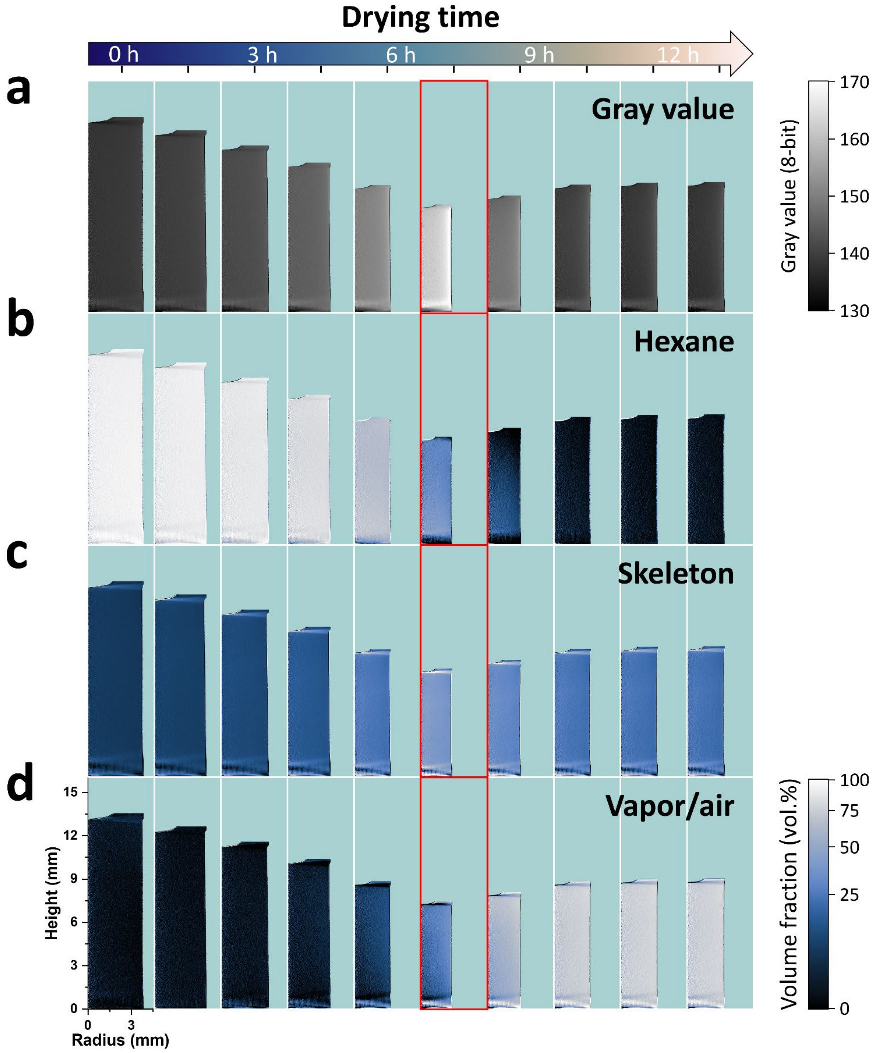

Figure S11. GHR and volume fraction maps of sample M5 at 10 selected drying stages on top of a cyan background. (a) GHR maps with the corresponding gray value scale on the right. The brightness and contrast in the images of the GHR maps are adjusted to improve visualization. (b) HEXHR maps. (c) SKELHR maps. (d) AIRHR maps. The color scale of the volume fraction maps is shown at the bottom right of the figure. The volume fraction maps are normalized between 0 % and 100 %. The images of the volume fraction maps are encoded with a gamma value of 0.5 to improve visualization. The time scale is illustrated with an arrow on top of the figure and the time gap between the maps in a given panel is 1.43 +/- 0.04 h. The length scale of all maps is indicated in the first map of panel (d). The maps corresponding to the maximum shrinkage are outlined in red. Each map consists in 410 x 1455 non-interpolated data points.

Figure S12

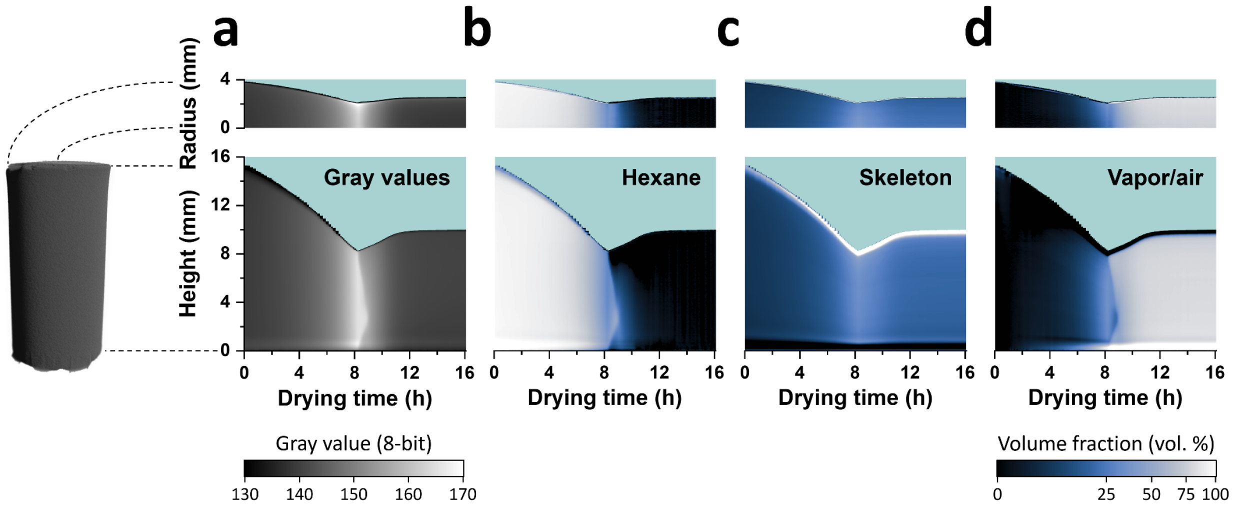

Figure S12. Radial and vertical maps of the gray values and volume fraction of sample M1 on top of a cyan background. The 3D image on the left of the figure depicts the segmented volume of M1 at the beginning of drying and the dashed lines illustrate the radial and vertical axes of the cylinder against which the radial and vertical maps are shown. (a) GR and GH maps. The gray value scale is shown at the bottom of panel (a). The brightness and contrast in the images of the GR and GH maps are adjusted to improve visualization. (b) HEXR and HEXH maps. (c) SKELR and SKELH maps. (d) AIRR and AIRH maps. The images of the volume fraction maps are encoded with a gamma value of 0.5 to improve visualization. The time axis is shown in each vertical map and the length scale is shown in the radial and vertical maps of panel (a). The radial maps consist in 1663 x 410 data points and the vertical maps in 1663 x 1455 points. In all maps, the horizontal time resolution is interpolated from 141 time stamps onto 1663 points.

Figure S13

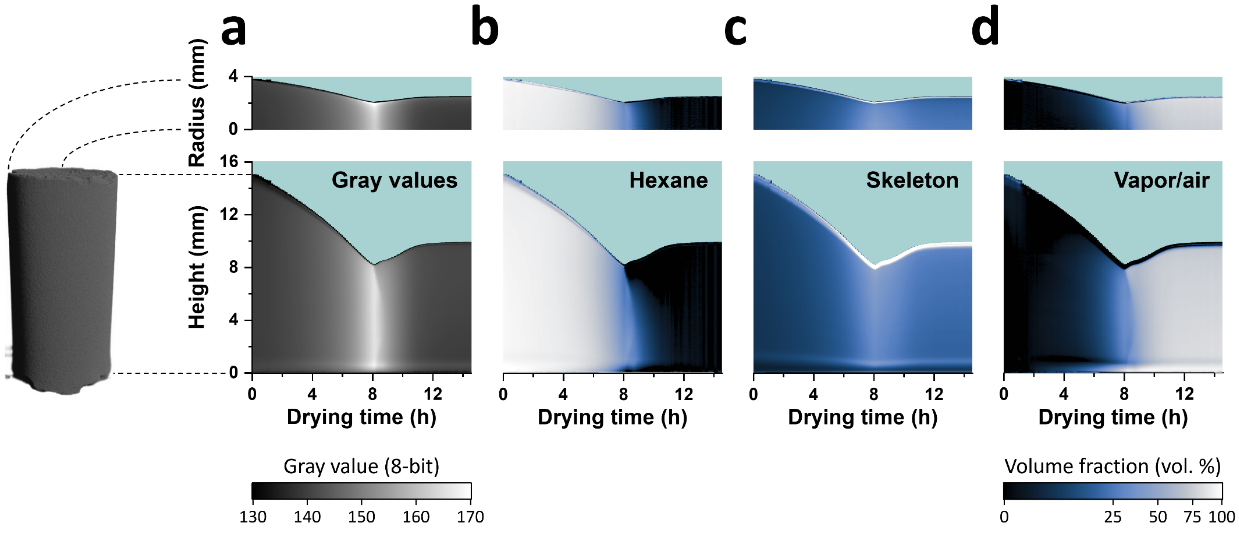

Figure S13. Radial and vertical maps of the gray values and volume fraction of sample M2 on top of a cyan background. The 3D image on the left of the figure depicts the segmented volume of M2 at the beginning of drying and the dashed lines illustrate the radial and vertical axes of the cylinder against which the radial and vertical maps are shown. (a) GR and GH maps. The gray value scale is shown at the bottom of panel (a). The brightness and contrast in the images of the GR and GH maps are adjusted to improve visualization. (b) HEXR

and HEXH maps. (c) SKELR and SKELH maps. (d) AIRR and AIRH maps. The images of the volume fraction maps are encoded with a gamma value of 0.5 to improve visualization. The time axis is shown in each vertical map and the length scale is shown in the radial and vertical maps of panel (a). The radial maps consist in 1509 x 410 data points and the vertical maps in 1509 x 1455 points. In all maps, the horizontal time resolution is interpolated from 141 time stamps onto 1509 points.

Figure S14

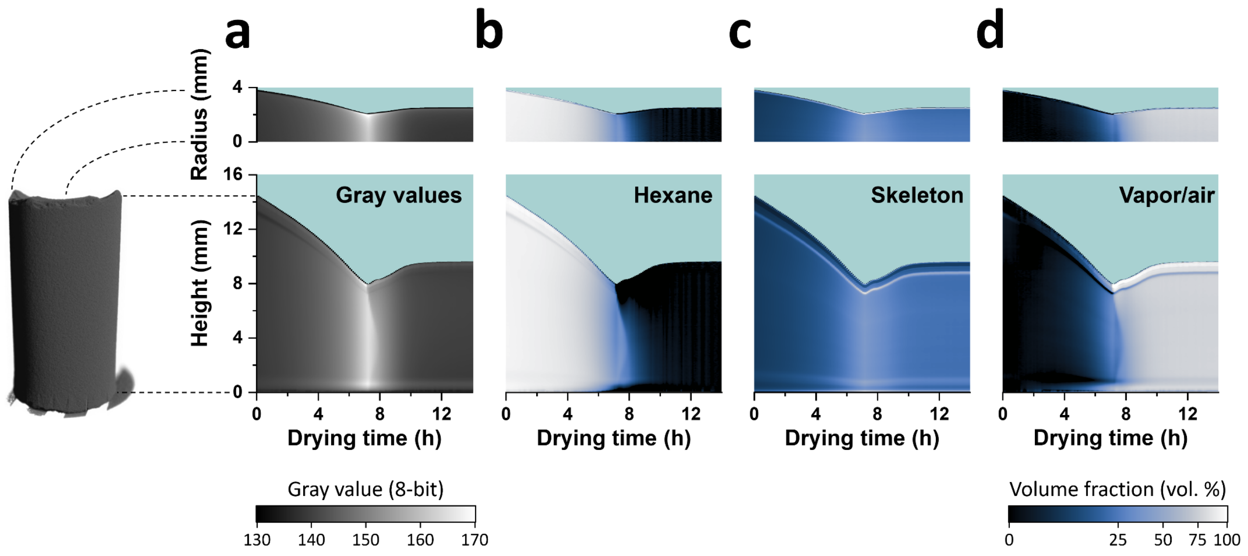

Figure S14. Radial and vertical maps of the gray values and volume fraction of sample M5 on top of a cyan background. The 3D image on the left of the figure depicts the segmented volume of M5 at the beginning of drying and the dashed lines illustrate the radial and vertical axes of the cylinder against which the radial and vertical maps are shown. (a) GR and GH maps. The gray value scale is shown at the bottom of panel (a). The brightness and contrast in the images of the GR and GH maps are adjusted to improve visualization. (b) HEXR and HEXH maps. (c) SKELR and SKELH maps. (d) AIRR and AIRH maps. The images of the volume fraction maps are encoded with a gamma value of 0.5 to improve visualization. The time axis is shown in each vertical map and the length scale is shown in the radial and vertical maps of panel (a). The radial maps consist in 1444 x 410 data points and the vertical maps in 1444 x 1455 points. In all maps, the horizontal time resolution is interpolated from 141 time stamps onto 1444 points.

Figure S15

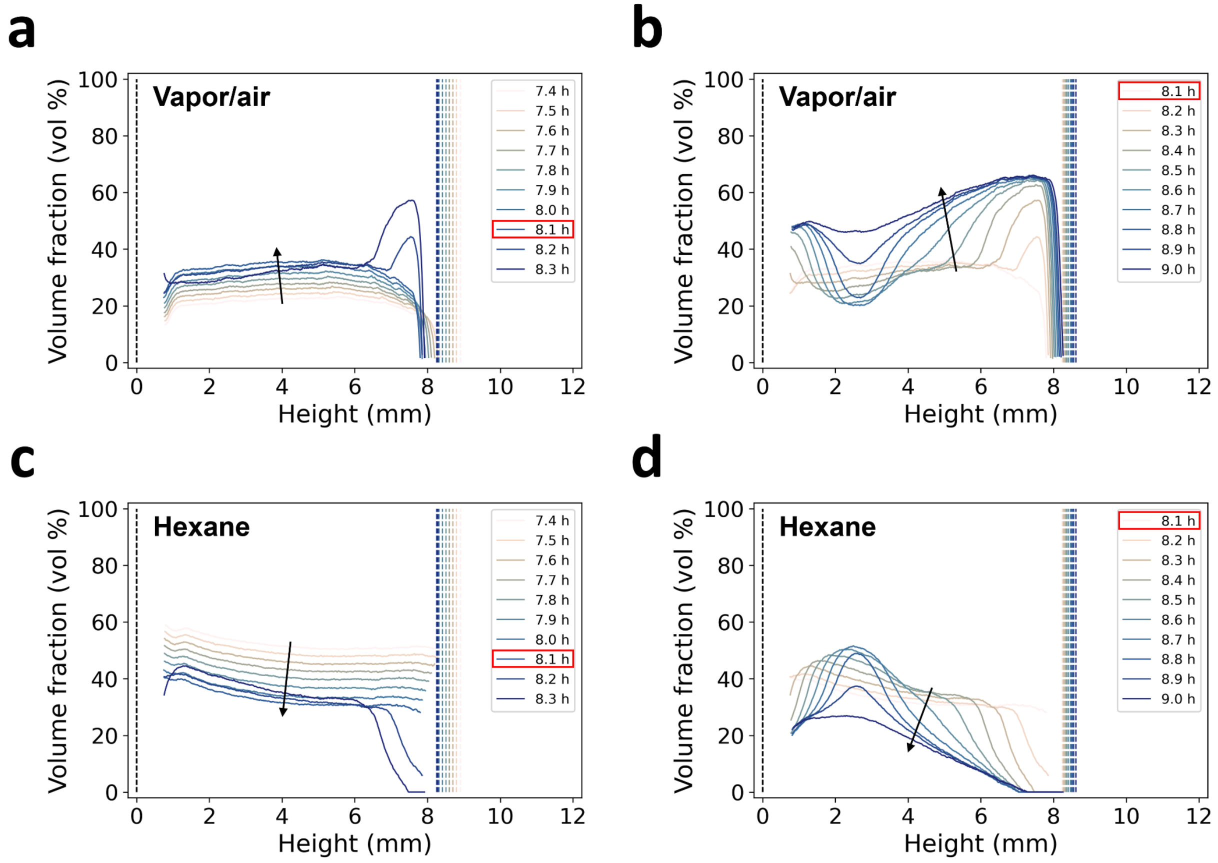

Figure S15. Volume fraction profiles of hexane and vapor/air along the height of sample M1 at selected time stamps. Vapor/air profiles between 7.4 and 8.3 h (a) and between 8.1 and 9.0 h (b). Hexane profiles between 7.4 and 8.3 h (c) and between 8.1 and 9.0 h (d). The profiles in panels (a) and (c) correspond to the µCT scans before the maximum shrinkage and shortly after, while the profiles in panels (b) and (d) correspond to the µCT scans at the maximum shrinkage and after. The dashed lines correspond to the bottom (h = 0 mm) and to the top of the sample. The time of maximum shrinkage is highlighted in red in the legends. The spacing between the profiles in each panel corresponds to a single µCT scan. The black arrows depict the drying time. The profiles were extracted from the AIRH and HEXH maps by excluding the values affected by the artifacts at the edges for better visualization.

Figure S16

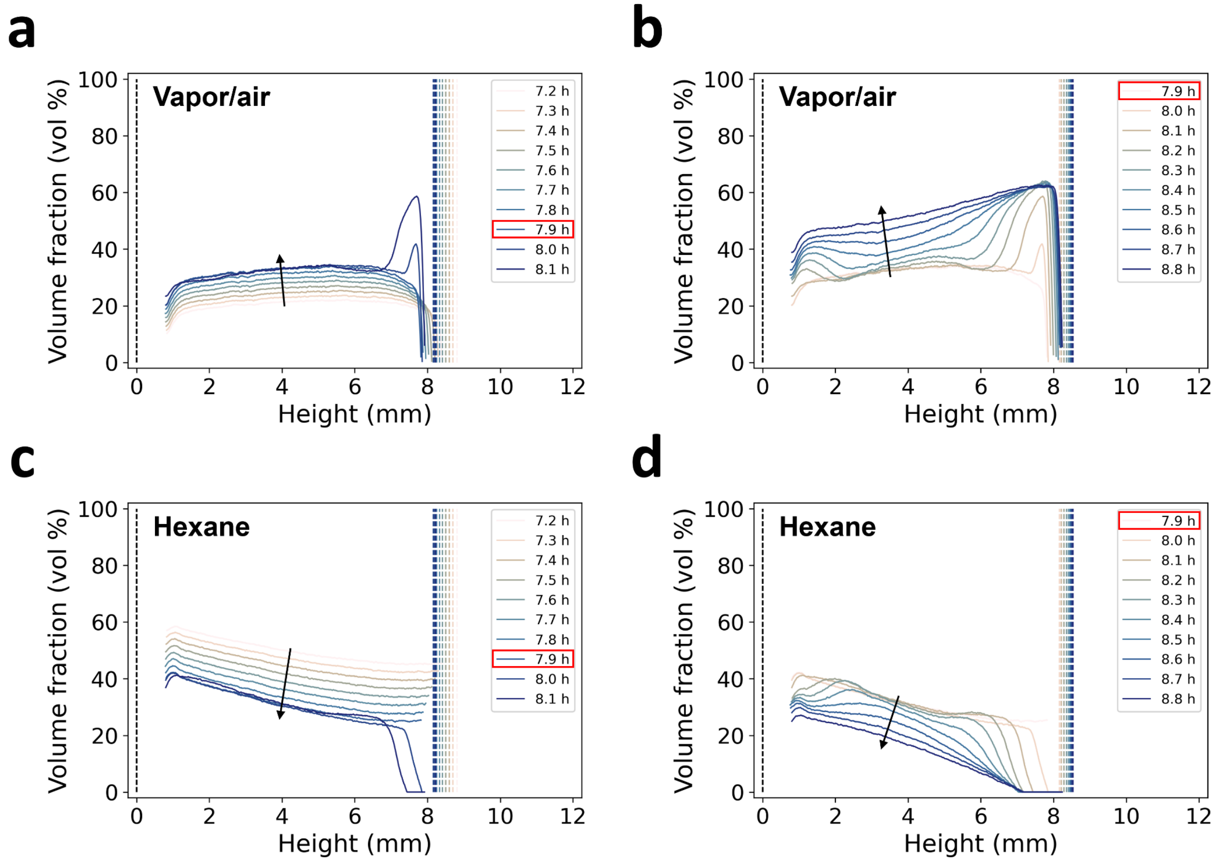

Figure S16. Volume fraction profiles of hexane and vapor/air along the height of sample M2 at selected time stamps. Vapor/air profiles between 7.2 and 8.1 h (a) and between 7.9 and 8.8 h (b). Hexane profiles between 7.2 and 8.1 h (c) and between 7.9 and 8.8 h (d). The profiles in panels (a) and (c) correspond to the µCT scans before the maximum shrinkage and shortly after, while the profiles in panels (b) and (d) correspond to the µCT scans at the maximum shrinkage and after. The dashed lines correspond to the bottom (h = 0 mm) and to the top of the sample. The time of maximum shrinkage is highlighted in red in the legends. The spacing between the profiles in each panel corresponds to a single µCT scan. The black arrows depict the drying time. The profiles were extracted from the AIRH and HEXH maps by excluding the values affected by the artifacts at the edges for better visualization.

Figure S17

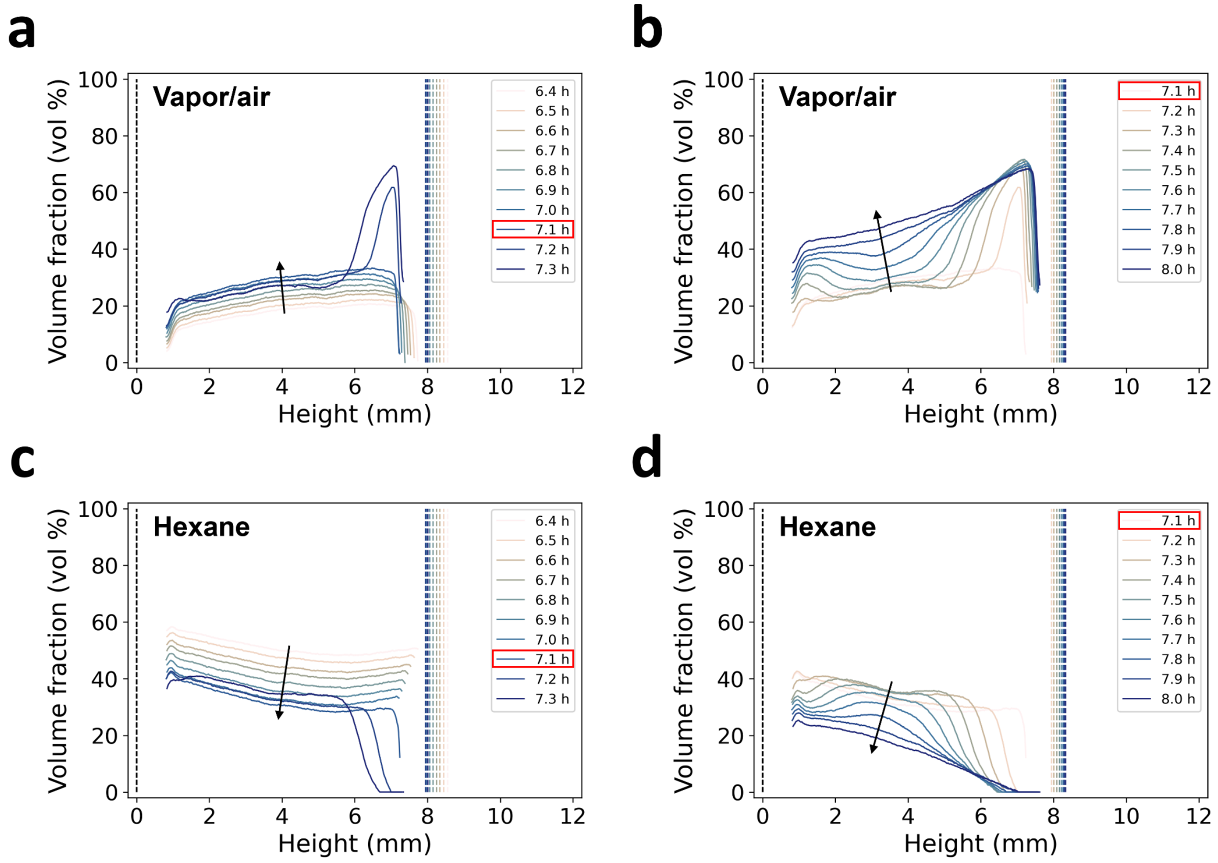

Figure S17. Volume fraction profiles of hexane and vapor/air along the height of sample M5 at selected time stamps. Vapor/air profiles between 6.4 and 7.3 h (a) and between 7.1 and 8.0 h (b). Hexane profiles between 6.4 and 7.3 h (c) and between 7.1 and 8.0 h (d). The profiles in panels (a) and (c) correspond to the µCT scans before the maximum shrinkage and shortly after, while the profiles in panels (b) and (d) correspond to the µCT scans at the maximum shrinkage and after. The dashed lines correspond to the bottom (h = 0 mm) and to the top of the sample. The time of maximum shrinkage is highlighted in red in the legends. The spacing between the profiles in each panel corresponds to a single µCT scan. The black arrows depict the drying time. The profiles were extracted from the AIRH and HEXH maps by excluding the values affected by the artifacts at the edges for better visualization.

Figure S18

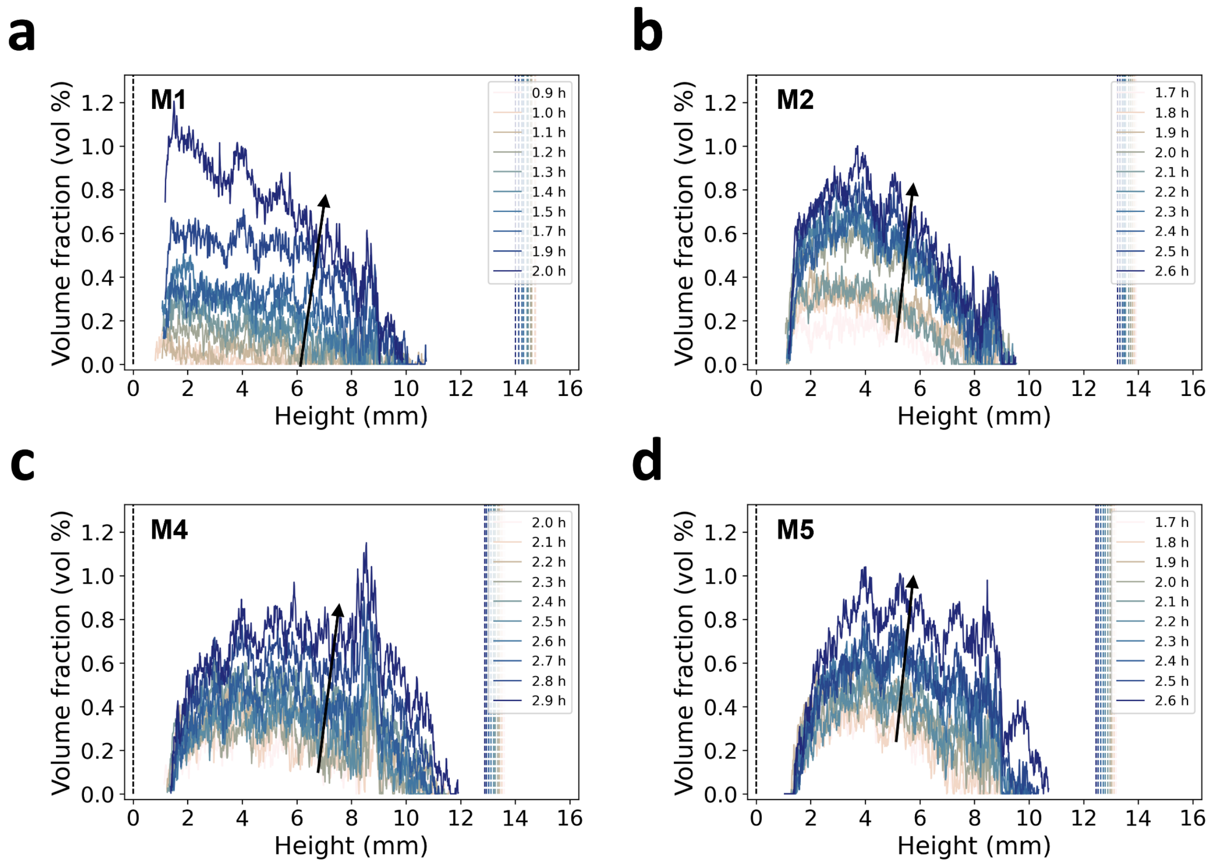

Figure S18. Vertical vapor/air profiles extracted from the AIRH maps of all samples at selected drying stages. (a) Sample M1 between 0.9 and 2.0 h. (b) Sample M2 between 1.7 and 2.6 h. (c) Sample M4 between 2.0 and 2.9 h (as shown in the main text). (d) Sample M5 between 1.7 and 2.6 h. The dashed lines correspond to the bottom (h = 0 mm) and to the top of the sample. The spacing between each profile corresponds to a single μCT scan. The black arrows depict the drying time.

Figure S19

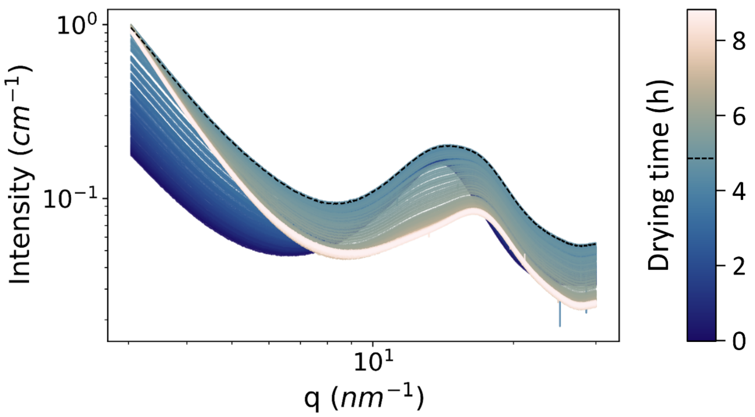

Figure S19. Scattering patterns of gel M6 throughout drying within 3 – 30 nm^{-1}. Only 100 profiles were shown until ca. 8.5 h to enhance visualization. The scattered intensity of the gel at the maximum shrinkage is highlighted with black dashed lines. The color scale is shown on the right and also depicts the time of maximum shrinkage. The data are plotted as bands including the standard deviation.

Figure S20

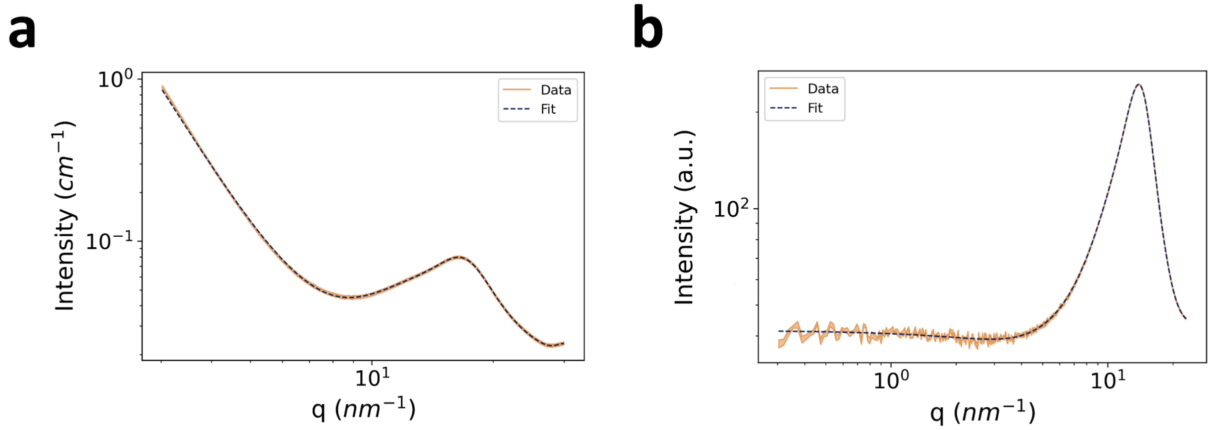

Figure S20. (a) Scattering pattern of sample M6 at the end of drying in the region 3 – 30 nm^{-1} along with the corresponding fit. The scattered intensity is an absolute units. (b) Scattering pattern of a hexane capillary in the region 0.3 – 3.3 nm^{-1} along with the fit of the data. Here the scattered intensity is in arbitrary units as the data frames on the hexane reference samples were not normalized by the sample thickness. The data are plotted as bands including the standard deviation.

Figure S21

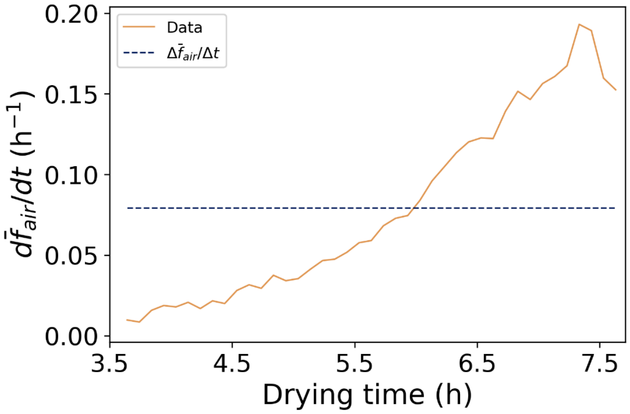

Figure S21. Numerical derivative of the average vapor/air volume fraction in gel M4 versus the drying time: $d\bar{f}_{\mathrm{air}}/dt$ from the cavitation onset ($t_{\mathrm{cav}} = 3.64$ h) until the time of maximum shrinkage ($t_{\mathrm{MS}} = 7.63$ h). The dashed line indicates the value $\Delta\bar{f}_{\mathrm{air}}/\Delta t$ used in the manuscript. These results were produced using the µCT global quantitative imaging results from our previous study [2].

SI5: Spatial Variability Analysis

Vertical and radial distributions. This section reports the spatial variability analysis of the gray values in the GHR and GR maps and discusses their origin. A heterogeneous distribution of the gray values was observed in the GHR maps (Figure 3, Figure S9 – Figure S11). To further quantify these variations, gray value profiles were extracted from the GHR maps of sample M4 across the gel height and radius as shown in Figure S22. Three vertical profiles were extracted at a relative radius of 0.25, 0.5 and 0.75 (from the center of the gel to its radial edge) and three radial profiles were extracted at a relative height of 0.25, 0.5 and 0.75 (from the bottom to the top of the gel).

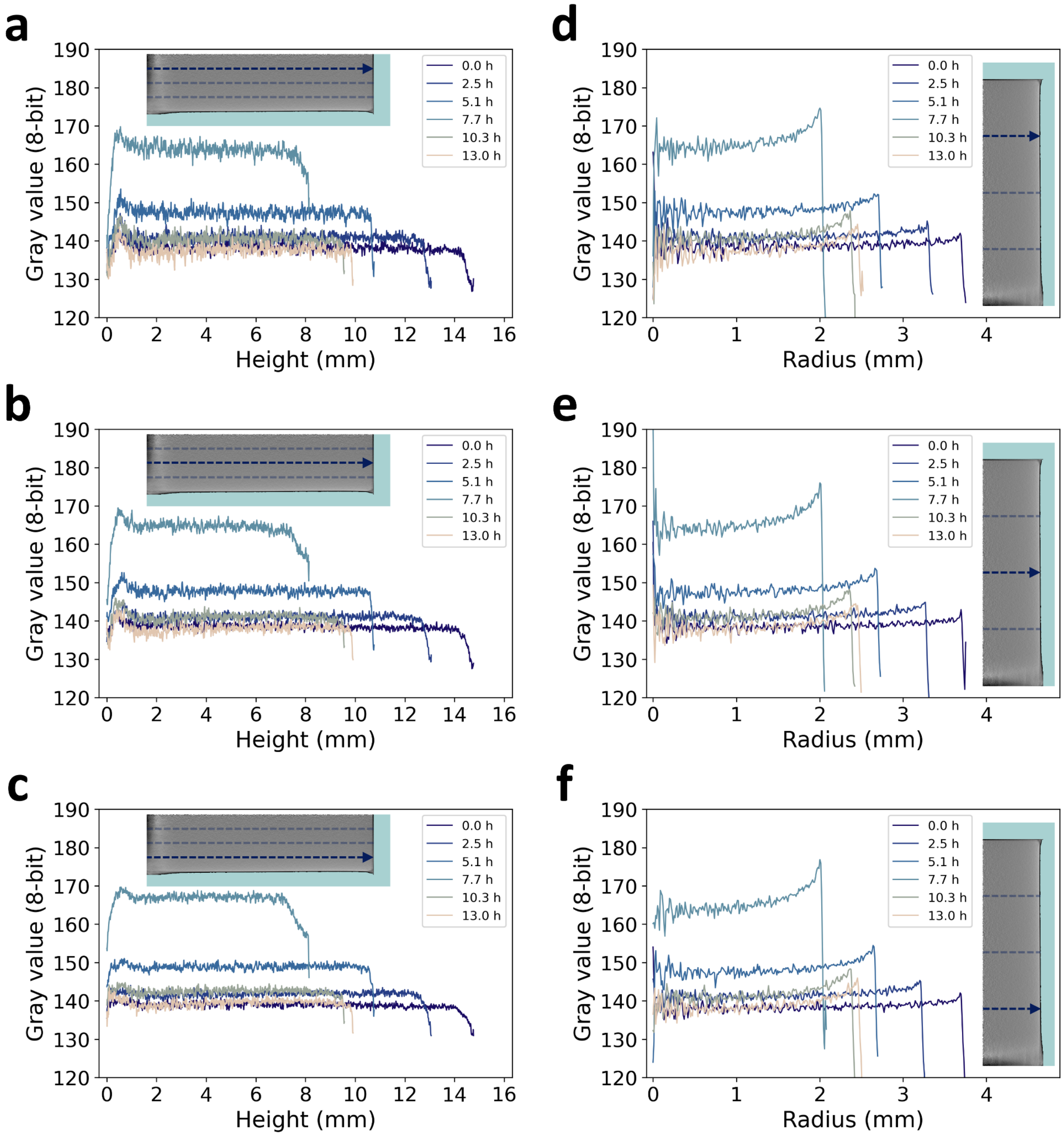

Figure S22. Gray value profiles extracted from the GHR maps of sample M4. Each panel presents the gray value profiles at six drying stages. Each panel includes a GHR map as an inset with an arrow illustrating the region where the gray value profiles are extracted. The regions (lines) are fixed at a relative height or relative radius regarding to the maximum height or radius at each drying stage. (a-c) Gray value profiles along the height of the gel at a relative radius of (a) 0.25, (b) 0.5 and (c) 0.75. (d-f) Gray value profiles along the radius of the gel at a relative height of (d) 0.75, (e) 0.5 and (f) 0.25.

The gray values remained relatively constant across the gel's height throughout drying, suggesting a homogeneous composition (Figure S22a-c). Variations were only observed during the spring-back effect (visible in Figure S22c at 7 – 8 mm and 7.6 h of drying) and at the edges of the sample, regardless of the radial coordinate. The small peak at a height of ca. 0.5 mm was seemingly caused by under-sampling artifacts in the masked slices [5] due to the low number of projections taken and the proximity of the sample with the drying chamber. This region with locally higher gray values can clearly be seen in the GHR maps

(Figure S9 – Figure S11). The lower gray values before the peak at the bottom of the gel (at a height of 0 mm), as well as the lower gray values at the top of the gel (Figure S22a-c) were due to imperfect segmentation at the edges which included some of the background in the ROIs.

Across the gel's radius, the gray values followed an exponential growth with the radius regardless of the vertical coordinate (Figure S22d-f). After reaching a maximum near the edge of the sample, the gray values decreased due to imperfect segmentation that included some of the background in the masked slices. The exponential dependency of the gray values on the gel's radius was coherent with beam hardening artifacts in cylindrical samples [6]. Beam hardening artifacts are caused by a higher attenuation of the soft X-rays in the center of the sample than at its periphery with polychromatic radiations. Beam hardening was expected in our in operando experiments given the relatively high voltage (135 kV) and the absence of a filter. The change in amplitude and curvature of the radial variations was quantified throughout drying by fitting the gray value profiles to compare the variations with a simplified beam hardening model.

RAC radial profiles were extracted from the MR map of sample M4 at each scan number. The edges effect were excluded by considering the profiles until the maximum of the RAC values. The RAC radial profiles were then fitted with an exponential function defined as $f(r) = Ae^{r/B} + C$, where *r* is the gel's radius in mm and *A*, *B* and *C* are fitting parameters. Figure S23a shows the fitted radial profiles at selected drying stages. The amplitude of the radial variations was quantified by δ_μ, defined as the difference of the RAC at the outer radius of the gel and the RAC at the center of the gel:

$$\delta_\mu = A\left(e^{r_{\max}/B} - 1\right), \tag{68}$$

where $r_{\max}$ is the maximum radius of the gel. The curvature was quantified with the inverse of the B parameter, the lower B the more the profiles were curved, compared to flat profiles as $B \to \infty$. The fitting of the radial profiles generated 141 values of δ_μ and B shown in Figure S23b and Figure S23c, respectively along the average diameter of the gel. δ_μ was inversely proportional to the gel diameter: the amplitude of the radial variations increased as the gel diameter decreased. The maximum amplitude corresponded to the maximum shrinkage and the steep drop afterwards was likely caused by the heterogeneous spring-back of the gel. A similar behavior was observed for the curvature of the spatial variations. The evolution of the B parameter throughout drying was comparable to the gel diameter with a higher curvature the smaller the diameter was. Identical conclusions were made from spatial variability analyses on samples M1, M2 and M4. This procedure was also performed on the hr-maps and provided similar results in terms of amplitude, but the curvature could not be accurately determined due to the noise in the GHR maps.

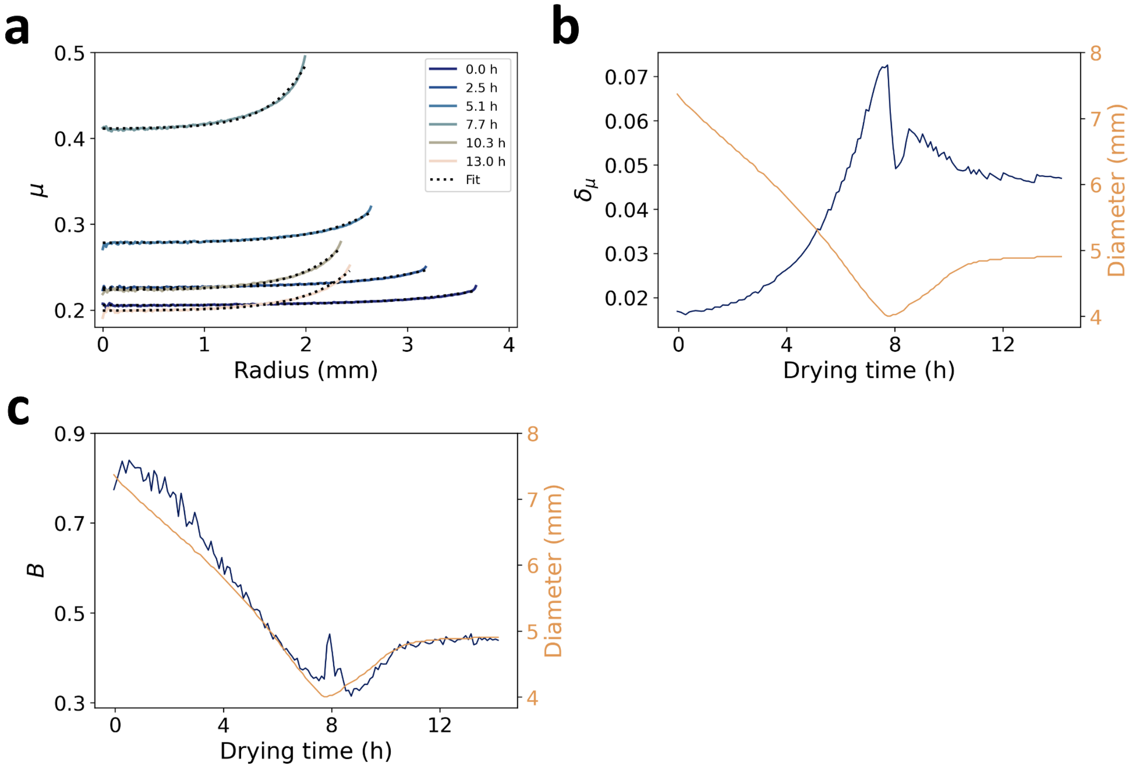

Figure S23. (a) Example of the fitting results on the radial profiles extracted from the MR maps of sample M4 at six drying stages. (b) Amplitude of the gray value variations: δ_μ parameter (blue) and average diameter of the gel (orange). (c) Curvature of the gray value variations (blue) and average diameter of the gel (orange). The smaller B, the more curved the radial profiles.

The correlation between the amplitude (δ_μ) and the curvature ($1/B$) of the variations with the diameter of the gel was consistent with beam hardening. Considering a large cylindrical sample, most of the soft X-rays are absorbed except a relatively small portion at the sample edges resulting in virtually higher RAC in these regions. In a smaller cylindrical sample of the same material, the difference in the extent of soft X-rays absorbed in the center and at the edge is more pronounced due to the exponential dependency of X-ray transmission with the sample thickness[1] [7], resulting in stronger beam hardening and in increased amplitude and curvature of the variations in the gray values and RAC along the gel radius.

Besides beam hardening artifacts, a potential gradient of the hexane composition along the radial direction of the gel could also generate the radial variations observed in Figure S22d-f and Figure S23a. However, the amplitude of the variations would then vanish in the dry gel, which was not the case in our experiments. Additionally, the value of δ_μ was inversely proportional to the diameter of the gel and not on the hexane content. Eventual radial variations caused by a different hexane content should decrease in amplitude as

[1] The transmission of a monochromatic X-ray beam through a materials of attenuation coefficient μ and thickness d is given by Beer-Lambert's law: $T = I/I_0 = e^{-\mu d}$, where T is the transmission and I and I_0 are the transmitted and initial intensity of the X-ray beam, respectively.

the hexane evaporates, whereas δ_μ had the same value after 6 h of drying and at the end of drying (Figure S23b). On the other hand, heterogeneities of the silica skeleton composition in the gel could produce variations of the gray values and RAC along the gel radius. A denser shell of silica skeleton could have formed in the samples during gelation in the molds. Such variations would not vanish in the dry gel and would be consistent with the increase in curvature with the gel diameter. While this hypothesis could not be ruled out, the significant increase in the amplitude of the variations between the maximum shrinkage and the start of drying could not be solely explained by a higher content of skeleton at the edge of the gel.

The spatial variability analysis leaned towards the occurrence of beam hardening to explain the observed variations along the gel radius, with possible effects related to a heterogeneous composition of the gels. Given those remarks, the evaluation of the evaporation mechanisms was performed on the GH map and the derived vertical volume fraction maps, as beam hardening artifacts were not observed along the gel height.

Radial variations in the volume fraction maps. Upon modeling the phase composition from the GHR and GR maps, similar radial variations as the ones attributed to beam hardening appeared in the corresponding volume fraction maps. As a result, the phase composition of the gel seemed to vary along its radius. This can be slightly seen in the HEXR and AIRR maps shortly before the maximum shrinkage of sample M4 in the main text and in Figure S12 – Figure S14 for the other samples. Figure S24 shows the radial volume fraction profiles extracted from the HEXR, SKELR and AIRR maps at three selected drying stages, where variations along the gel radius could clearly be seen. These were attributed to the propagation of beam hardening artifacts present in the gray value maps. It was worth noting that the amplitude and curvature of the variations observed in the HEXR, SKELR and AIRR radial maps were not directly proportional to the RAC variations shown in Figure S23. This was due to the way the drying model was defined: the quantity $\mu_{skel} f_{skel,i,j,k}$ was calculated from the µCT scans of the dry gel and interpolated and rescaled to the other scans (SI2).

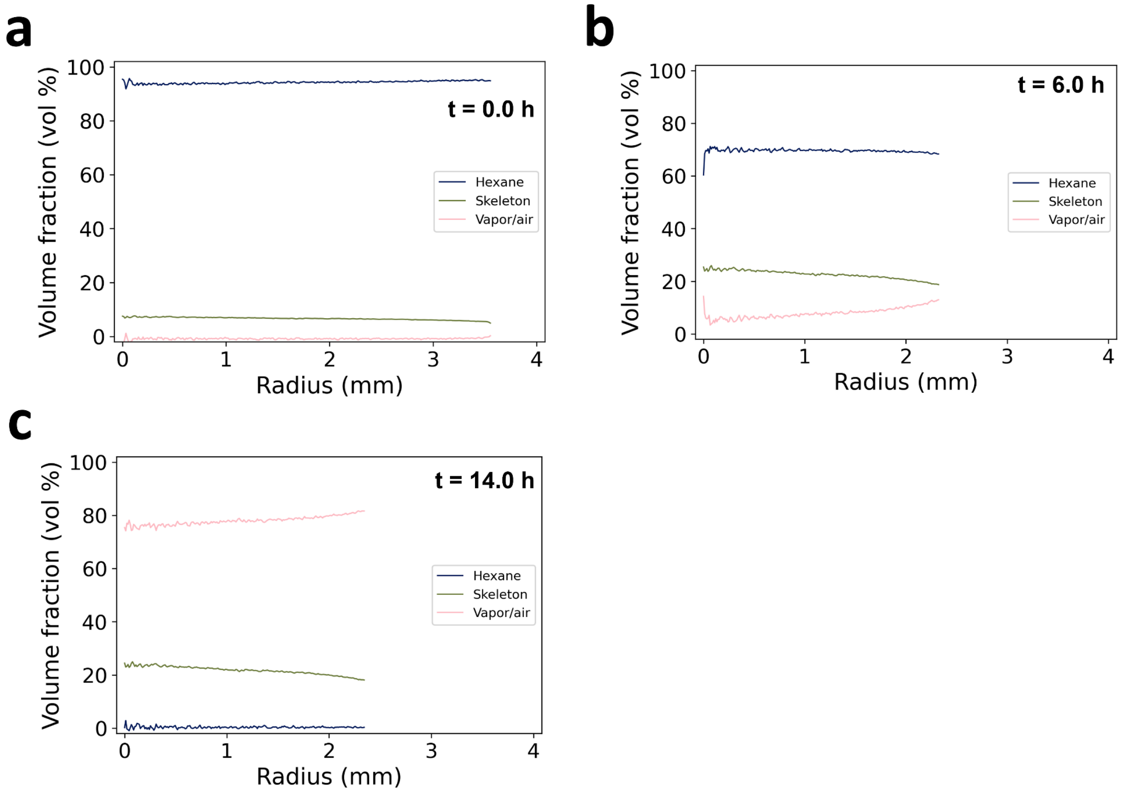

Figure S24. Hexane, skeleton and vapor/air radial profiles extracted from the HEXR, SKELR and AIRR maps of sample M4 at three selected drying stages: (a) 0.0 h, (b) 6.0 h and (c) 14.0 h. The time is indicated in each panel on the top right corner.

Azimuthal variations. This section presents the spatial variability analysis on the gray values along the azimuth of the cylindrical samples to evaluate potential heterogeneities in the gel composition during drying. To do so, selected masked slices were integrated radially with a custom Python script to produce azimuthal gray value profiles. For each scan number, a masked slice was selected at a given relative height of the cylinder. The center of each masked slice was defined as the center of mass as described in SI1. The pixels in the masked slices were separated into 100 bins depending on their azimuth from 0 to 2π and were radially integrated. The resulting gray value maps were defined as the azimuthal maps $g_{\chi,k}$, where χ stands for the azimuthal range with $0 \leq \chi \leq 99$ and k for the scan number. Rather than evaluating the azimuthal map, the difference of the gray values compared to the average at a given scan was computed:

$$\delta_{\chi,k} = g_{\chi,k} - \frac{1}{100}\sum_{\chi} g_{\chi,k}, \tag{69}$$

where $\delta_{\chi,k}$ is also a map. Figure S25 shows the final results at three relative heights: 0.25, 0.5 and 0.75.

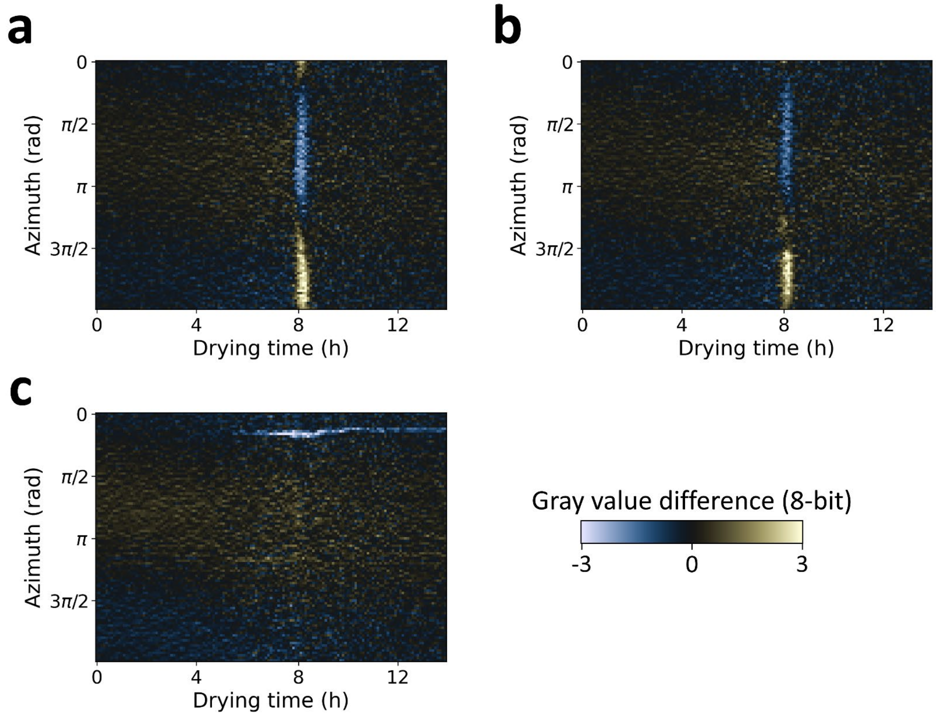

Figure S25. $\delta_{\chi,k}$ **maps (gray value difference) of sample M4 throughout drying. The maps in panels (a-c) refer to the radial integration performed at a relative height of (a) 0.25 (near the bottom), (b) 0.5 and (c) 0.75 (near the top) in the gels. Each panel share the same color scale which is indicated at the bottom right of the figure. Each map consists in 100x141 data points and the horizontal axis is linearly interpolated in time.**

Slight variations in the gray values (+/- 1 gray value) were consistently observed along the azimuth regardless of the drying stage and relative height. The gray values were higher than the average between $\pi/2$ and π and lower between $3\pi/2$ and 2π. Similar variations were observed in the other samples at different angles, and the minimum of the variations systematically occurred in the regions the closest to the wall of the drying chamber while the maximum occurred at the opposite location (Figure S26). Those spatial variations were attributed to reconstruction artifacts but could not be clearly identified to a specific µCT artifact. A combination of undersampling and beam hardening artifacts may have created these variations. Nevertheless, the established dependency of the azimuthal variations with the proximity of the gel to the chamber suggested that they did not originate from heterogeneities in the composition of the gels. Stronger azimuthal variations appeared shortly after the maximum shrinkage in samples M2, M4 and M5 (Figure S25a,b and Figure S26). The time at which the variations increased corresponded to the time where the drying front reached the relative height of the gel at which the azimuthal maps were produced, which suggested that the spring-back effect was slightly heterogeneous along the azimuthal direction of the gels. This feature was not observed in sample M1, which may be related to the fact that M1 stayed relatively well centered during drying. Sample M5 moved during drying, resulting in a change of direction of the variations before and after maximum shrinkage (Figure S26d).

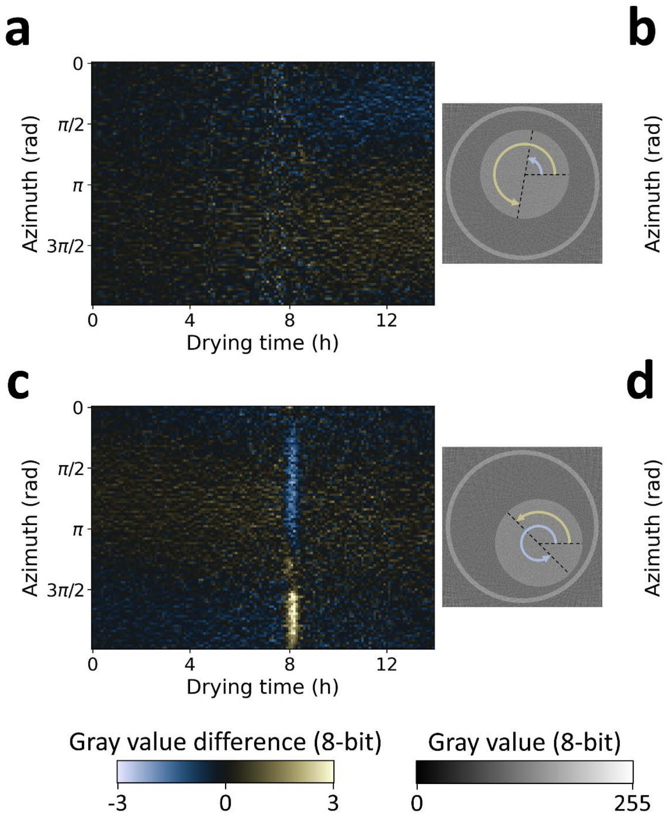

Figure S26. $\delta_{\chi,k}$ **maps (gray value difference) at a relative height of 0.5 throughout drying. The maps in panels (a), (b) (c) and (d) refer to the samples M1, M2, M4 and M5, respectively. Each panel contains an inset on the right of the map showing the masked slice at the relative height of 0.5 at the beginning of drying. The angle corresponding to the maximum (yellow) and minimum (blue) of** $\delta_{\chi,k}$ **are depicted on the masked slice with arrows. Each map consists in 100x141 data points and the horizontal axis was linearly interpolated in time.**

SI6: Comparative analysis of the Quantitative Imaging Approaches

This section reports a comparative analysis between the quantitative imaging results from this study and from the global quantitative imaging procedure from ref [2] to evaluate the reliability of the approach presented here. The gray value and volume fraction maps from the three reduction procedures were integrated along their corresponding spatial domains to calculate a spatially averaged quantity representative of the gel throughout drying. The integration of the volume fraction maps was performed over a partial domain of the corresponding maps and was consistent with the global quantitative imaging approach. For a given map $M_{i,j,k}$, the integration was defined as:

$$\bar{M}_k = \frac{1}{N_{\Omega'_k}} \sum_{(i,j)\in\Omega'_k} M_{i,j,k} \cdot n_j \tag{70}$$

Where $\bar{M}_k$ is the average value of the map $M_{i,j,k}$ integrated over the partial domain Ω'_k, with $M_{i,j,k}$ a GHR, HEXHR, SKELHR or AIRHR map, $N_{\Omega'_k}$ is the number of pixels within Ω'_k and n_j is a radial weight factor depending on the radial distance given by the index j. As a reminder to the reader: the GHR maps were

computed by azimuthal integration of the masked slices, thus the values in $g_{i,j,k}$ at a large index j were calculated from more pixels in the masked slices than the values at a small j. The radial weight factor n_j accounted for this and was defined as $n_j = \pi[(j+1)^2 - j^2]$. The averaging of the GH, HEXH, SKELH, AIRH and GR, HEXR, SKELR and AIRR maps was done similarly as in eq. (70). Depending on the nature of the map (i.e. on the reduction procedure used), the domains were further cropped to mitigate the contribution of the artifacts at the edges of the maps originating from improper segmentation. Those edges effects did not have a large impact on the integration of the gray value maps because the variations were in the order of 10-20 gray values out of 255 over a relatively small domain. However, upon calculating the volume fraction maps from the gray value maps, these variations were significantly amplified sometimes resulting in volume fraction at the edges of +/- 500 %, which were not representative of the state of the gel.

Figure S27 – Figure S30 show the average quantities calculated in this study for samples M1, M2, M4 and M5, respectively (referred to as local maps) along with the results of the global quantitative imaging procedure. The gray values were in good agreement in all samples and all three data reduction procedures between the local and global approaches, besides a slight shift of the maximum gray value. The hexane volume fraction evolution was also consistent between both workflows, although some variations were observed at and shortly after the maximum shrinkage, especially in the HEXR maps (e.g. in Figure S27d). A similar feature was also observed in Figure 7 in the main text. The content of vapor/air showed comparable variations at the maximum shrinkage. Overall, the average skeleton volume fraction profiles from the local approach were systematically higher than the one calculated in the global approach, while the average vapor/air profiles from the local approach were lower than in the global one. It was not possible to link the differences between the two workflows to a specific aspect of the two quantitative imaging approaches, as they were fundamentally different. Whereas the global quantitative imaging approach consisted mainly of two steps: integration of the gray values and calculations, the local quantitative imaging approach in this work consisted in: partial integration of the gray values (SI1), bilinear interpolation and rescaling (SI3), calculations (SI2) and partial integration for the comparative analysis (SI6). It could notably not be ruled out that the averaging procedure described here was responsible for the observed variations. Nonetheless, the evolution of the average local volume fraction throughout drying appeared consistent with our previous study.

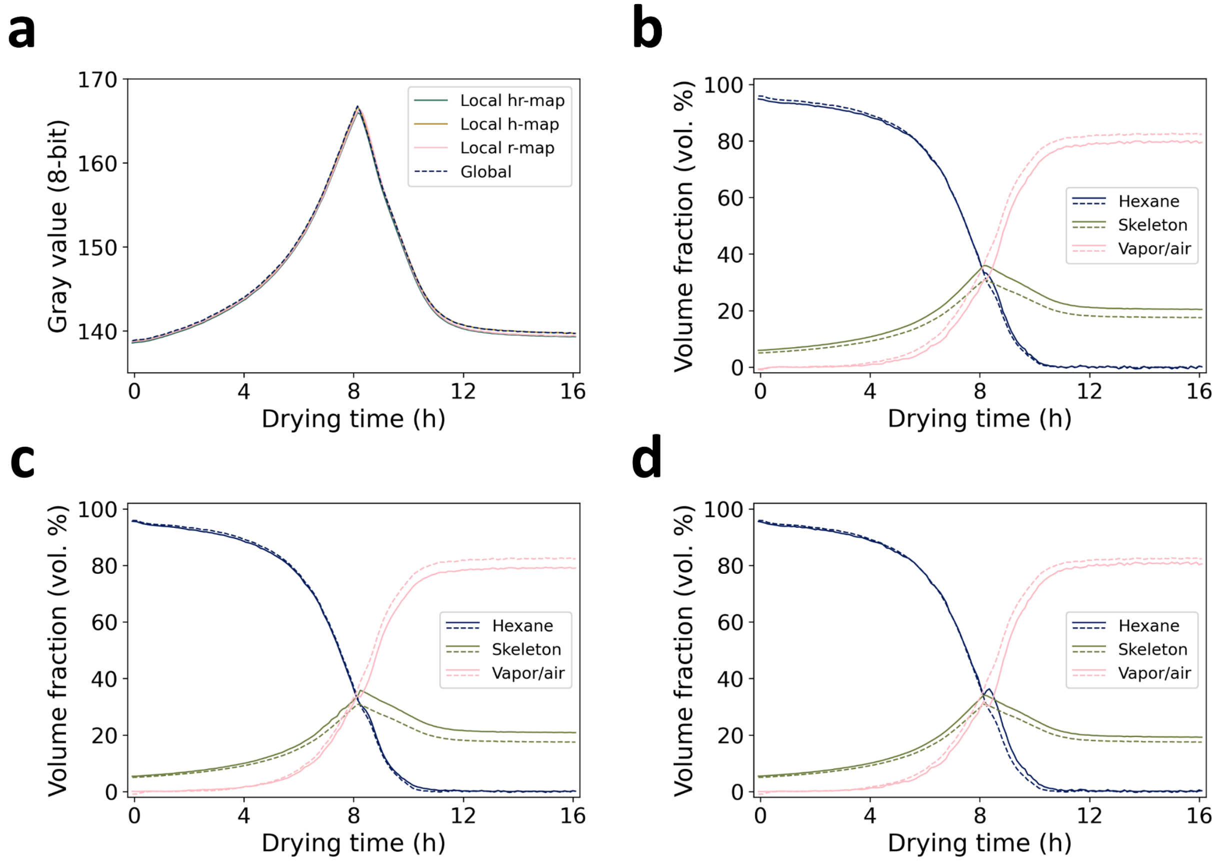

Figure S27. Comparison between the local and global quantitative imaging approaches in sample M1. (a) Global gray values and averaged local gray value in the three reduction procedures. (b-d) Global volume fraction (dashed lines) and averaged local volume fraction (full lines) of the three phases for the azimuthal integration (b), slice integration (c) and azimuthal + vertical integration (d) reduction procedures.

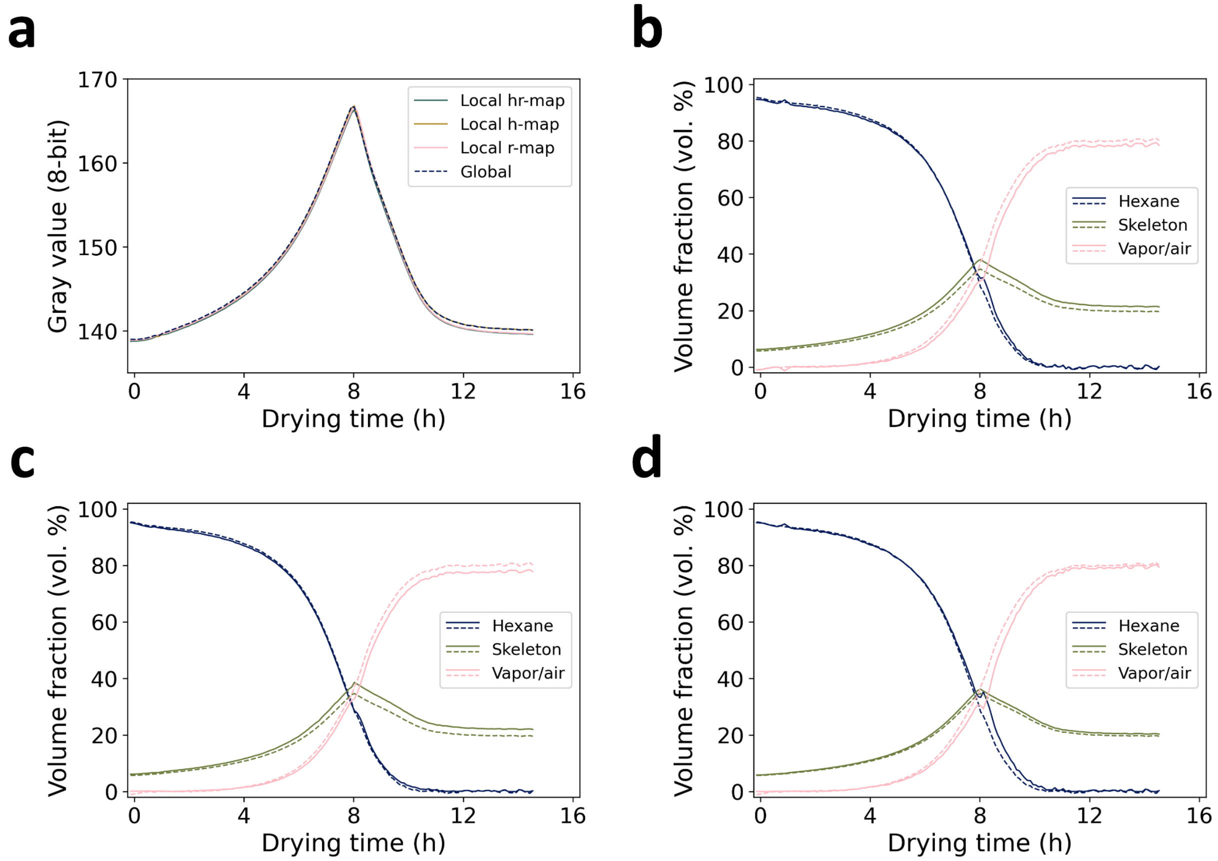

Figure S28. Comparison between the local and global quantitative imaging approaches in sample M2. (a) Global gray values and averaged local gray value in the three reduction procedures. (b-d) Global volume fraction (dashed lines) and averaged local volume fraction (full lines) of the three phases for the azimuthal integration (b), slice integration (c) and azimuthal + vertical integration (d) reduction procedures.

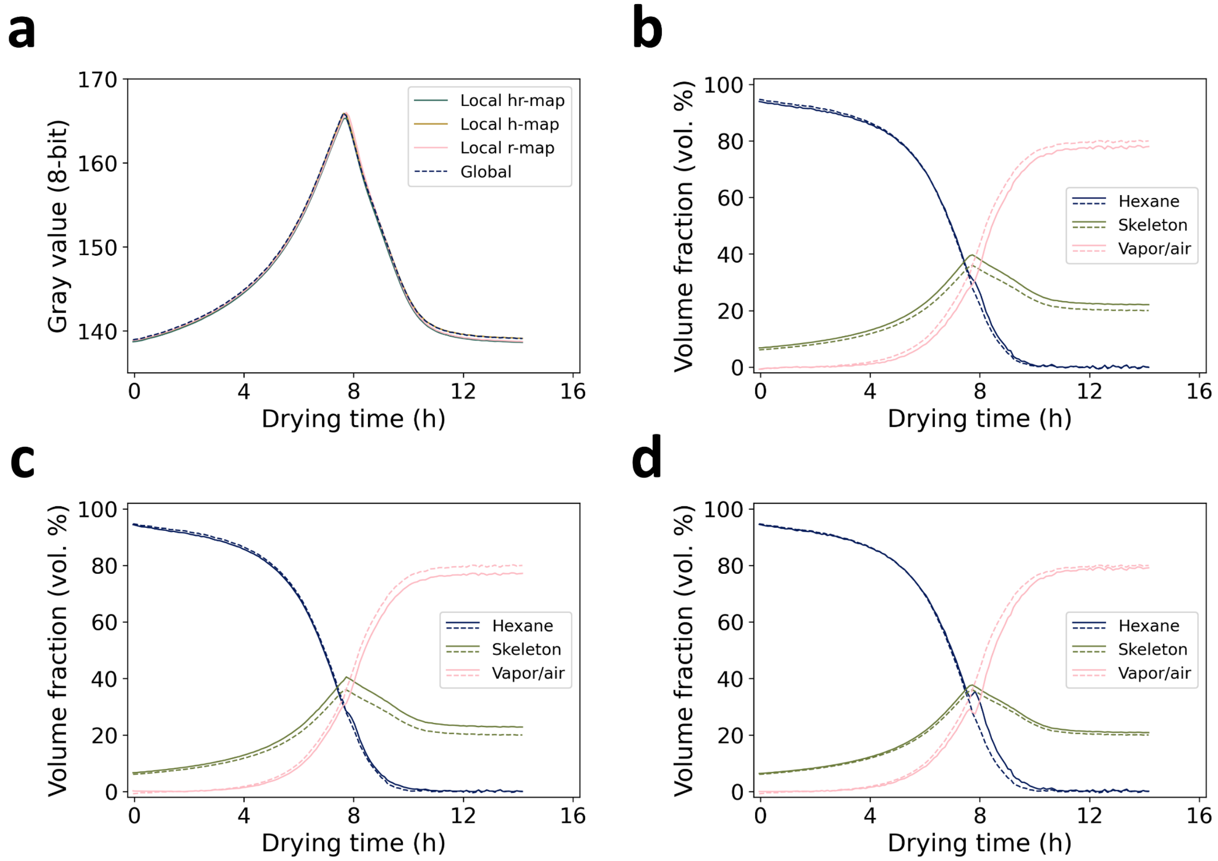

Figure S29. Comparison between the local and global quantitative imaging approaches in sample M4. (a) Global gray values and averaged local gray value in the three reduction procedures. (b-d) Global volume fraction (dashed lines) and averaged local volume fraction (full lines) of the three phases for the azimuthal integration (b), slice integration (c) and azimuthal + vertical integration (d) reduction procedures.

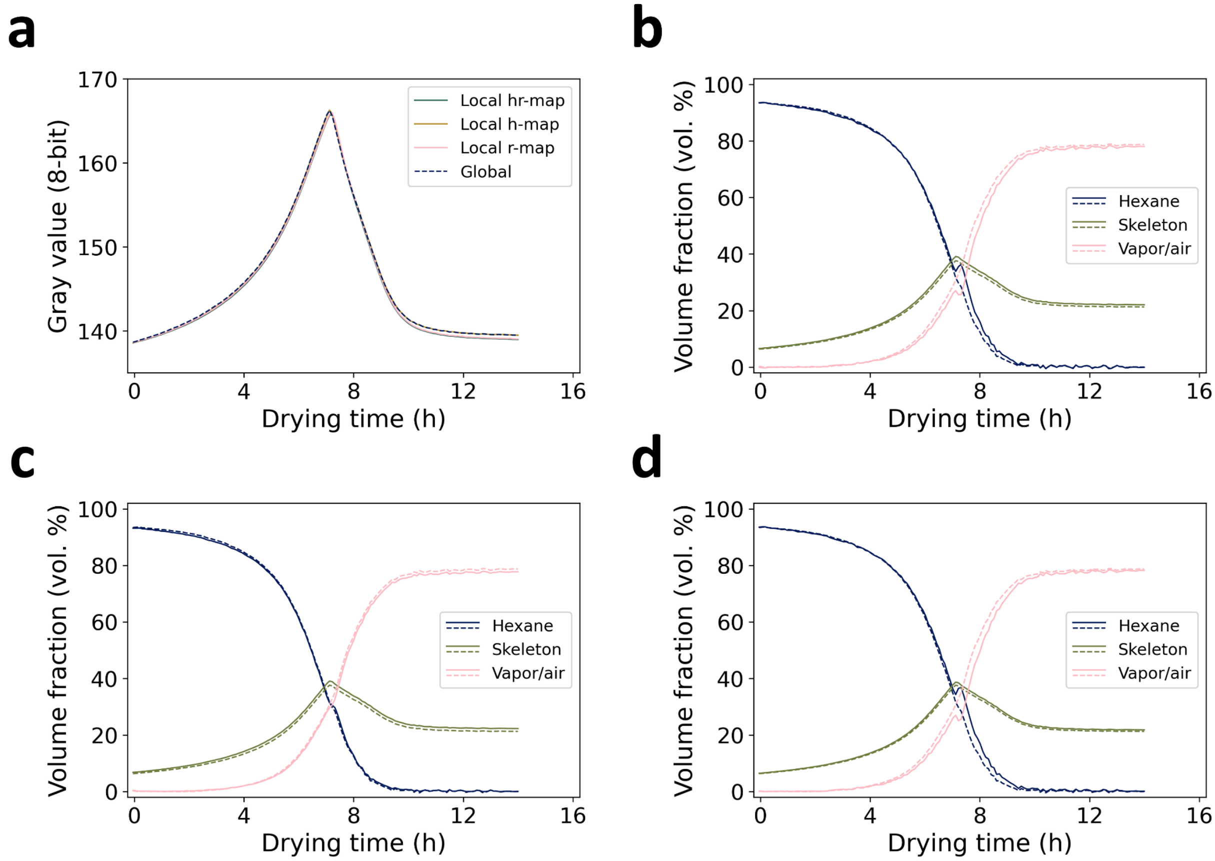

Figure S30. Comparison between the local and global quantitative imaging approaches in sample M5. (a) Global gray values and averaged local gray value in the three reduction procedures. (b-d) Global volume fraction (dashed lines) and averaged local volume fraction (full lines) of the three phases for the azimuthal integration (b), slice integration (c) and azimuthal + vertical integration (d) reduction procedures.

Figure S31

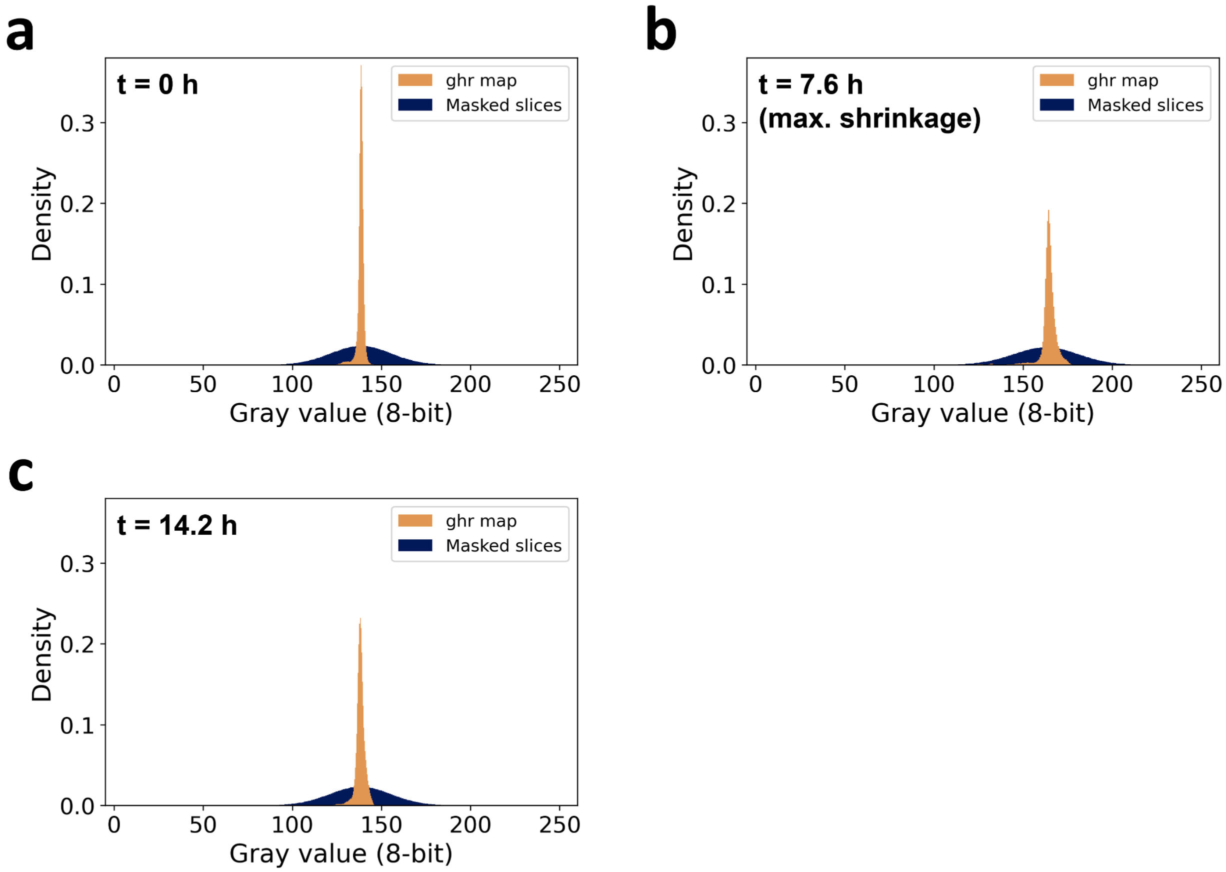

Figure S31. Gray value density histograms of a GHR map (orange) and of all the masked slices (blue) at three selected drying stages: (a) t = 0 h, (b) t = 7.6 h (maximum shrinkage) and (c) t = 14.2 h. Upon azimuthal integration, the distribution of the gray values became narrower.

References

[1] “DIPlib” [Computer software]. 2023. https://github.com/DIPlib/diplib.

[2] J. Gonthier *et al.*, “In Operando μCT Imaging of Silylated Silica Aerogels during Ambient Pressure Drying and Spring-Back,” *Chem. Mater.*, vol. 35, no. 18, pp. 7683–7693, Sep. 2023, doi: 10.1021/acs.chemmater.3c01451.

[3] Object Research Systems (ORS) Inc. “Dragonfly” [Computer software]. (version 2022.2). 2022. http://www.theobjects.com/dragonfly.

[4] J. Schindelin *et al.*, “Fiji: an open-source platform for biological-image analysis,” *Nat. Methods*, vol. 9, no. 7, pp. 676–682, 2012, doi: 10.1038/nmeth.2019.

[5] R. Schulze *et al.*, “Artefacts in CBCT: a review,” *Dentomaxillofacial Radiol.*, vol. 40, no. 5, pp. 265–273, Jul. 2011, doi: 10.1259/dmfr/30642039.

[6] G. R. Davis and J. C. Elliott, “Artefacts in X-ray microtomography of materials,” *Mater. Sci. Technol.*, vol. 22, no. 9, pp. 1011–1018, Sep. 2006, doi: 10.1179/174328406X114117.

[7] P. J. Withers *et al.*, “X-ray computed tomography,” *Nat. Rev. Methods Prim.*, vol. 1, no. 1, p. 18, 2021, doi: 10.1038/s43586-021-00015-4.
